# Principles Governing A-to-I RNA Editing in the Breast Cancer Transcriptome

**DOI:** 10.1016/j.celrep.2015.09.032

**Published:** 2015-10-01

**Authors:** Debora Fumagalli, David Gacquer, Françoise Rothé, Anne Lefort, Frederick Libert, David Brown, Naima Kheddoumi, Adam Shlien, Tomasz Konopka, Roberto Salgado, Denis Larsimont, Kornelia Polyak, Karen Willard-Gallo, Christine Desmedt, Martine Piccart, Marc Abramowicz, Peter J. Campbell, Christos Sotiriou, Vincent Detours

**Affiliations:** 1 Breast Cancer Translational Research Laboratory, Jules Bordet Institute, Université Libre de Bruxelles (ULB), Boulevard de Waterloo, 125-1000 Brussels, Belgium; 2 IRIBHM, Université Libre de Bruxelles (ULB), Route de Lennik, 808-1070 Brussels, Belgium; 3 WELBIO, Route de Lennik, 808-1070 Brussels, Belgium; 4 Cancer Genome Project, Wellcome Trust Sanger Institute, Wellcome Trust Genome Campus, Hinxton, Cambridgeshire CB10 1SA, UK; 5 Department of Pathology, Jules Bordet Institute, Université Libre de Bruxelles (ULB), Boulevard de Waterloo, 125-1000 Brussels, Belgium; 6 Department of Medical Oncology, Dana-Farber Cancer Institute, 450 Brookline Avenue, Boston, MA 02215, USA; 7 Molecular Immunology Unit, Jules Bordet Institute, Université Libre de Bruxelles (ULB), Boulevard de Waterloo, 125-1000 Brussels, Belgium; 8 Department of Medicine, Jules Bordet Institute, Université Libre de Bruxelles (ULB), Boulevard de Waterloo, 125-1000 Brussels, Belgium; 9 Department of Genetics, Hôpital Erasme, Route de Lennik, 808-1070 Brussels, Belgium

## Abstract

Little is known about how RNA editing operates in cancer. Transcriptome analysis of 68 normal and cancerous breast tissues revealed that the editing enzyme ADAR acts uniformly, on the same loci, across tissues. In controlled ADAR expression experiments, the editing frequency increased at all loci with ADAR expression levels according to the logistic model. Loci-specific “editabilities,” i.e., propensities to be edited by ADAR, were quantifiable by fitting the logistic function to dose-response data. The editing frequency was increased in tumor cells in comparison to normal controls. Type I interferon response and *ADAR* DNA copy number together explained 53% of ADAR expression variance in breast cancers. *ADAR* silencing using small hairpin RNA lentivirus transduction in breast cancer cell lines led to less cell proliferation and more apoptosis. A-to-I editing is a pervasive, yet reproducible, source of variation that is globally controlled by 1q amplification and inflammation, both of which are highly prevalent among human cancers.

## Highlights

• A-to-I editing is a major source of mRNA variability in breast and other cancers
• RNA editing is globally controlled by tumor interferon and ADAR copy number
• Both these factors are highly prevalent among human cancers
• RNA editing sites might represent a new class of therapeutic targets

## Graphical Abstract

**Figure:**
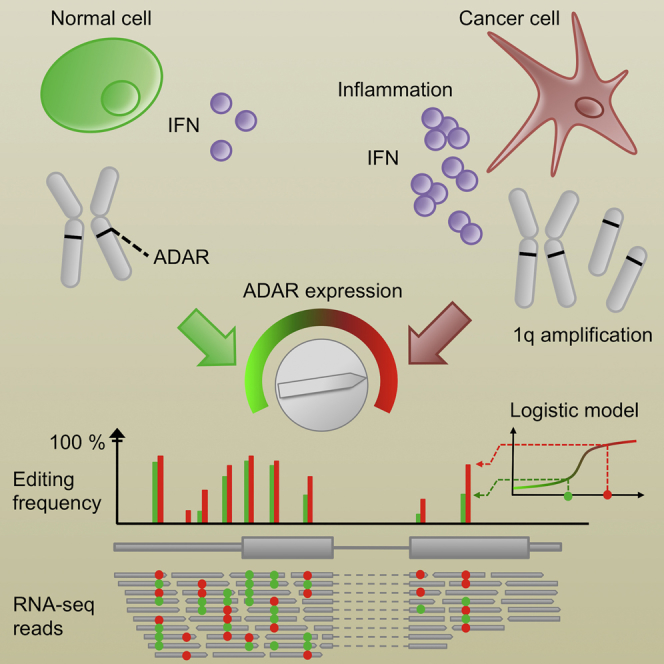

## Teaser

Fumagalli et al. identify the principles governing A-to-I editing in breast and potentially all types of cancer, demonstrating that A-to-I editing is a pervasive source of transcriptome variation that is mainly controlled by two factors, 1q amplification and inflammation, both of which are highly prevalent among human cancers.

## Introduction

Although intense effort is currently being dedicated to cancer genome sequencing, comparatively little attention has been devoted at understanding how faithful RNA sequences are to the DNA sequences from which they were derived. mRNA is the target of a series of post-transcriptional modifications that can affect its structure and stability, one of the most relevant being RNA editing (Bass, 2002, Levanon et al., 2004, Nishikura, 2010). The most common form of RNA editing in humans, the A-to-I type, is catalyzed by the adenosine deaminases that act on RNA (ADARs) family of enzymes, which bind double-stranded RNA (dsRNA) and turn adenosines into inosines at precise positions (Bass, 2002, Nishikura, 2010). Inosines are subsequently interpreted as guanosines by the cellular transcription machinery. ADAR enzymes are essential in mammals (Higuchi et al., 2000, Wang et al., 2000) and exist in three forms: ADAR (also known as ADAR1), which is ubiquitous and has two isoforms—p110 is constitutive and p150 is inducible; ADARB1 (also known as ADAR2), principally expressed in the brain; and ADARB2 (also known as ADAR3), which contrary to ADAR and ADARB1 seems to be enzymatically inactive (Chen et al., 2000, Savva et al., 2012).

A-to-I edits can profoundly influence cellular functions and regulations by altering mRNA splicing, stability, localization, and translation, and by interfering with the binding of regulatory RNAs (Athanasiadis et al., 2004, Rueter et al., 1999, Wang et al., 2013). In addition to mRNA, ADAR can target non-coding RNAs such as micro-RNAs (miRNAs), small-interfering RNAs (siRNAs), and long non-coding RNAs (lncRNAs), affecting both their structure and activities (Blow et al., 2006, Hundley and Bass, 2010, Kapusta et al., 2013, Kawahara et al., 2007). A-to-I editing has been shown to occur predominantly in highly repetitive *Alu* sequences, likely because their frequency (>10^6^) in the human genome makes their arrangement in quasi-palindrome configurations prone to RNA duplex formation highly probable (Athanasiadis et al., 2004, Bazak et al., 2014a, Kim et al., 2004, Levanon et al., 2004). High-throughput sequencing studies suggest that tens of thousands to millions of positions are targeted by A-to-I editing in the human transcriptome (Bahn et al., 2012, Ju et al., 2011, Li et al., 2009, Park et al., 2012, Peng et al., 2012, Ramaswami et al., 2012, Ramaswami et al., 2013), and a recent publication reports that potentially all adenosines in specific *Alu* repeats undergo A-to-I editing (Bazak et al., 2014b).

Currently, a limited number of studies on A-to-I RNA editing in cancer have been published, with the findings pointing to a diversity of effects. For example, in brain cancer, editing inhibits cell growth and is reduced in glioma (Maas et al., 2001, Paz et al., 2007) and pediatric astrocytoma (Cenci et al., 2008). In contrast, A-to-I editing increases during chronic myeloid leukemia progression (Jiang et al., 2013). In hepatocellular carcinoma, A-to-I editing of the antizyme inhibitor 1 (*AZIN1*) increases and neutralizes a key inhibitor of the polyamine synthesis pathway, thereby promoting proliferation in vitro and increasing tumor initiation and volume in a mouse xenograft model (Chen et al., 2013). The studies published so far included a small number of samples—an important limit given the sheer diversity of tumor transcriptomes—and/or investigated a limited number of editing sites. Whether the edited transcripts originated from cancer cells or other cell types, e.g., immune cells, present in the tumor mass was not addressed. Hence, both the magnitude and mechanisms regulating A-to-I editing in the majority of cancers, including breast cancer (BC), remain largely unknown.

The main objective of this study was to investigate the principles governing the A-to-I editing process in BC as well as in other types of cancer.

## Results

### Detection and Validation of A-to-I Editing Sites in Breast Tissue

The extent of A-to-I RNA editing in BC was investigated by paired exome and transcriptome sequencing of a broad series of BC samples representing the principal intrinsic subtypes including 17 triple-negative (TN), 14 HER2-positive (HER2), 16 luminal A (LA), and 11 luminal B (LB) tumors (Table S1). Paired exome and transcriptome sequencing of matched, tumor-adjacent normal tissue was performed on ten cases from this series. RNA-DNA single nucleotide differences (RDDs) were called as outlined in Figure S1 (details in the Supplemental Experimental Procedures).

**Figure 1:**
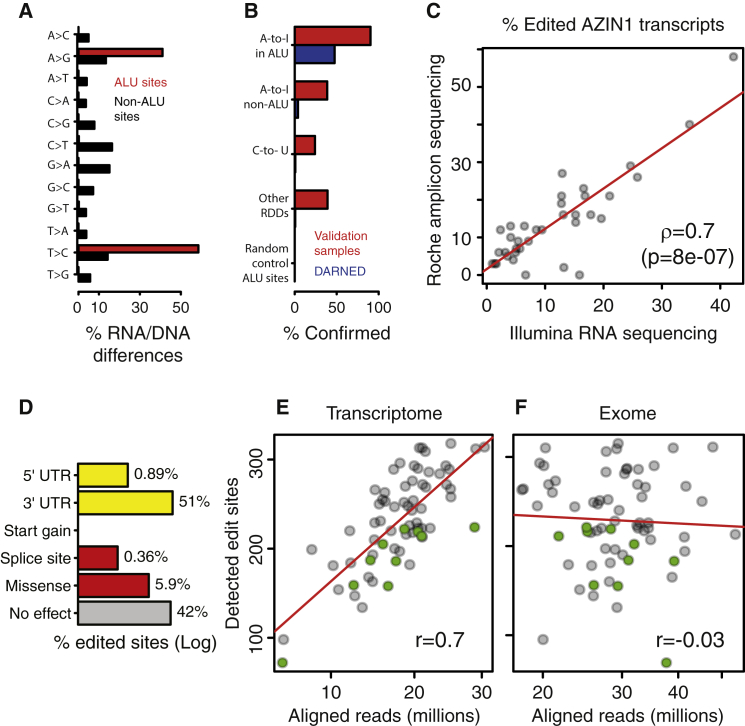
Detection of A-to-I Editing (A) Substitution frequencies of RDDs. (B) Percentage of RDDs confirmed in the validation data set, n = 15 BCs (in red), and the DARNED database (in blue). The negative control set is composed of 1,000 sites selected at random positions in randomly selected *Alu* regions. Sites in immunoglobulin (Ig) hyper-variable regions were excluded; see the Supplemental Experimental Procedures. (C) Each dot represents a sample for which the frequency of edited AZIN1 transcripts has been measured with Illumina full transcriptome sequencing (x axis) and Roche FLX amplicon sequencing (y axis). ρ denotes the Spearman’s correlation. (D) Distribution of the 560 edited sites into functional categories. (E and F) Number of detected *Alu* A-to-I sites as a function of transcriptome and exome coverages, respectively. Green dots represent tumor-matched normal samples.

Overall, we detected 16,027 RDDs in one or more samples, with all possible base changes represented (Figure 1A). Among these, 560 RDDs were located in *Alu* regions, and all were of the A-to-I type (Figure 1A; Table S2), consistent with the notion that A-to-I editing occurs predominantly in forward-facing *Alu* forming dsRNA duplexes processed by ADAR. Forty-seven percent of the A-to-I *Alu* RDDs were present in the DARNED RNA editing site database (Kiran and Baranov, 2010). In contrast, only 2.5% of A-to-I, non-*Alu* RDDs and 0.6% of non A-to-I RDDs were found in the DARNED database (Figure 1B).

Breast tissue is not well represented in the studies covered by the DARNED database. Given that gene expression and RNA editing frequency (defined for each sample as the ratio of the number of RNA sequencing (RNA-seq) reads documenting the non-reference base relative to the total number of reads covering the site) could be regulated in a tissue specific manner, we further validated our findings in an independent breast series. This independent validation series included 15 BC samples with paired transcriptome and full genome sequencing data from the Sanger Institute. The genomic coordinates of our putative RDDs and the coordinates of 1,000 random *Alu* positions were sent to the Sanger Institute without any additional information. This blind test—based on an independent RDD detection pipeline (Supplemental Experimental Procedures)—confirmed 90% of the *Alu* RDDs, while only one of the 1,000 random *Alu* sites was detected in the validation series. Beyond *Alu*, overlap with the validation series was below 40% (Figure 1B). Given the low confirmation rate of RDDs located outside of *Alu* regions in both the DARNED database and the independent validation series, and that the majority of human editing events are A-to-I detected in *Alu* repeats (Athanasiadis et al., 2004, Bazak et al., 2014b, Kim et al., 2004, Levanon et al., 2004), our subsequent analyses focused exclusively on the subset of A-to-I RDDs located in *Alu* sequences. Since several works have reported the editing of *AZIN1*, this target was also included in our analyses (Chen et al., 2013, Ju et al., 2011, Li et al., 2009, Li et al., 2011, Peng et al., 2012, Qin et al., 2014, Ramaswami et al., 2012, Shah et al., 2009).

To evaluate the accuracy of edited transcript frequencies measured in our full transcriptome data, we generated amplicons of the *AZIN1* editing site region for 36 samples that were then analyzed by an independent sequencing technology (Roche FLX sequencer). The edit frequencies measured from full transcriptome and amplicon sequencing were remarkably consistent (Figure 1C) and thereby validated the accuracy of these estimations.

The distribution of A-to-I within *Alu* edits according to functional effect is shown in Figure 1D; functional information for all putative and confirmed edited sites is available Table S2.

### The Apparent Size of the Editome Depends on the Transcriptome Sequencing Depth and on the Span of Sequenced Genomic Regions

Sequencing depth is a key factor in detecting single nucleotide variations (Bazak et al., 2014b), leading us to ask whether the exome and RNA sequencing depths could influence the number of detectable *Alu* edit sites. While this number was not dependent on the exome sequencing depth, it did greatly increase with the transcriptome coverage (Figures 1E and 1F; Table S3). No plateau was reached in our data set, which had a maximum coverage of ∼3 × 10^7^ reads/sample. This suggests that with higher transcriptome coverage additional A-to-I editing sites should be detectable in the breast transcriptome.

A comparison of our results and methods with previous literature is presented in Tables S4A and S4B. This analysis revealed that genome sequencing span is among the main factors limiting the RDD detection. Since our DNA sequencing covered the exome and not the entire genome, we implemented a less conservative editing detection pipeline bypassing the exome DNA comparison and focusing instead the detection of A-to-I editing on sites previously reported in the literature (Supplemental Experimental Procedures). This DNA-free pipeline detected 59,993 A-to-I editing sites. The main variable investigated in this paper, namely, the mean editing frequency, estimated from these 59,993 sites or the 560 *Alu* sites obtained with the DNA-based pipeline, was nearly identical (ρ = 0.9, p = 2 × 10^−16^). Most of the sites detected by the DNA-free pipeline were expressed in few samples (median, 14.7% of the samples; interquartile range [IQR], 4.4%–50%) and/or edited at low frequency (median, 0% of the reads; IQR, 0%–3.4%); i.e., they were of limited interest as far as correlative analysis across a significant fraction of the cohort is concerned and most probably had negligible influence on cancer progression. The number of sites dropped from 59,993 to 1,852 after filtering out positions expressed at detectable levels in <75% of the samples and not edited at a frequency >10% in any samples. By contrast, applying the same filter to the DNA-based pipeline reduced the number of sites from 560 to 455.

### More A-to-I Editing Was Found in Tumor Compared to Normal Matched Breast Tissue

**Figure 2:**
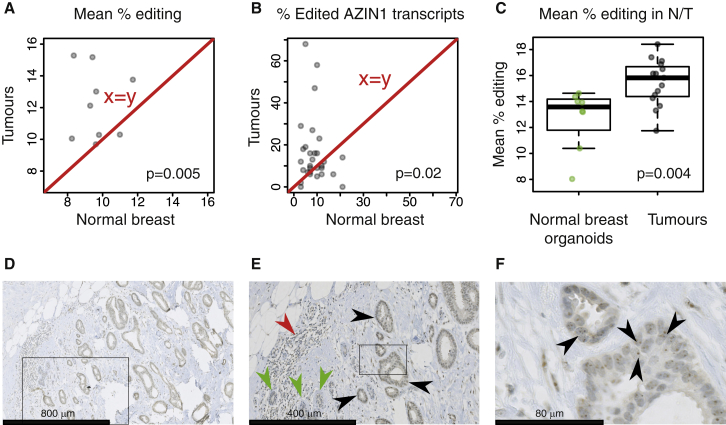
A-to-I Editing and ADAR Expression in Normal and Tumor Breast Tissue (A) Each dot represents a patient with the mean editing frequency in her normal (x axis) and her matched tumor breast tissue (y axis). (B) Same as (A), except that the AZIN1 editing frequency measured by Roche FLX amplicon sequencing is depicted. (C) The mean editing frequency of eight breast organoid cultures is compared to that of 15 breast tumors. (D) Representative ADAR staining of a luminal A tumor. (E) Zooming in (D) reveals that tumor staining (black arrows) is higher than in normal epithelium (green arrows) and lymphocytes (red arrows). (F) Zooming further in (E) reveals a higher staining of nucleoli (black arrows).

To determine whether A-to-I editing is specifically altered in BC, the mean editing frequencies across all edited sites were compared between matched normal and tumor breast tissues for ten cases where paired exome and transcriptome sequencing data were available for the normal tissue. We also compared the specific edit frequency of the *AZIN1* transcript determined by high-depth amplicon sequencing (Roche FLX sequencer) between tumor and matched normal breast tissues. The global mean editing frequency and the *AZIN1* specific editing frequency were higher in tumor compared to matched-normal breast tissues (Figures 2A and 2B; Tables S3 and S5).

Normal breast samples may contain less epithelial cells; hence, lower editing in these samples could be a trivial consequence of lower editing in non-epithelial cells (e.g., adipocytes) compared to epithelial cells. Thus, the site-averaged editing frequencies across all 560 *Alu* sites from the independent validation series (15 BCs) were compared to eight normal breast organoids (i.e., freshly isolated uncultured intact breast milk ducts). Editing was higher in tumor compared to pure normal epithelial cells (Figure 2C), which validates our findings.

### Global A-to-I Editing Is Governed by ADAR Expression and Site-Specific Editability

The general principles governing A-to-I editing in BC were investigated in multiple, matched exome-transcriptome data pairs. The ADAR family of enzymes catalyzes A-to-I editing, leading us to first determine their expression levels in normal and tumor breast tissues as well as their association with editing frequency using transcriptome sequencing data. ADAR was expressed 9-fold more than ADARB1 and >1,000-fold more than ADARB2 (p < 10^−16^, Figure S2), which was anticipated because these last two isoforms are principally expressed in the brain. Moreover, while ADAR expression was higher in tumor compared to patient-matched normal breast tissues (p = 0.005, Figure S2), an inverse borderline-significant trend was observed for ADARB1 (p = 0.1, Figure S2).

**Figure 3:**
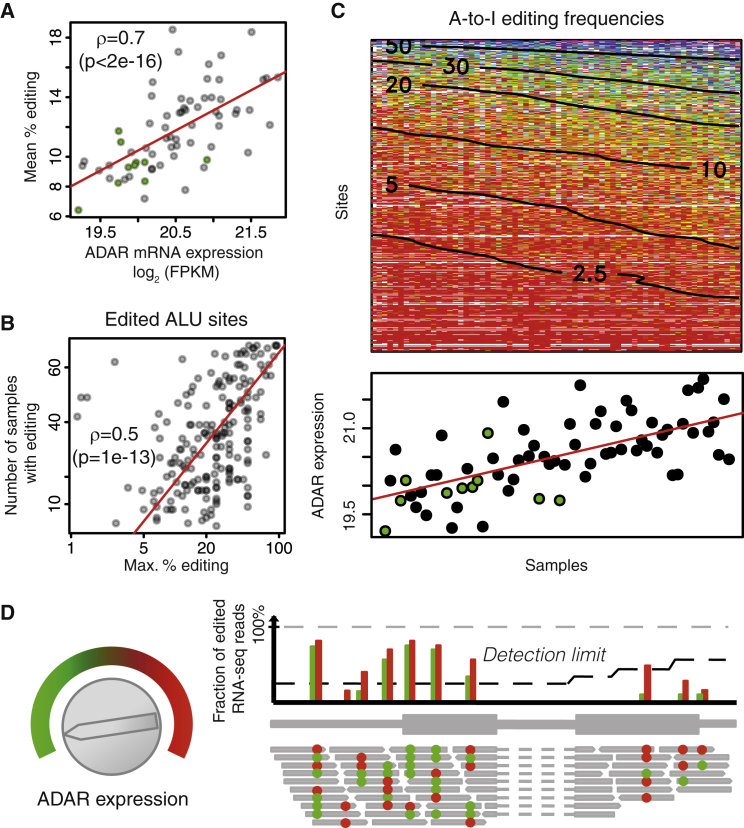
Model of A-to-I Editing (A) Each dot represents a sample with its RNA-seq-estimated ADAR expression on the x axis (in log_2_ of fragments per kilobase per million mapped reads), and its mean editing frequency across all 560 *Alu* sites on the y axis. Green dots represent tumor-matched normal samples. The RNA-seq expression of *ADAR* is highly correlated with microarrays and qRT-PCR expression (Figure S2). (B) Each dot represents an *Alu* A-to-I editing site with the maximal edit frequency across all samples on the x axis and the number of samples in which it was detectably edited on the y axis. (C) Heatmap of editing frequencies across all *Alu* A-to-I edit sites in all samples. Both are ordered by increasing (down-to-up, left-to-right) mean editing frequencies. Smoothed contour lines labels give the percentage of edited transcripts. The bottom panel shows corresponding ADAR expression. Green dots represent tumor-matched normal samples. Negative controls are presented in Figure S2. (D) Model of A-to-I editing. Turning the ADAR “expression knob” clockwise increases ADAR expression. As a result, more transcripts are edited (red dots), and the editing frequency of all editable sites increases accordingly (compare green versus red bars). Moreover, the detection limit at some sites for which editing was previously undetectable is passed. The detection limit depends on sequencing coverage, which is lower on the right-most exon. Importantly, the ranking of editing frequencies of the different sites is unaltered by *ADAR* expression.

The mean editing frequency (defined as the average editing frequency of all 560 *Alu* sites) was significantly positively correlated with ADAR mRNA expression levels (Spearman’s ρ = 0.7, p < 2 × 10^−16^; 40% of variance explained; Figure 3A; Table S3), while it was weakly anti-correlated with ADARB1 expression levels (Figure S2), as previously reported (Chen et al., 2013). The global association detected between *ADAR* mRNA expression and the mean editing frequency was also observed at individual editing sites (Figure S2; Table S2). Considering both the high levels of *ADAR* mRNA expression and its strong correlation with the mean editing frequency, our further analyses were focused on ADAR.

Editing site distribution across normal and BC tissues was investigated by plotting the maximum edit frequency for all editing sites against the number of samples where editing of these sites was detected (Figure 3B). These two variables were highly correlated indicating that if a site was highly edited in one sample, it was very likely to be edited in many other samples. This also suggested that the editing sites detected in normal tissues are also detected in matched tumor tissues and across all BC patients.

Sites and samples were then ordered by increasing mean editing frequencies, and the individual editing frequencies at all 560 *Alu* sites in all samples were displayed as a heatmap (Figure 3C; negative controls in Figure S2). This revealed that high editing frequencies were present in the samples with more editing sites and high ADAR expression. Conversely, samples with lower ADAR expression had fewer edited sites, which were edited at lower frequencies. Taken together, these data suggest a quantitative model of A-to-I editing (Figure 3D). In this model, turning up the ADAR expression “knob” leads to detectable editing at more sites and an increased editing frequency of all the editable sites. Conversely, when ADAR expression is low, editing is detectable at fewer sites and at a lower frequency. We propose that “editability,” the propensity of a position to be edited by ADAR, depends mostly upon biophysical interactions between an individual site with its surrounding RNA sequence and partnering as a duplex with ADAR. We show below how to quantitatively estimate it from dose-response data.

### Validation of the A-to-I Editing Model

**Figure 4:**
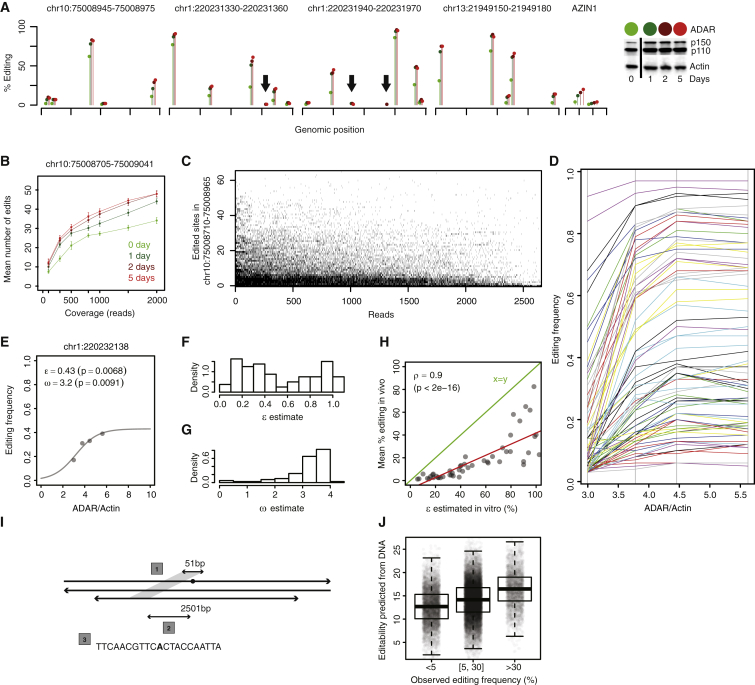
Validation of the A-to-I Editing Model and Quantitative Estimation of Site-Specific Editability (A) Effect of increasing ADAR expression in the cell line MCF7 on editing in four representative *Alu* regions and AZIN1. The full-length of sequenced regions are shown in Figure S3 for MCF7 and three more cell lines. Complete ADAR western blots quantifications underlying the color scale are provided in Figure 5E (see baseline t = 0 and IFN-α, t ∈ {1, 2, 5} days tracks) and in Figure S7. Increasing ADAR expression increases the editing frequency at all editable positions, as predicted by the model of Figure 3D. Similar results were obtained for IFN-β and IFN-γ (global, position-less, view Figure 5F). Arrows point at editing sites detectable only at higher ADAR expression in our assay. (B) Increasing sequencing coverage (x axis) or ADAR expression (color scale) increases the number of detectable editing sites (y axis). Coverage variation was implemented by down-sampling the total pool of sequencing reads, starting from 2,000×, down to 100×, and re-running the variant detection pipeline for each down-sampled alignment. Each data point is the mean of 30 down-sampling experiments. Error bars, SD. (C) Editing of individual mRNA molecules. Each black dot depicts an edited base in a given mRNA molecule. The y axis goes from 0 to 60 and corresponds to the adenosines in the ∼250-bp span that are edited in at least one of the 2,842 reads represented along on the x axis. Reads and adenosines were ordered by decreasing editing frequencies. 185 non-edited reads were omitted from the figure. (D) Dose-response curves for experiment in cell line BT474. ADAR was increased through IFN-α stimulation (as in A). We focused on 81 sites (color lines) with a baseline editing frequency >2.5% in order to avoid trivial nonlinear effects caused by lack of detection at low ADAR expression. (E) Example of a fit of the logistic model (line) to experimental data points (dots). The unit of ω is commensurate to the dimensionless ADAR relative expression and ε is the fraction of edited transcripts at saturation. (F and G) Distributions of ε and ω across the 81 sites. (H) The 81 edited sites are depicted as dots with the corresponding *ε*_*i*_ estimates derived from the BT474 cell lines on the x axis and their in vivo editing frequency on the y axis. (D)–(H) are part of a more comprehensive analysis presented in Figure S4. (I) DNA-based statistical model of editability. The model included three parameters: (1) the best Smith-Waterman global alignment score of the 51-bp sequence surrounding the editing site (green dot) within the 2,501-bp sequence surrounding the editing site on the reverse strand; (2) the distance separating the editing site from this best alignment; (3) the 20 nucleotides surrounding the editing site. These 1 + 1 + 20 = 22 variables were fitted with a linear model against the editing frequencies of half of 51,621 *Alu* editing sites with coverage ≥20× previously identified (Ramaswami et al., 2012). (J) Observed editing frequencies versus editabilities predicted from DNA for validation sites.

We challenged this A-to-I editing model by inducing *ADAR* expression in four breast cell lines (three tumor and one normal tissue derived cell lines) with interferon α, a known ADAR inducer (Patterson and Samuel, 1995). The effect of inducing *ADAR* overexpression on the editing frequency of *AZIN1* and four of the most edited *Alu* regions in the discovery series was analyzed by amplicon sequencing (Roche FLX sequencer). These experiments demonstrated: First, that the same sites were edited in all cell lines (Figure S3; Table S6), including 90 of the 91 sites detected by whole-transcriptome sequencing in vivo. Second, that the editing frequency profiles were similar across all cell lines (Figure S3). Third, that *ADAR* induction increased editing frequencies at all edited positions (Figures 4A and S3). Fourth, that *ADAR* induction and/or increase of depth of coverage increased the number of detected editing sites (Figure 4B). Due to deeper coverage (typically >1,000× for the Roche FLX sequencer) of the cell line amplicons, we identified 137 new sites in addition to the 90 in the discovery data set, which suggests there are likely more sites to identify in breast tissue.

We took advantage of the long reads (>300 bp) and high coverage of the Roche FLX data to further validate our model by applying it to thousands of individual mRNA molecules transcribed from the same DNA region in the same individual. Focusing on one 256-bp *Alu* region in one cell line, 65 of 68 adenosines potentially targeted by ADAR (Figure 4C) were edited in at least one of the 2,842 mRNA molecules analyzed. The number of edited positions per transcript was highly variable, ranging from 0 to 26 (38% of all adenosines). As expected, the sets of edited positions in “low-edited” mRNA molecules tended to be subsets of those edited in “high-edited” mRNA molecules. These findings further validate our A-to-I editing model. Nevertheless, the editing process had a strong stochastic component at the level of individual molecules. This is at odds with the deterministic nature of editability, a quantity defined at the level of populations of RNA transcripts. We propose to reconcile these two viewpoints by interpreting editability as a probability of edition by ADAR.

### Quantitative Estimation of Site-Specific Editability with the Logistic Model

The dependence of site-specific editing frequencies on ADAR protein expression in our in vitro experiments is shown in Figure 4D. Editing frequencies increase monotonously with ADAR until a site-specific saturation threshold is reached. This suggests that these frequencies could be approximated with the logistic model, *f*(*x*) = *ε*_*i*_/(1+exp(*ω*_*i*_*−x*)), at each site *i*. The offset of the s-shaped curve is controlled by *ω*_*i*_ and the editing frequency at saturation by *ε*_*i*_. We propose *ε*_*i*_—a quantity independent of ADAR expression—as the mathematical definition of site-specific editability, putting this concept on a firm quantitative ground.

We estimated *ε*_*i*_ and *ω*_*i*_ by fitting the logistic model to each one of the dose-response curves shown in the above graphics. A typical fit is shown in Figure 4E (see also Figure S4) and the distributions of *ε*_*i*_ and *ω*_*i*_ across all sites in Figures 4F and 4G. As expected, *ε*_*i*_ estimates are spread over the entire [0, 1] interval. The *ω*_*i*_ estimates are centered around a unique value, i.e., *ω*_*i*_ is essentially site independent. Related p values (Figure S4) are small considering that only four points were available for each fit. Although saturation was reached for two ADAR expression data points in one experiment but not in the others, the estimates obtained for independent experiments were consistent (ρ = 0.97, p < 2 × 10^−16^; Figure S4). The lower coverage of our in vivo data was not sufficient to adequately fit the logistic model, but *ε*_*i*_ estimated in vitro is highly correlated with the mean editing frequency measured in vivo (Figure 4H). In vivo editing is, on average, well below saturation (Figure 4H). Hence, the logistic model provides an operational procedure to derive useful quantitative estimates of site-specific editability from dose-response data.

### Site-Specific Editability Is Correlated with Local Sequence Features

We hinted that editability depends upon biophysical interactions between an individual site with its surrounding RNA sequence and partnering as a duplex with *ADAR*. This implies that editability should be partially predictable from the sequence data, so we sought to develop and validate a simple proof-of-principle DNA-based statistical model for editability. The model relies on the notions that (1) an edited site must be part of an RNA duplex, implying that it lies within a sequence with a nearby palindromic match, and (2) ADAR activity depends upon a specific nucleotide sequence in the vicinity of the edited base (Figure 4I; Supplemental Experimental Procedures). To build the model, we analyzed the editing frequencies of 51,621 edited *Alu* sites with ≥20× coverage from an independent sample sequenced at very high coverage (Ramaswami et al., 2012). These sites were then ordered by genomic position. The first half was used to fit a statistical model of the edit frequency based on DNA data alone. Editability scores were then computed for the second half of the sites (not used to train the model), which turned out to be strongly associated with the observed editing frequencies (Figure 4J). Our validated statistical model supports the notion that the editability of a given site is partly determined by the local site-specific DNA features. Of note, the logistic fit of dose-response data, not the DNA-based model, should be used to estimate quantitatively editability.

### Association of ADAR Expression, A-to-I Editing, and Clinico-Pathological Variables

The relevance of ADAR expression to the A-to-I editing process led us to analyze its tissue and cellular localization by immunohistochemistry (IHC). Uniform ADAR expression was detected in cancer cells (Figures 2D–2F) but to a lesser extent in normal cells and tumor-infiltrating lymphocytes (TILs; see Figure 2E). Moreover, ADAR staining was markedly stronger in nucleoli (Figure 2F), in agreement with previous findings (Desterro et al., 2003, Sansam et al., 2003).

To investigate the potential clinical impact of A-to-I editing, we determined whether the mean editing frequency was associated with the tumor cell content (i.e., the proportion of malignant epithelial cells, adipose, stroma, normal epithelial cells and TILs) and/or well-established clinico-pathological parameters, including estrogen receptor, progesterone receptor, the proliferation marker Ki67, HER2 status, tumor size, nodal status, and histological grade. The mean editing frequency was positively correlated with the percentage of TILs (Spearman’s correlation ρ = 0.3, p = 0.02), tumor size (ρ = 0.3, p = 0.01), and HER2 IHC staining (ρ = 0.3, p = 0.01; Figure S5; Tables S1 and S3). Multivariate analysis of this data set suggests that TILs and HER2 IHC are dependent variables in their association with editing frequency (Figure S5).

To circumvent our limited sample size, correlations between these variables and ADAR expression were assessed in a large cohort of 787 BC patients with HER2 analyzed by IHC (Curtis et al., 2012). TILs were not scored in this series so the level of Signal Transducer and Activator of Transcription 1 (*STAT1*) expression, a proxy for type I interferon response, was used instead. This independent BC series confirmed an association between ADAR and STAT1 expression but not for HER2 status or tumor size (Figure S5). The lack of an association with estrogen receptor, Ki67, and HER2 indicates that ADAR expression is not correlated with a specific BC subtype beyond their link with the adaptive immune response.

### The Interferon Response and Gains in *ADAR* Copy Number Independently Control A-to-I Editing in Cancer

**Figure 5:**
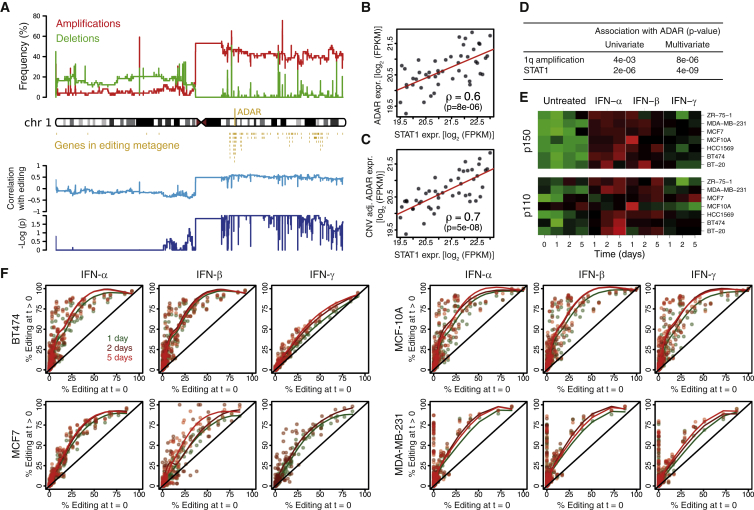
*ADAR* Amplification and the Interferon Response Are Independent Predictors of ADAR Expression in Cancer (A) The top panel shows the frequencies of amplifications/deletions along chromosome 1 in our series. The middle panel shows the genes whose expression is highly associated with that of ADAR. Nineteen genes not located on chr1 are omitted. The bottom panel shows the Spearman’s correlation coefficient and associated p values of non-segmented copy-number array probes with the sample-wise mean editing frequencies. (B) Dots represent tumor samples, with STAT1 expression on the x axis and ADAR expression on the y axis. (C) Same as (B) with *ADAR* expression adjusted for *ADAR* copy number. (D) Association p values of *ADAR* copy number and STAT1 expression with ADAR expression increase in a multivariate analysis, demonstrating that ADAR expression is independently associated with these two variables. (E) Seven breast cancer cell lines were exposed to interferon α, β, and γ for 1, 2, and 5 days. Western blots quantifications are depicted for each cell line, interferon, and time. Because expression dynamic ranges vary among cell lines, each line has its own color scale extending from low expression in green to high expression in red. The underlying gels are presented in Figure S7 and blot quantification in Table S6. Corresponding mRNA RT-PCR expression data are shown Figure S7 and detailed Table S7. (F) Editing frequencies in the absence of treatment (x axis) versus interferon treatment (y axis). Points depict the editing sites in AZIN1 and the four *Alu* regions of Figure S3. Points are above the identity line x = y (black diagonals); i.e., interferons increase editing frequencies at all sites. Library preparation failed for MCF7/IFN-γ at 5 days. Limited sequencing coverage precluded detection of some editing events for MDA-MB-231, t = 0 and t = 1 days.

The biological processes potentially associated with RNA editing were investigated by searching for genes whose expression had a strong positive correlation with the mean editing frequency (details in the Supplemental Experimental Procedures). Remarkably, 62 of the 85 genes identified were located on chromosome 1q (p = 10^−66^). Since *ADAR* is located on chromosome 1q, we next used SNP array data to determine *ADAR* copy numbers in our samples. *ADAR* amplification was frequent in our series (44%) and correlated with high mean editing frequencies (Figure 5A).

Chromosome 1q contains hundreds of genes and therefore its amplification could have a systemic impact on the BC transcriptome (Curtis et al., 2012). Therefore, we further characterized the genes correlated with editing that were independent from 1q amplification. First, the microarray expression data were adjusted for 1q copy number to remove any potential confounding effects of *ADAR* amplification, and then gene set analysis was performed (Efron and Tibshirani, 2007) to identify canonical pathways associated with the mean editing frequency. The 13 significant pathway gene sets revealed by this analysis were all involved in interferon responses, interferon-related DNA and RNA sensing, and lymphocyte biology (Figure S6). We also investigated gene sets with shared transcription factor binding motifs between their promoters. The seven significant gene sets identified were overwhelmingly related to *NFκB* and the interferon response, including the Interferon Response Factors *IRF1*, *IRF2*, and *IRF7* (Figure S6). To further investigate the relationship between interferon-related genes and ADAR expression, the median expression levels of STAT1 (Figure 5B) and 389 type I interferon-inducible genes (Figure S6) derived from ten microarray studies (Schoggins et al., 2011) were measured. The expression of STAT1 and the 389 genes were positively associated with ADAR expression, suggesting that increased editing was part of a broader type I interferon response related to the chronic inflammatory state in cancer.

The respective roles of *ADAR* copy number and STAT1 expression (as a proxy for interferon response) in the A-to-I editing process were further defined using multivariate analysis to demonstrate that they are independently associated with *ADAR* expression (Figures 5B–5D). STAT1 was correlated with ADAR expression (Figure 5B), and this correlation could be strengthened by adjusting ADAR expression for *ADAR* DNA copy number (Figures 5C and 5D). Taken together, STAT1 and *ADAR* copy number explained 53% of ADAR expression variation. The independent effect of type I interferon response and *ADAR* amplification was also supported by measuring the constitutive p110 and interferon-inducible p150 ADAR isoforms (Figure S6). STAT1 expression was more strongly correlated with p150 than p110, and, conversely, *ADAR* copy number was more strongly correlated with p110 than p150.

While *ADAR* amplification is likely limited to malignant epithelial cells, the type-I interferon effect could be principally mediated by TILs. To further explore this, we treated seven breast cell lines (derived from the four principal BC molecular subtypes and normal breast) with individual interferons (α, β, and γ) to determine whether editing can be directly increased by interferon. ADAR p150 protein expression increased with all three interferons in all cell lines at each time point (Figures 5E and S7), while p110 induction was weaker and less consistent. The moderate but significant correlation between p110 and STAT1 mRNA detected in primary tumors suggests that a small amount of p110 was induced (Figure S6). The same four cell lines used to validate our A-to-I editing model were analyzed for p150 and p110 *ADAR* mRNA isoform expression levels, the editing proportion of *AZIN1* and the four most edited *Alu* regions previously selected. The mRNA levels for p110 and p150 isoforms paralleled their protein expression (Figure S7). Moreover, editing increased at all editable sites with all interferons in the four cell lines (Figure 5F). Higher editing levels were observed at 2 or 5 days compared to untreated or 1 day. The induction of ADAR and editing was lowest for IFN-γ. These experiments confirm that type-I interferon response affect A-to-I mRNA editing in epithelial cells.

### ADAR Is Involved in Cell Proliferation and Apoptosis in Breast Cancer

**Figure 6:**
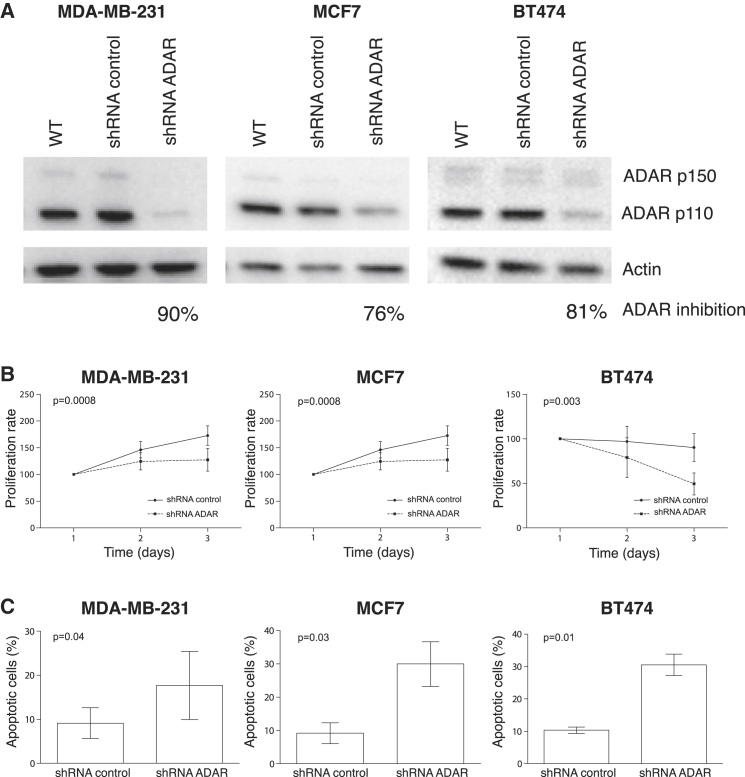
ADAR Involvement in Cell Proliferation and Apoptosis (A) Western blot analysis of ADAR silencing after shRNA lentiviral transduction in MDA-MB-231, MCF7, and BT474 breast cancer cell lines. (B) ADAR silencing statistically decreases cell proliferation. Cell growth curves for ADAR-knockdown cells (shRNA ADAR) and control cells (shRNA control) in MDA-MB-231, MCF7, and BT474 BC cell lines. (C) ADAR silencing statistically increases cell apoptosis. Illustration of the percentage of apoptotic cells in ADAR-knockdown cells (shRNA ADAR) and control cells (shRNA control) in MDA-MB-231, MCF7, and BT474 BC cell lines. Error bars depict SDs of three independent experiments.

Given that we have shown that both ADAR expression and mean editing frequency were higher in breast tumors compared to matched normal tissues, we aimed to further investigate ADAR’s role on cell proliferation, migration, and apoptosis. To that purpose, ADAR expression was stably knocked down in three representative BC cell lines (MDA-MB-231, MCF7, and BT474) using small hairpin RNA (shRNA) lentiviral particles (shRNA ADAR). The three cell lines were also transduced with scramble shRNA lentiviral particles (shRNA control) as a negative control for the functional experiments. ADAR silencing was confirmed by western blot analysis (Figure 6A).

To assess the role of ADAR in cell proliferation, MTT assays were performed. These experiments showed that ADAR silencing led to a statistically significant decrease in cell proliferation (shRNA ADAR) compared to the control cells (shRNA control) in all cell lines (Figure 6B). These results suggest that ADAR promotes cell proliferation. No significant effect of ADAR silencing was found on cell migration. The role of ADAR in apoptosis was investigated using Annexin V assays. ADAR silencing led to a statistically significant increase in cell apoptosis (shRNA ADAR) compared to the control cells (shRNA control) in all cell lines (Figure 6C) suggesting that ADAR may act as an anti-apoptotic factor.

### The Role of *ADAR* Copy-Number Gains and Interferon Responses in Other Cancers

**Figure 7:**
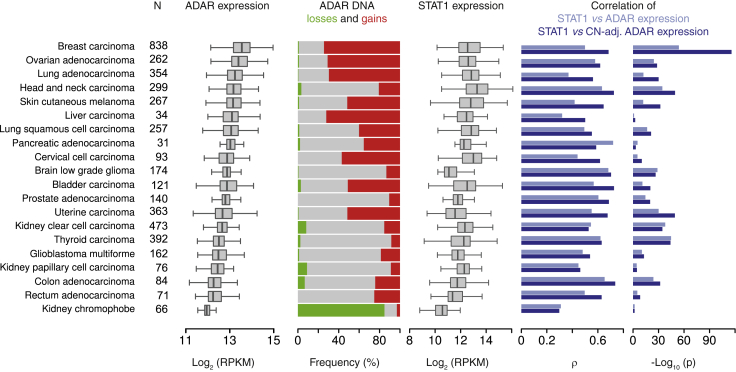
*ADAR* Amplification and the Interferon Response Predict ADAR Expression in Human Cancers We included all TCGA data sets and tumors (see “N” column) for which both copy-number and RNA-seq expression data (pipeline v.3) were available. Data sets are ordered by decreasing median ADAR expression (top to bottom). The three leftmost plots depict the distributions of ADAR expression, *ADAR* DNA copy number, and STAT1 expression across each data set. The two rightmost bar plots extend to TCGA data the calculation presented for our data in Figures 5B and 5C. In most cancers, adjusting ADAR expression for *ADAR* copy number increases the Spearman correlation, ρ, with STAT1 (cf. the dark blue bars to the light blue bars).

*ADAR* amplification is frequent in human cancers (Figure 7) and inflammatory responses are pervasive in this disease. This information led us to investigate whether these two factors were related to ADAR expression in 4,480 cancers from The Cancer Genome Atlas (TCGA, http://cancergenome.nih.gov/) for which sample-matched expression and copy-number profiles were available. The representative analyses shown in Figures 5B and 5C were reproducible across the TCGA data set, which spanned 20 types of cancer from 16 organs (Figure 7). Overall, ADAR expression was consistently associated with both *ADAR* copy number and STAT1 expression. Similar to BC, adjusting ADAR expression for *ADAR* copy number increased the correlation between ADAR and STAT1 for all except pancreatic, kidney, and thyroid tumors. The frequency of *ADAR* amplification was low in kidney and thyroid tumors, therefore correcting for *ADAR* copy number had a limited effect. These data suggest that ADAR expression could be principally driven by interferon in these two types of cancer. In most cancers, however, the editing process is driven by both type I interferon and *ADAR* copy-number amplification. A correlation between *ADAR* copy number and ADAR expression has also been recently reported in esophageal cancer (Qin et al., 2014).

## Discussion

The magnitude of A-to-I editing in cancer as well as the mechanisms controlling and regulating the A-to-I editing machinery are currently unknown. To address both points, we performed a survey on RNA editing in cancer by profiling dozens of BCs and matched healthy breast tissues. The sample size of this study opened a window on principles governing A-to-I editing that were previously out of reach. A significant finding from our study was the demonstration that the same sites are edited in normal and tumor breast tissues as well as in several BC cell lines. We further showed that while the editing frequency profiles are correlated across tissues and BC cell lines, the frequency of editing is significantly higher in tumors compared to their matched normal breast tissues. High editing frequencies are detected in samples with high ADAR expression. These data provide the basis for our A-to-I editing model, where increases in ADAR expression increase the editing frequency of all editable positions in the transcriptome. We successfully validated this model in BC cell lines and showed that ADAR control of site-specific editing frequency can be approximated with the logistic model. ADAR’s site-specific activity, that we call editability, is partly influenced by the biophysics of interactions between nucleotides in the surrounding RNA sequences and their duplex partnering with ADAR and can be estimated from dose-response experiments. Finally, we showed that ADAR expression is controlled by 1q amplification and inflammation in human cancers.

In our study, longer *ADAR* induction times and/or deeper sequencing coverage increased the number of editing sites detected. Interestingly, no plateau was reached at the depths we investigated, with up to 3 × 10^7^ aligned reads per sample. A previous study made a similar observation using a coverage of up to 5 × 10^8^ mRNA reads/sample, where no plateau was reached despite >140,000 A-to-I sites detected in the *Alu*’s (Ramaswami et al., 2012). A hundred million sites could be edited in humans (Bazak et al., 2014b). Differences in number of edited sites between the cited works and the present study could be due to the cell type analyzed (e.g., lymphoblastoid cell line versus breast tissues/cell lines) and the DNA (e.g., whole-genome versus coding sequences and their neighborhood) and/or RNA sequencing strategies (Table S4). For example, several studies used the GM12878 and the YH (also known as SRA043767) cell lines for which the transcriptomes were sequenced at the outstanding depth of 0.5–1.2 billions reads and compared to the matched whole-genome sequence. In these studies, the number of editing sites, ranging from ∼20,000 to ∼2 million, is commensurate to the number of callable bases. Conversely, the studies with lower individual transcriptome coverages report less editing sites, like ours (560 sites) and Bahn et al. (2012) (5,965 sites). Bahn et al. had access to the full genome sequence, while we had access only to the coding DNA sequence (CDS) regions and their neighborhood. In addition, the detection pipeline specificity versus sensitivity trade-off may also play a role. Most previous studies used the A > G ratio as a surrogate for error rates; i.e., they assumed that A-to-I is the only significant RNA editing type and that all A > G RDDs are bona fide editing events. The A > G rate in *Alu* regions is 80% in Bazak et al., 2014a, Bazak et al., 2014b), 90% in Peng et al. (2012), 96% in Ramaswami et al. (2012), and 100% in our study. Our pipeline is therefore more conservative according to this criterion, and consequently less putative editing sites were detected. It is anticipated that a large number of additional A-to-I editing sites beyond those identified here remain to be discovered in BC. The data presented here clearly demonstrate that A-to-I editing is a pervasive phenomenon in cancer and suggest that it is a major source of mRNA sequence variability in breast and potentially other types of cancer (Paz-Yaacov et al., 2015, Han et al., 2015). Editing has the potential to significantly impact transcriptional regulation and cellular functions in tumor cells. Indeed, our in vitro studies have shown that *ADAR* silencing decreases cell proliferation and promotes apoptosis supporting the potential carcinogenic role of ADAR and consequently A-to-I editing in BC.

Multiple studies are revealing that aberrant expression of ADAR and APOBEC families of enzymes occurs in many human diseases, including cancer. Since the first studies implementing the sequencing technology in humans, ADAR appeared to be one of the highest overexpressed genes in BC, and its recoding potential started to emerge (Shah et al., 2009). More recent works have shown that in breast and other tumor types mutational signatures are associated with APOBEC family proteins (Alexandrov et al., 2013, Nik-Zainal et al., 2012) with evidence that APOBEC-mediated mutagenesis is highly active in human cancers (Burns et al., 2013, Roberts et al., 2013, Swanton et al., 2015, Zhang et al., 2015). Although the relevance of ADARs and RNA editing in cancer just begins to be recognized (Avesson and Barry, 2014, Han et al., 2014, Mo et al., 2014, Salameh et al., 2015, Witkin et al., 2015), the link between A-to-I editing by ADAR and the type I interferon response shown in our study suggests that the cancer immune response can influence ADAR’s activity, as shown in other systems. A significant role for ADAR is further supported by our demonstration that its expression is significantly upregulated by *ADAR* copy-number gains in breast (up to 75%) and other cancers (up to 70%). Overall, these data highlight the potential magnitude of A-to-I RNA editing in tumors and thereby the possibility for large-scale clinical implications. RNA editing and/or APOBEC-mediated mutagenesis could shape the immunogenicity of the tumor and thereby directly affect anti- and/or pro-tumor immune responses. RNA editing itself, the processes it regulates and its potential to differentially direct activities in response to the chronic inflammatory tumor microenvironment, may have important implications for clinical progression in breast and other cancers.

The widespread editing we observed, in combination with the conservation of editing sites detected across tissues and patients, suggests there might be clinical and therapeutic implications for a wide range of cancer patients. However, modulation of editing at an individual site is entangled with many processes. The model we established for A-to-I editing implies that modulation of ADAR will also affect all editable sites in expressed transcripts. In addition, ADAR has been shown to influence miRNA processing (Heale et al., 2009, Ota et al., 2013, Shoshan et al., 2015, Tomaselli et al., 2013, Yang et al., 2006), to control mRNA transcript stability (Wang et al., 2013) and to affect several RNA processing pathways (Bahn et al., 2015). Finally, variation of ADAR expression in vivo will possibly be associated with modification of the hundreds of genes located on 1q and/or controlled by interferon. Determining whether increasing A-to-I editing limits or enhances cancer progression will need to take into account all of these potential variables. More research is needed to identify the critical editing sites, establish their potential as markers of cancer evolution, and investigate them as a new class of therapeutic targets.

## Experimental Procedures

The study has been approved by the Institut Jules Bordet Ethics Committee (approval number: CE1967). The methods are fully detailed in the Supplemental Experimental Procedures. In brief, the exome and transcriptome of 58 well-characterized BC samples representing the four main known subtypes based on immunohistochemistry, namely, TN, HER2^+^, luminal A, and luminal B, and ten matched normal samples were profiled using exome sequencing and RNA-seq in paired-end mode on the Illumina HiSeq 2000 platform. Gene expression and SNPs profiles were obtained with Affymetrix HG-U133 Plus 2.0 Array chips and Affymetrix Genome-Wide Human SNP Arrays 6.0 for 57 and 49 tumor samples, respectively. RNA reads obtained from RNA-seq were aligned simultaneously on the human genome and all known exonic junctions. Variant calls were submitted to a series of filters limiting artifact associated with RNA-seq. The identified RNA-DNA differences (RDDs) were validated in an independent cohort of 15 BC samples; moreover, few events as well as their editing frequencies were validated using an independent technology (Roche FLX sequencer). The effect of interferon (IFN) on ADAR expression and editing was evaluated on six BC cell lines and one immortalized, non-transformed mammary epithelial cell line, MCF-10A. Cell lines were treated for 1, 2, or 5 days with IFN α, β, or γ. The effect of treatment on ADAR p110 and p150 protein and gene expression levels were evaluated quantifying the immunoblot signals and qRT-PCR data, respectively, while the effect of IFN treatment on editing distribution and frequency was investigated using amplicon sequencing (Roche FLX sequencer). In each sample, the mean editing frequency was correlated with clinico-pathological parameters and the expression of ADAR. The intracellular localization of ADAR was defined using immunohistochemistry. The association between editing and *ADAR* amplification and/or a surrogate of interferon response (STAT1 expression) was evaluated in breast and 19 additional cancer types obtained from TCGA. Finally, the effect of ADAR knockdown on cell proliferation, migration, and apoptosis was evaluated in three representative BC cell lines transduced with shRNA lentiviral particles.

## Author Contributions

C.S., V.D., and M.A. made substantial contributions to study conception and design. D.F., F.R., and N.K. acquired the data (acquired and managed patients/samples, performed cell lines experiments, etc.). A.L., F.L., and P.J.C. generated the sequencing data. R.S. and D.L. performed the pathology evaluation. K.P. generated the breast cancer organoids. D.G., V.D., D.B., T.K., A.S., C.S., D.F., and F.R. analyzed and interpreted the data (e.g., statistical analysis, biostatistics, computational analysis). V.D., D.F., C.S., C.D., D.G., K.W.-G., F.R., D.B., M.P., and R.S. made substantial contributions to writing, review, and/or revision of the manuscript. V.D. and C.S. supervised the study.

## Accession Numbers

Sequencing and SNPs array data obtained from the enrolled patients are archived at European Genome-phenome Archive, https://www.ebi.ac.uk/ega, under accession number EGAS00001000495; amplicon sequencing data obtained from cell lines are archived at European Nucleotide Archive, http://www.ebi.ac.uk/ena, under study accession number ERP004253; gene expression array data are archived at the NCBI GEO, http://www.ncbi.nlm.nih.gov/geo, under accession number GSE43358. Clinical information, results of sequence and arrays preprocessing, and biological assays are available in Tables S1, S2, S3, S4, S5, S6, and S7.

## Supplemental Information

Document S1. Supplemental Experimental Procedures and Figures S1–S7

Table S1. Patient Data, Related to Figures 1, 2, 3, 4, and 5

Table S2. Putative RNA-DNA Differences in In Vivo Samples, Related to Figures 1, 2, 3, 4, and 5

Table S3. Sample Data, Related to Figures 1, 2, 3, 4, and 5

Table S4. Comparison of A-to-I RNA Editing Studies and Detection Pipelines, Related to Figure 1

Table S5. AZIN1 Editing Measured by Amplicon Sequencing in 30 Patient-Matched Tumor/Normal Pairs, Related to Figure 2

Table S6. ADAR Protein Expression Quantification and Editing Frequency in In Vitro IFN Experiments, Related to Figures 4 and 5

Table S7. ADAR RT-PCR mRNA Expression in In Vitro IFN Experiments, Related to Figures 4 and 5

Document S2. Article plus Supplemental Information
