# Supplementary material for: Principles Governing A-to-I RNA Editing in the Breast Cancer Transcriptome
Source: Cell Rep. 2015 Oct 1;13(2):277–89. doi: 10.1016/j.celrep.2015.09.032 (PMC5326813; doi:10.1016/j.celrep.2015.09.032)
Supplement: Document S1. Supplemental Experimental Procedures and Figures S1–S7 [file mmc1.pdf]

Cell Reports

Supplemental Information

Principles Governing A-to-I RNA Editing in the Breast Cancer Transcriptome

Debora Fumagalli, David Gacquer, Françoise Rothé, Anne Lefort, Frederick Libert, David Brown, Naima Kheddoumi, Adam Shlien, Tomasz Konopka, Roberto Salgado, Denis Larsimont, Kornelia Polyak, Karen Willard-Gallo, Christine Desmedt, Martine Piccart, Marc Abramowicz, Peter J. Campbell, Christos Sotiriou, and Vincent Detours

Supplemental Information

Supplemental Figures 2
Supplemental Tables 9
Supplemental Experimental Procedures 10
Patients and sample characterization and preparation 10
Detection of RNA-DNA differences 11
Protein expression, mRNA expression and DNA copy number profiling 15
Cell lines experiments 16
Statistical Analysis 19
Supplemental References 22

Supplemental Figures

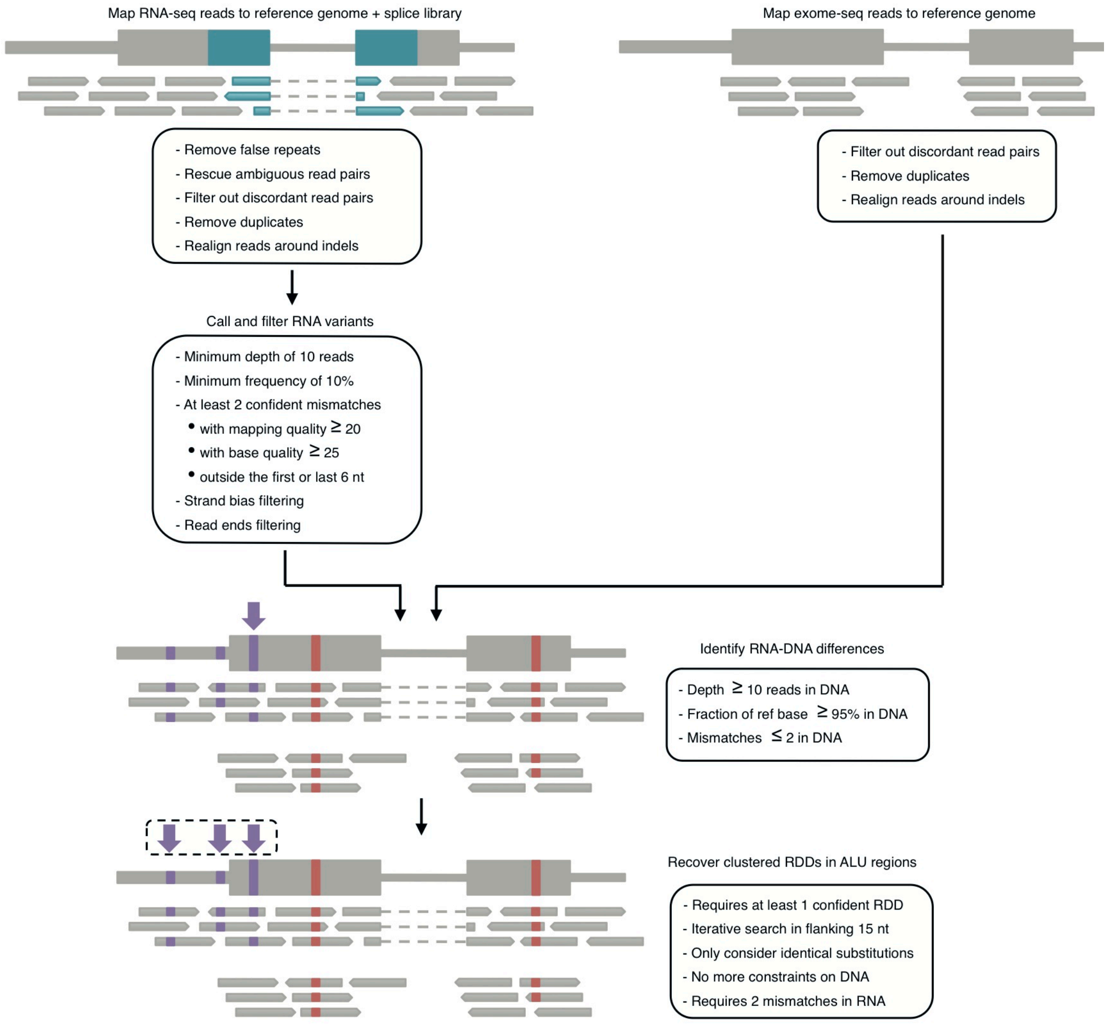

Figure S1. Overview of the pipeline for RNA/DNA differences (RDDs) detection (Related to Figures 1-5). Details are presented in the Supplemental Experimental Procedures.

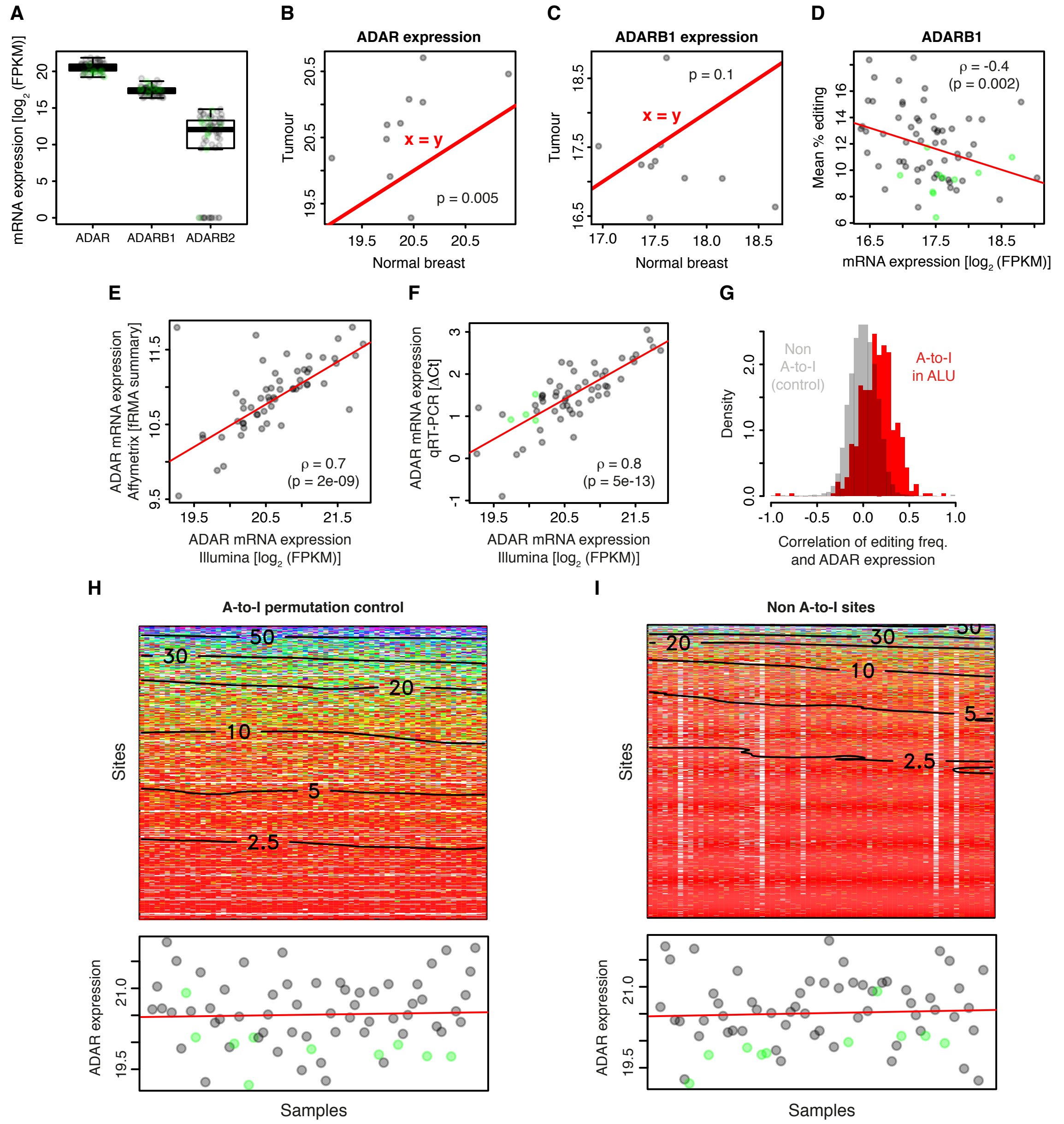

Figure S2. Expression and correlation with A-to-I RNA editing for the *ADAR* isoforms (A-D, Related to Figures 2 and 3) and (E-I) additional controls associated with Figure 3. (A), Expression of *ADAR*, *ADARB1* and *ADARB2* across our cohort. **(B)**, Each point represents a sample with the expression of *ADAR* in the normal breast tissue depicted on the x-axis and the expression in the matched tumor breast tissue on the y-axis. All but one point are above the x=y identity line, demonstrating that *ADAR* expression is higher in tumors than in normal tissues. The p-value was calculated from a Wilcoxon paired signed test. **(C)**, Same as **(B)** for *ADARB1*. **(D)**, each point represents a sample with *ADARB1* expression on the x-axis and the mean editing frequency on the y-axis. *ADAR* expression quantification from whole transcriptome sequencing is highly consistent with, (**E**), Affymetrix microarray and, (**F**), qRT-PCR quantifications. (**G**), Distribution of Spearman's correlations across the samples of the RNA-seq expression of *ADAR* and the editing frequencies of individual sites: 560 Alu A-to-I sites (red), and as negative control 11,312 putative non A-to-I RDDs (grey). (**H** and **I**), Heatmaps of editing frequencies after random permutation editing of frequencies across samples, depicted in panel (**H**), and of non-A-to-I putative RDDs, depicted in panel (**I**). The gradient in these negative control heatmaps is from top-to-bottom, without left-to-right component. Green dots represent tumor-matched normal samples.

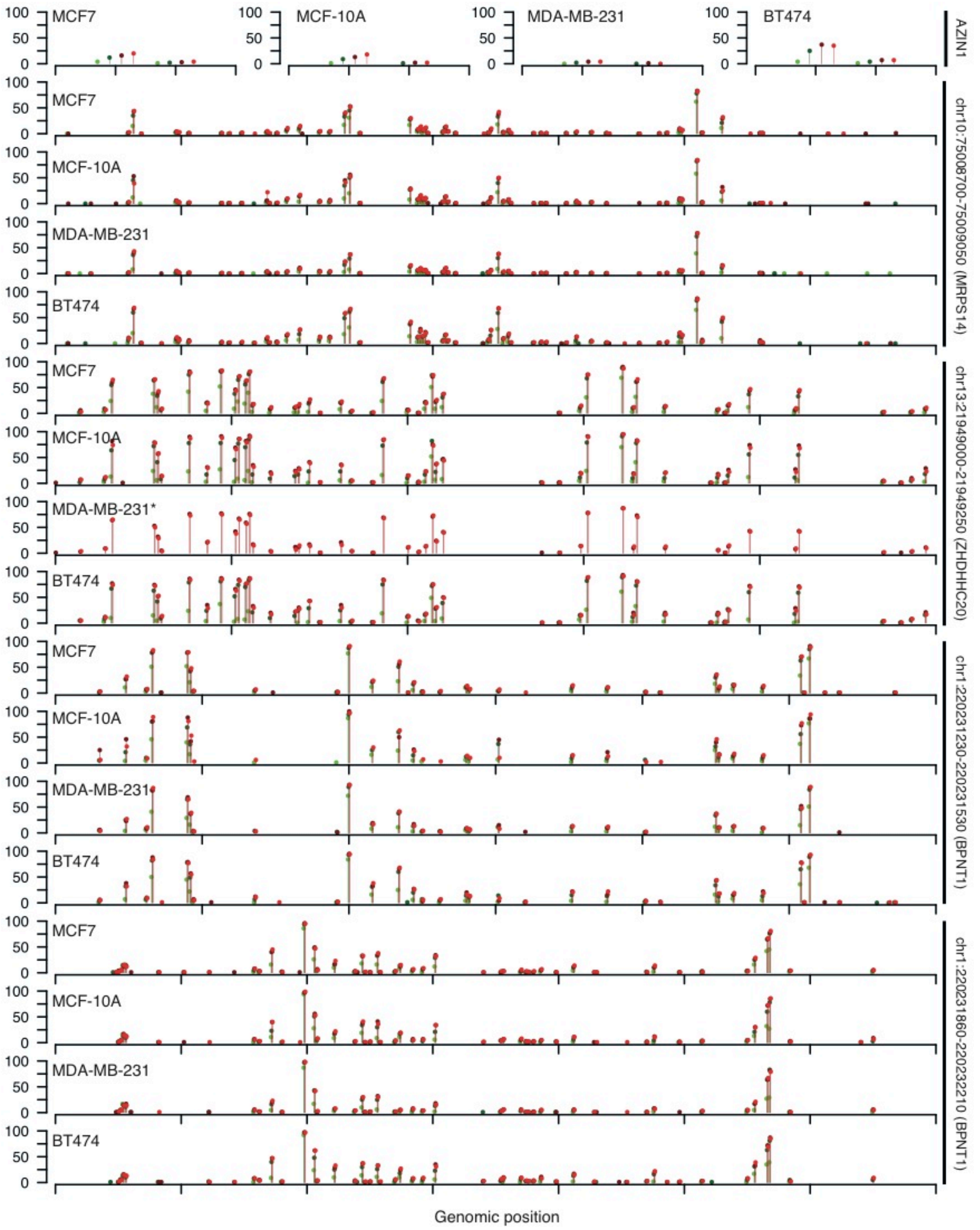

Figure S3. The same sites are edited in four breast cell lines (three tumor and one normal tissue derived cell lines) and increasing *ADAR* expression increases the editing frequency at all these sites (Related to Figure 4A). Editing in *AZIN1* and 4 *Alu* regions is shown in 4 breast cell lines and 4 ADAR protein expression levels (same color scale as in Figure 4A). The x-axis scales are different for each region (see right-side labels). (*) Library preparation failed for the two lower ADAR expression samples for MDA-MB-231, chr13:21949000-21949250.

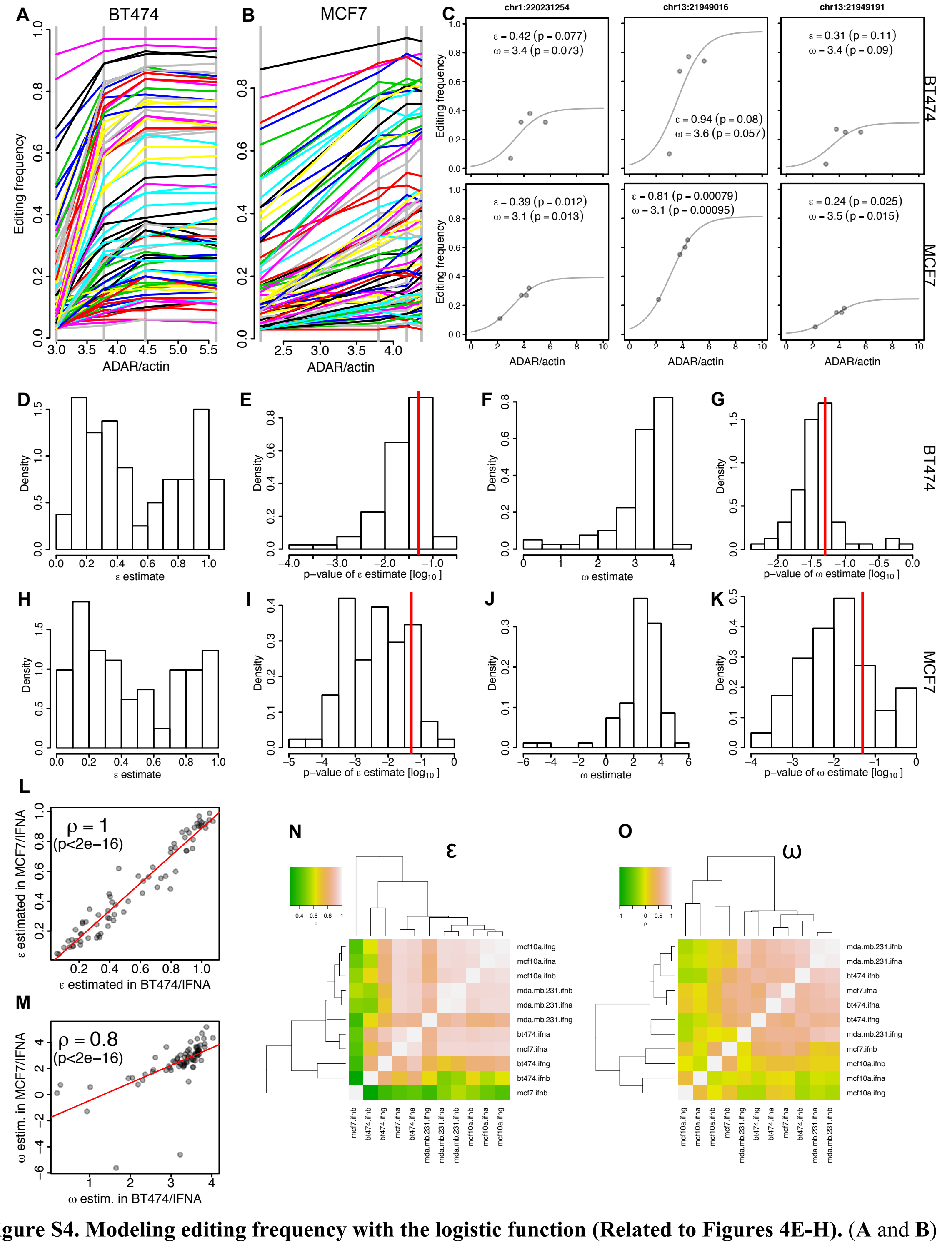

Figure S4. Modeling editing frequency with the logistic function (Related to Figures 4E-H). (**A** and **B**) Dose-response curves for cell lines BT474 and MCF7 (**A** also shown in main text). ADAR was induced via interferon treatment. Note that saturation is reached for the third and fourth points for BT474, but not MCF7. (**C**), Fits of the logistic model to dose-response data for three editing sites are shown. (**D-K**), Overview of all the logistic fits for dose-response curves shown in (**A**) and (**B**), with distribution of ε_i (**D**, **H**), ω_i (**F**, **J**) and associated p-values (**E**, **I**, **G** and **K**; Red lines denote the p=0.05 limit). ε_i but not ω_i estimates are distributed around a central value, suggesting that ε_i, but not ω_i, is site-specific. (**L** and **M**), comparison of the logistic parameters estimated from the BT474 and MCF7 experiments. (**N** and **O**), Correlations of ε_i, and ω_i, between all pairs of interferon treatment experiments performed in this study for which enough data was available. Lower correlations typically resulted from aberrant fits caused by data scarcity, the fits rest on 4 data points, and suboptimal doses.

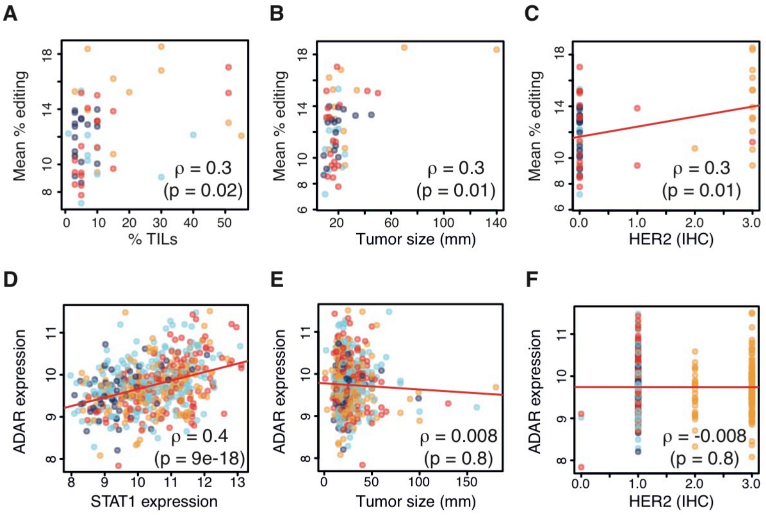

G

	Univariate	Multivariate p-values			
		TILs and HER2	TILs and T size	HER2 and T size	All
% TILs	0.01	0.07	0.02		0.06
Tumor size	0.0001		0.0002	0.0009	0.0009
HER2 (IHC)	0.004	0.02		0.04	0.13

Figure S5. Correlations of A-to-I editing and breast cancer clinico-pathological variables in our cohort (Related to Figure 2). The mean editing frequency is significantly correlated with (**A**), the proportion of tumor-infiltrating lymphocytes (TILs), (**B**), tumor size and (**C**), HER2 defined by immuno-histochemistry. Point colors depict subtypes: navy blue, luminal A; sky blue, luminal B; orange, HER2; red, triple negative. These associations were tested in 787 patients of the Metabric cohort (Curtis et al., 2012) for whom HER2 IHC was available. *ADAR* expression (a surrogate for the editing frequency, Figure 3A) and *STAT1* expression (a surrogate for TILs) were used in this analysis. An association was found with *STAT1* (**D**), but not tumor size (**E**) and HER2 IHC (**F**). (**G**), A multivariate analysis demonstrates that the associations of editing with HER2 and TILs are statistically related. Multivariate analysis decreases the significance of all variables when all three are combined together. We conducted bi-variate analyses to dispel the ambiguity of variables' dependencies. HER2 status and TILs were less significant when analyzed together than with tumor size. Thus, the dependency is mostly between TILs and HER2 status. Importantly, this figure depicts only significant associations. No significant correlations could be found between mean editing frequency and adipose content, stromal content, grade, nodal status, and ER, PGR and Ki67 immuno-histochemistry staining.

A

gene set	GSA score	p-value	FDR
KEGG_RIG_I_LIKE_RECEPTOR_SIGNALING_PATHWAY	0.3981	0	0
KEGG_CYTOSOLIC_DNA_SENSING_PATHWAY	0.6729	0	0
ST_TUMOR_NECROSIS_FACTOR_PATHWAY	0.6677	0	0
SIG_PIP3_SIGNALING_IN_B_LYMPHOCYTES	0.6815	0	0
PID_TXA2PATHWAY	0.6138	0	0
REACTOME_IMMUNOREGULATORY_INTERACTIONS_BETWEEN_A_LYMPHOID_AND_A_NON_LYMPHOID_CELL	1.2665	0	0
REACTOME_INTERFERON_ALPHA_BETA_SIGNALING	1.8057	0	0
REACTOME_INTERFERON_SIGNALING	0.8589	0	0
REACTOME_RIG_I_MDA5_MEDIATED_INDUCTION_OF_IFN_ALPHA_BETA_PATHWAYS	0.5511	0	0
REACTOME_CYTOKINE_SIGNALING_IN_IMMUNE_SYSTEM	0.5604	0	0
REACTOME_INFLAMMASOMES	0.869	0	0

B

gene set	GSA score	p-value	FDR
V$NFKAPPAB_01	0.2987	0	0
V$IRF1_01	0.4256	0	0
V$IRF2_01	0.5835	0	0
V$ISRE_01	0.5547	0	0
V$IRF7_01	0.5173	0	0
V$ICSBP_Q6	0.4919	0	0
STTTCRNTTT_V$IRF_Q6	0.8069	0	0

C

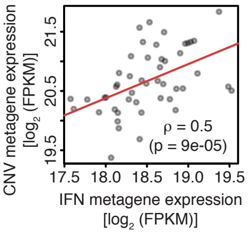

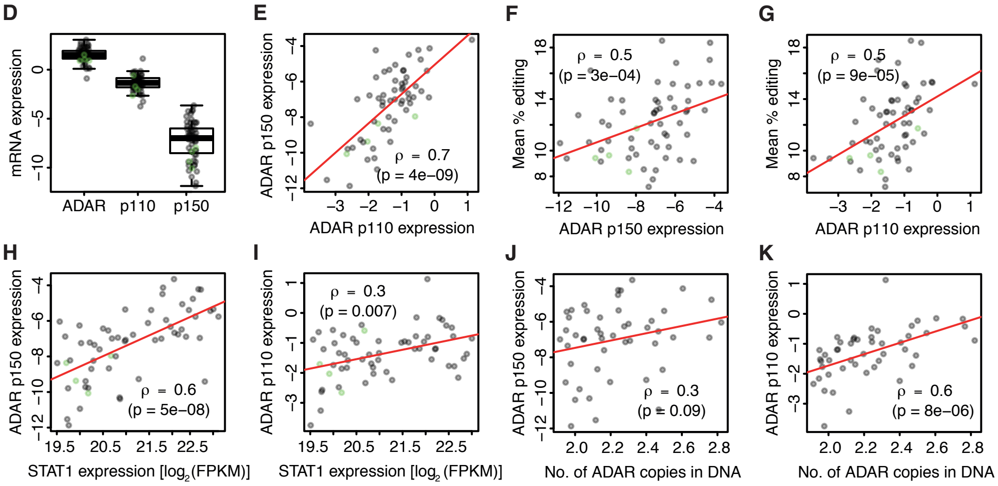

Figure S6. Gene set and metagenes analysis of the correlation of A-to-I editing with gene expression (A-C), and expressions of *ADAR* isoforms p110 and p150 support a control of *ADAR* expression by 1q amplification and interferon (D-K) (Related to Figure 5). Affymetrix expression data were adjusted for *ADAR* DNA copy number and then screened for gene sets associated with the mean editing frequency. (**A**), Screen of gene sets defining canonical pathways. (**B**), Screen of genes set defined by genes sharing binding motifs for the same transcription factor in their promoter. Null p-values and false discovery rate (FDR) means that no random gene sets in 500 had a higher GSA score (see Supplemental Experimental Procedures). (**C**), Correlation between DNA copy number-adjusted *ADAR* expression and the median expression of 389 interferon-induced genes compiled from 10 studies. The relative expression of *ADAR,* its constitutively active form, p110, and the interferon-inducible form, p150, were measured by qRT-PCR for 58 samples (see Table S3), depicted as individual data points in the panels. (**D**), The truncated form of *ADAR*, p110, predominates over the full-length transcript, p150. (**E**), The expressions of the two isoforms are highly correlated and, (**F** and **G**), are correlated with the mean editing frequency. *STAT1* is correlated with the expression of interferon-inducible p150 (**H**), but less with the expression of p110 (**I**). Conversely, the correlation with *ADAR* copy number is lower for p150 (**J**) than p110 (**K**) — in agreement with the notion that the association of p150 with *ADAR* copy number is confounded by its strong dependence on interferon control. Green dots represent tumor-matched normal samples.

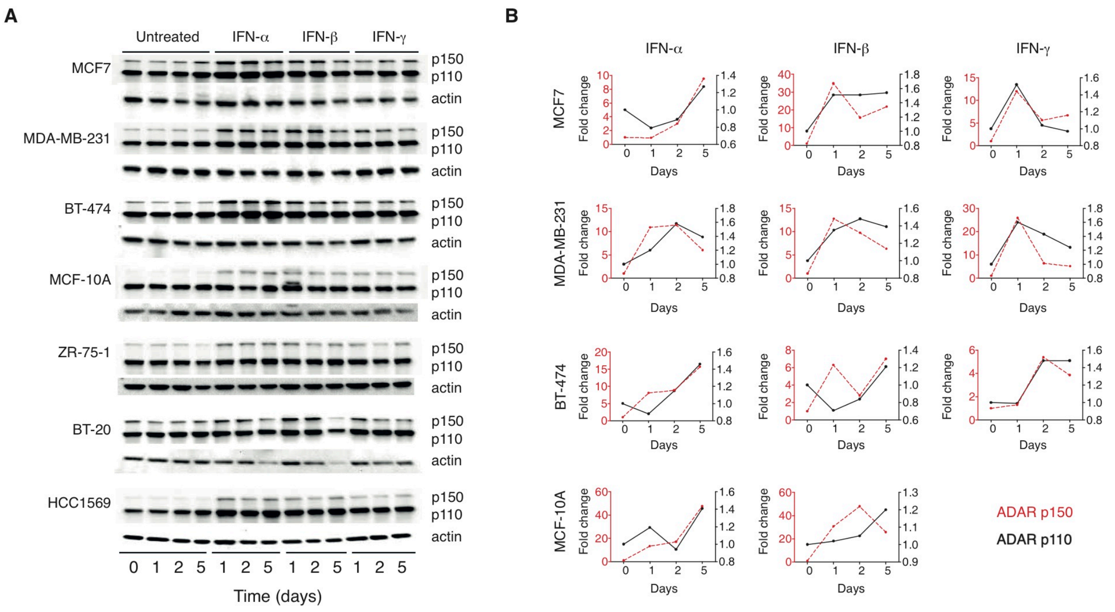

Figure S7. Interferon treatments increase *ADAR* mRNA and protein expressions in breast cancer cell lines (Related to Figure 5). (**A**), Western blots underlying Figure 5E and Table S6. (**B**), qRT-PCR for *ADAR* p110 and p150 (see also Table S7). The expressions of p110 and p150 are presented as fold changes relative to the expression of the untreated cells. Note the different *y*-axis scales used for the two isoforms. The scales for p150 are much larger.

Supplemental Tables

Supplemental tables are provided as online excel files.

- Table S1: **Patients data (Related to Figures 1-5**). Clinic-pathologic data of the patients involved in the study.

- Table S2: **Putative RNA-DNA differences (RDDs) in *in vivo* samples (Related to Figures 1-5)**. Characterization of the 16,027 RDDs identified in the patients under study. This data is necessary to reproduce most calculation in the paper.

- Table S3: **Sample data (Related to Figures 1-5)**. For each patient involved in the study, this table reports key information necessary to reproduce most analyses and figures in the paper.

- Table S4: **Comparison of (A) A-to-I RNA editing studies and (B) detection pipelines (Related to Figure 1).** These tables report the comparison between the current study and the most relevant ones published in the field in the last years with what regards (A) their features and (B) their detection pipelines.

- Table S5: **AZIN1 editing measured by amplicon sequencing in 30 patient-matched tumor/normal pairs (Related to Figure 2)**. Values representing the editing of AZIN1 measured by amplicon sequencing (Roche FLX) in tumor and normal matched pairs of 30 study patients.

- Table S6: **ADAR protein expression quantification (A) and editing frequency (B) in *in vitro* IFN experiments (Related to Figures 4 and 5).** For cell lines treated with IFN α, β and γ for 24h, 48h and 120h, these tables report: (A) the ADAR protein expression determined by Western blot, and (B) the editing frequency of the sites investigated with amplicon sequencing.

- Table S7: **ADAR RT-PCR mRNA expression in *in vitro* IFN experiments Related to Figures 4 and 5).** For cell lines treated with IFN α, β and γ for 24h, 48h and 120h, this table reports the expression of ADAR determined with RT-PCR.

Supplemental Experimental Procedures

Patients and samples characterization and preparation

Samples selection. A total of 58 breast cancer (BC) patients for whom both fresh-frozen tumor and matched normal breast tissue, as well as formalin-fixed paraffin embedded (FFPE) matched tumor breast tissue were available at Jules Bordet Institute Tumor Bank (Jules Bordet Institute, Brussels, Belgium) were selected for this project. Patients were recruited between 2007 and 2011; the associated clinico-pathological data can be found in Table S1.

The use of the data is consistent with the informed consent signed by the patients or has been granted ethical approval by the local Ethics Committee and is in accordance with the applicable laws and regulations of Belgium. The study has been approved by the local Ethics Committee (approval number: CE1967).

Samples histopathology. On the basis of their immunohistochemistry (IHC) profile, patients were classified in one of the principle IHC BC subtypes: triple negative (TN: estrogen receptor (ER), progesterone receptor (PgR), and human epidermal growth factor receptor 2 (HER2) negative), HER2 positive (any ER and PgR, HER2 positive), luminal A (ER positive, HER2 negative, histological grade 1) and luminal B (ER positive, HER2 negative, histological grade 3).

The ER and PgR status was defined using the anti-estrogen receptor antibody [SP1] (ab166600, Abcam®, Cambridge, UK) and the anti-progesterone receptor antibody [1E2] (Roche, Basel, Switzerland), respectively. The staining was scored according to Allred (Harvey et al., 1999; Leake et al., 2000) using a combined score for proportion and intensity, and was considered as positive if the global score was >2. The HER2 status was defined using the antiHER2/neu antibody (4B5) (Roche). The scoring and subsequent FISH-analyses were done in accordance to the ASCO-CAP Guidelines on HER2-testing (Wolff et al., 2007). The histological grade was defined using the modified Bloom-Richardson grading system (Elston and Ellis, 1991; Genestie et al., 1998). The Ki67 staining was performed using the Monoclonal Mouse Anti-Human Ki-67 Antigen (Clone MIB-1) (Dako, Glostrup, Denmark).

For each sample, an hematoxylin and eosin (H&E) slide was made and was reviewed by a breast pathologist to confirm that the tumor specimen contained at least 30% of tumor cell nuclei and that the matched, adjacent normal specimen contained no tumor cells. Evaluation of the quantity and location (stromal or intratumoral) of tumor-infiltrating lymphocytes (TILs) was defined as described previously (Denkert et al., 2010).

DNA Extraction. DNA from both tumor and matched normal fresh-frozen tissues was extracted using the DNeasy Blood and Tissue kit® (Qiagen, Venlo, Netherlands) following the manufacturer's instructions. DNA concentration was measured using the NanoDrop 1000 instrument (Thermo Scientific, Waltham, Massachusetts). All the samples yielded enough material for downstream analyses.

RNA Extraction. RNA from both tumor and normal fresh-frozen tissues as well as from cell lines was extracted using TRIzol® (Life Technologies, Carlsbad, California) following the manufacturer's instructions. RNA concentration was defined using the NanoDrop 1000, and RNA integrity (RIN: RNA Integrity Number) was assessed using an Agilent 2100 Bioanalyzer (Agilent Technologies, Santa Clara, California).

All the samples yielded enough material for downstream analyses and had a RIN equal or superior to 6.5.

Purification of organoids from primary breast tissues. The protocol is described in details elsewhere (Allinen et al., 2004; Choudhury et al., 2013).

Detection of RNA-DNA differences

The analysis pipeline for the detection of RNA-DNA differences (RDDs) is summarized in Figure S1 and described in details in the following sections.

RNA Sequencing. Transcriptome sequencing was performed at DNAVision (Gosselie, Belgium). Transcriptome libraries from 58 tumor and 10 matched normal samples were constructed using the Illumina® TruSeq™ RNA Sample Preparation Kit for paired end reads sequencing on the HiSeq 2000 (Illumina, San Diego, California) following the manufacturer's instructions.

Briefly, starting from 1 μg of total RNA, the poly-A containing mRNA molecules were purified using poly-T oligo-attached magnetic beads. Following purification, the mRNA was fragmented into small pieces using divalent cations under elevated temperature. The cleaved RNA fragments were copied into first strand cDNA using reverse transcriptase and random primers. This was followed by second strand cDNA synthesis using DNA Polymerase I and RNase H and purification using the AMPure XP beads (Agencourt BioSciences Corporation, Beverly, Massachusetts). The cDNA fragments went through an end repair process, the addition of a single 'A' base and ligation of the adapters. The products were purified using the AMPure XP beads and enriched with PCR (15 cycles) to create the final cDNA library followed by purification using the AMPure XP beads. Libraries' quality control and quantification were performed using the Agilent Bioanalyser 2100 and qRT-PCR; libraries were pooled (4 libraries/pool). Clusters were generated in a cBot Cluster Generation System using the Paired-End Cluster Generation Kit v2-HS and sequenced on the Illumina HiSeq 2000 platform with a 2x50 base-pairs (BP) paired-end mode.

Exome Sequencing. Exome sequencing was performed at GATC (Konstanz, Germany). Genomic libraries from the tumor and matched normal samples were generated using the Illumina Paired End DNA sample preparation kit (Illumina) following the manufacturer's instructions. Enrichment was performed using the Agilent SureSelect Human All Exon V3 kit (Agilent) following the manufacturer's instructions.

Briefly, 2-3 μg of total genomic DNA was randomly fragmented to between 150 and 600bp by focused acoustic shearing (Covaris Inc, Wouburn, Massachusetts). A cleanup was performed using AMPure beads (Agencourt BioSciences Corporation) following the manufacturer's protocol and quality of the material was assessed using the Agilent Bioanalyser 2100. The size fractionated DNA was end repaired using T4 DNA polymerase, Klenow polymerase and T4 polynucleotide kinase and purified using AMPure beads. The resulting blunt ended fragments were A-tailed using a 3′-5′ exonuclease-deficient Klenow fragment, purified using AMPure beads and ligated to Illumina paired-end adaptor oligonucleotides in a 'TA' ligation at 20°C for 15 minutes. The product was purified using AMPure beads. After estimation of the concentration, the adaptor-ligated library was amplified and then purified using AMPure beads. Quality and quantity were assessed using an Agilent 2100 Bioanalyzer. The enriched regions were captured, purified, PCR amplified and purified using AMPure beads. After quantification and quality control of the captured library, samples were pooled (four samples/lane) for loading on an Illumina HiSeq

2000. Samples were sequenced in paired-end mode, with a read length of 2x100 bases.

Transcriptome read mapping. Because transcriptome read mapping is a key step to identify differences between RNA and DNA, we designed a dedicated framework to handle common errors associated to spliced reads. RNA reads were mapped simultaneously on the human reference genome (hg19) and a dedicated library of splice junction sequences using the Burrows-Wheeler Aligner (Li and Durbin, 2010) (BWA v0.5.9). We chose the BWA aligner due to its ability to handle gapped alignment and to report multiple matches for each read, which is required to identify reads mapping equally to the genome and the corresponding splice junction or to solve ambiguous read pairing. Paired reads were mapped independently with the command '`bwa aln –n 6`' to report up to 6 matches for reads that can be aligned to multiple places. Splice junctions were designed by concatenating respectively the last and first 50 nucleotides for each pair of consecutive exons. We used gene annotations from Refseq, UCSC, Ensembl and Gencode, downloaded from the UCSC Table Browser (Karolchik et al., 2004). Junctions common to two or more annotation sources were added only once to the library. Also, because BWA concatenates all chromosome sequences before indexing the reference, buffers of 20 N letters were added at each extremity of the splice junctions. This prevents BWA from producing irrelevant alignments extending outside the boundaries of reference sequences. For exons shorter than 50 nucleotides, this procedure would add adjacent intronic bases immediately upstream or downstream of this exon to meet the required splice sequence length. Such additions could further introduce inaccurate mapping and recurrent alignment errors around splice junctions. To solve this issue, we used an incremental approach to create splice site sequences, allowing as many exons as necessary to meet the required sequence length of 100 nucleotides. After alignment, coordinates of reads mapped on splice junctions were converted to the hg19 coordinate system.

Trimmed RNA reads could be shorter than 50 bases, thus some could be equally placed on a splice junction and its genomic counterpart. In this case, unique matches could be mistaken with repeats and incorrectly discarded. After alignment, all matches reported for a given read were processed to remove those that were identical once alignments on splice sites were reverted back to hg19 coordinates.

Paired-end sequencing often implies a pair rescue step in which ambiguous alignments can be fixed based on strand orientation and distance between mates. However, in the context of RNA sequencing, this procedure can sometimes introduce recurrent mismatches. When paired reads are processed independently, 'bwa aln' uniquely map them on the correct genomic location. However, when running 'bwa sampe' to perform read pairing, incorrect alignments can be preferred if they form a pair matching the expected insert size and strand orientation, even in presence of multiple mismatches. This mainly occurs for processed pseudogenes, because 'bwa sampe' does not compute the distance between mates with regard to the skipped introns spanned by transcriptome reads.

To solve this issue without losing the benefit of paired-end information, we implemented our own read-pairing step similar to 'bwa sampe'. For each read pair for which either one or both mates could not be uniquely mapped, we considered all possible correct pairings minimizing the cumulative edit distance over both mates. Read pairing was considered correct if strand orientation and inner distance between mates, after subtraction of intronic sequences between them, matched the Illumina

sequencing protocol. If multiple best pairings were found, both mates were flagged as repeats and discarded. Otherwise, the best pair was selected and both reads were considered unique. During this step, reads identified as repeats when mapped independently could be recovered as unique if they belonged to a single best pairing. However, unlike 'bwa sampe', we did not implement a Smith-Waterman local alignment to rescue read pairs where only one mate could not be mapped.

Once non-canonical read pairs were discarded, duplicates were removed with Picard's MarkDuplicates utility (v1.59) (http://broadinstitute.github.io/picard/) with default parameters and reads were realigned locally using the GATK's IndelRealigner program (v1.4-15) (McKenna et al., 2010). Local realignment was run with options '--knownAlleles known.indels.vcf --consensusDeterminationModel USE_READS' --maxConsensuses 50 --maxReadsForRealignment 400000 --maxReadsInMemory 300000', where known.indels.vcf was downloaded from the GATK resource bundle.

Exome read mapping. Paired-end reads from exome sequencing were mapped to the hg19 reference genome using BWA with default settings. As for transcriptome reads, only concordant unique read pairs were used. Duplicates were further removed using Picard and remaining reads were realigned locally with GATK. Both programs were used with the same parameters as for transcriptome alignments.

Identification of RNA-DNA differences. We identified single nucleotide substitutions based on pileup alignments. Pileup was computed using SAMtools (v0.1.18) (Li et al., 2009) with the command '`samtools mpileup -B -D -d 100000 -f hg19.fa in.bam | pileup-to-vcf.pl`', where pileup-to-vcf.pl was an in-house program designed to call a variant if the following conditions are met at a given position: 1) minimum depth of 10 reads, 2) minimum alternate allele frequency of 10 percent, 3) a minimum of 2 confident mismatches with base quality equal or greater to 25, 4) located in reads with a mapping quality of 20 or more and 5) not within the first of last 6 nucleotides of this read. Variants were then filtered based on strand bias and distance to read ends, to discard low confidence candidates relying on mismatches whose position relative to the query sequence harbors a suspicious pattern. Substitutions were further identified as RDDs if the corresponding position in DNA was homozygous for the reference. This implied 6) a minimum coverage of 10 reads in DNA, 7) a fraction of reference base equal to or greater than 95 percents and 8) allowing a maximum of 2 mismatches. However, because recent studies show that some dbSNP entries correspond to RNA edits (Eisenberg et al., 2005), we did not remove RDDs matching a known record from dbSNP (Sherry et al., 2001) v135.

Recovery of clustered RDDs. Due to the low coverage in certain regions of our transcriptome datasets, many rare edits were lost considering our minimum depth and frequency thresholds. Previous studies (Ju et al., 2011; Peng et al., 2012; Ramaswami et al., 2012) also report that a large fraction of RNA edits are located within untranslated region of genes, which are poorly covered by exome libraries. ADAR mediated editing is known to operate on double-stranded RNA duplexes often caused by the presence of inverted Alu elements. As a consequence, RNA edits are often clustered in genomic regions corresponding to both strands of the latter duplexes. Based on the hypothesis that multiple edits are likely to be close one from each other, we added an additional step to our pipeline to rescue rare edits clustered with high confidence candidates. We selected all edits called within Alu elements, based on UCSC RepeatMasker (Smit et al.), and searched for identical substitutions in the same genomic region. We iteratively screened genomic intervals of 15 nucleotides

immediately upstream and downstream of each confident RDD, these intervals being extended until no more additional edits could be rescued.

Cohort-wise integration of editing sites. Per-sample edits identified as described in the previous sections were then pooled together and fraction of edited bases for each sample was recomputed directly from pileup alignment in each samples in the cohort. This allowed rare edits detected only in highly covered transcriptomes to be rescued in other samples.

Excluded regions. The majority of the RDDs (10,254) clustered in 8.6Mb spread across the following five regions that did not overlap the Alu regions.

Name	Chrom	Strand	txStart	txEnd
BL_1	chr6	*	28477796	33448353
uc010yts.2	chr2	+	89890561	90471176
uc021vkt.1	chr2	-	89156873	89630175
uc021vku.1	chr2	+	89185067	89595920
uc021ser.1	chr14	-	105994255	107283085
uc021wml.1	chr22	+	22385571	23265082

These five regions encompassed immunoglobulin variable regions and HLA genes, suggesting alignment artifacts and/or a potential immunological effect unrelated to A-to-I editing. Therefore, we did not consider these RDDs, but used them as negative controls in several of our A-to-I editing analyses.

DNA-free A-to-I editing detection pipeline. we downloaded a list of known A-to-I editing sites for hg19 from the RADAR database (Ramaswami and Li, 2014). REDItoolKnown, which is part of the REDItools package (Picardi and Pesole, 2013), was then invoked with default settings for each of the 68 RNA-seq samples (`REDItoolKnown.py -i sample.bam -f hg19.fa -l RADAR.tab -u -o sample.edits.txt`). The output was a list of positions of known editing sites for which editing is callable, but not necessarily present, in the sample at hand. Per-sample lists of edits were merged, resulting in a total of 115,087 non-redundant genomic positions callable in at least one sample. The number of sites for which at least one read across the entire cohort documented an editing event was 59,993. Importantly, this pipeline did not use our DNA exome sequences, and therefore is not limited by the small fraction of the genome they cover—dramatically extending the number of callable basis. Among the 59,993 events, 50,918 were from Alu regions.

Measure of AZIN1 editing in tissues with the Roche FLX sequencer. A region containing the edited site of *AZIN1* was amplified using designed fusion oligonucleotide primers (Forward 5'-ACCGGAAGTGATGAACCAGCCT-3' and Reverse 5'-GCTGAATGCAAGAAGGCACAAAGA-3' specific sequences). For each patient sample, PCR was performed on 50 ng of cDNA using the Platinum PCR System (Life Technologies Europe B.V., Gent, Belgium) and standard Touch-Down thermocycling conditions (2 min denaturation at 94°C, followed by 20 cycles of denaturation for 30 s at 94°C, annealing for 30 s at 65°C* and extension for 30 s at 72°C (*with decrements of 0,5c° annealing temperature at the completion of each cycle), 20 cycles of denaturation for 30 s at 94°C, annealing for 30 s at 55°C and extension for 30 s at 72°C, and final extension for 6 min at 72°C). The fused primers

each contained a common 20-bp region at their 5'-end that is used in Multiplex Identifiers labeling, clonal amplification and sequencing on a 454 Genome Sequencer FLX system as described by manufacturer (Roche Applied Sciences, Indianapolis, USA).

After removing primer and adapter sequences, 454 reads were mapped on the reference genome (hg19) using the BLAT program (Kent, 2002) due to its ability to handle long spliced reads. Blat was invoked with the following command: '`blat -stepSize=5 hg19.fa reads.fasta out.psl`'. Editing at position chr8:103,841,636 was then computed for each sample based on pileup alignment.

Protein expression, mRNA expression and DNA copy number profiling

ADAR IHC. For each sample, a representative FFPE block containing invasive adenocarcinoma, including whenever possible a corresponding ductal carcinoma *in situ*-component, lymphocytes and normal ductal epithelium cells, was selected.

ADAR IHC was performed as follows: briefly, sections were de-paraffinized and processed using the Ventana detection system with the iView™ DAB detection kit (Ventana, Tucson, Arizona). Antigen retrieval was performed with EDTA (Tris/borate/EDTA; pH 8.4). The slides were then incubated in a 1:50 dilution of mouse polyclonal anti-ADAR antibody (Abcam, ab88574) at room temperature for 28 minutes. After staining, slides were processed in accordance with routine protocols. A representative slide was chosen and scanned with a NanoZoomer 2.0RS scan (Hamamatsu Photonics Hamamatsu-SHI, Japan) in 40x mode using the NDP.scan software.

Quantitative real-time PCR (qRT-PCR). In order to analyze the expression of *ADAR* p110, *ADAR* p150, total *ADAR* expression in both clinical samples and cell lines, we first reverse-transcribed 500 ng of total RNA using the High Capacity RNA-to-cDNA kit (Applied Biosystems, Foster City, CA) following the manufacturer's instructions. qRT-PCR was performed according to the TaqMan Gene Expression Assay protocol (Applied Biosystems) using the following primers: *ADAR* p110: forward, 5'-GGCAGTCTCCGGGTG -3', reverse 5'- CTGTCTGTGCTCATAGCCTTGA-3', FAM probe: 5'-CCGGCCGTGTCCCGAGGA-3'; *ADAR* p150: forward, 5'-CTTCCAGTGCGGAGTAGCG-3', reverse 5'- GTGACGGTGTCTGCTTTCCA-3', FAM probe: 5'- TCGGGCCAGGGTCGTGCC- 3'. For the quantification of total *ADAR*, we used commercially available primers and probe (Hs00241666_m1, Life Technologies). *GUSB* (Hs99999908_m1, Life Technologies) and *TBP* (Hs00427621_m1, Life Technologies) were used as reference genes. Real-time PCR was performed on a 7900HT Sequence Detection System (Applied Biosystems). All reactions were run in duplicate.

Gene expression from RNA-seq data. RNA-seq data were generated and aligned on the human genome as described in previous sections. Expression was then estimated from the BAM files using Cufflinks v2.0.0 (Trapnell et al., 2012) with options `-N -u --GTF`. The transcript database provided with the `--GTF` option was ENSEMBL GRCh37.65. The expression FPKM (Fragment Per Kilobase per Million aligned reads) values, x, generated by Cufflinks were set to non null values and $\log_2$-transformed with the formula $f(x) = \log_2(x+1)$.

Gene expression from Affymetrix® array. 100 ng of total RNA was profiled using the Affymetrix® HG-U133 Plus 2.0 Arrays (Affymetrix®, Santa Clara, California), following the manufacturer's instructions. Briefly, the RNA was first reverse-transcribed into double-stranded cDNA. This cDNA was transcribed in vitro. After

purification of the aRNA, 12.5 µg were fragmented and labeled prior to hybridization to the arrays. Quality control (QC) for each chip was performed following the recommendations posted on http://www.arrayanalysis.org/.

CEL files were normalized with fRMA (McCall et al., 2010) v1.8.0 for R (R Development Core Team) v2.15.1. Probes were annotated from the ENSEMBL transcript database (same version as above) using BioMart (Smedley et al., 2009) v2.12.0. The best probe for a given gene was selected with Jetset (Li et al., 2011) v0.99.3.

When needed, RNA-seq and Affymetrix data were matched gene-wise on the basis of HUGO gene symbols.

Genome Wide SNP analysis. Genome wide SNP analysis was performed at AROS Applied biotechnologies a/s (Aarhus, Denmark) on Affymetrix Genome-Wide Human SNP Arrays 6.0 (Affymetrix) following the manufacturer's instructions. Briefly, 500 ng of genomic DNA was digested with either Nsp I or Sty I and then ligated to adapters that recognize the cohesive four-basepair (bp) overhangs. A generic primer that recognizes the adapter sequence was used to amplify adapter ligated DNA fragments, with PCR conditions optimized to preferentially amplify fragments in the 200 to 1,100 bp size range in a GeneAmp PCR System 9700 (Applied Biosystems). After purification and quantification, a total of 45 µl of PCR product was fragmented and a sample of the fragmented product was visualized on a 4% TBE agarose gel to confirm that the average size was smaller than 180 bp. The fragmented DNA was labeled with biotin and hybridized to the GeneChip Mapping Panels for 18 hrs. Arrays were washed and stained using an Affymetrix fluidics Station 450 and scanned using a GeneChip Scanner 3000 7G (Affymetrix). The Affymetrix GeneChip Operating Software (GCOS) was used to collect and extract feature data from the Affymetrix GeneChip Scanner.

The Affymetrix Genome-Wide Human SNP 6.0 arrays were normalized for technical variation between chips using the copy number workflow of Affymetrix Power Tools release v1.14.3. We used the full version of the CDF, version na.32 of NetAffx's annotation database for SNP 6.0 and version na.32 r1 of the HapMap 270 reference file. We ran the procedure with the default parameter settings. The raw $\log_2$ ratios from above were segmented using the circular binary segmentation algorithm (Olshen et al., 2004) implemented in the R/Bioconductor package DNAcopy version v1.34.0. We applied the full permutation method with default parameter settings, except `undo.splits="sdundo", undo.SD=2`. The segmented $\log_2$ ratios were used as input to a two-level hierarchical mixture model as described by van de Wiel et al. (van de Wiel et al., 2007) and implemented in the R package CGHcall version v2.20.0. Default parameter settings were used expect for `prior="not all", nclass=4`.

Cell lines experiments

Cell culture and Interferon treatment. MCF7 (ATCC® HTB22™), MDA-MB-231 (ATCC® HTB26™), BT-474 (ATCC® HTB20™), MCF-10A (ATCC® CRL10317™), ZR-75-1 (ATCC® CRL1500™), BT-20 (ATCC® HTB19™) and HCC1569 (ATCC® CRL2330™) breast cells lines were obtained from ATCC (Manassas, Virginia) in December 2012 and cultured under standard conditions. All cell lines were regularly authenticated by morphological observation and tested for mycoplasma contamination (MycoAlert, Rockland, Maryland) before performing the

experiments described below. The cells were incubated at 37 °C in a humidified incubator containing 5% CO_2.

MCF7 and ZR-75-1 are ER+, HER2- tumor cell lines; MDA-MB-231 and BT-20 are ER- HER2- tumor cell lines; BT-474 is an ER+ HER2+ tumor cell line and HCC1569 is HER2+ ER-. MCF-10A is an immortalized, non-transformed mammary epithelial cell line. Where indicated, cell lines were treated with the following doses of interferon (IFN): 1000UI/ml of Universal Type I IFN (Recombinant Human IFN-alpha A/D [BglII]) (IFN-α; cat# 11200-1, R&D Systems, Minneapolis, Minnesota), 1000UI/ml of Recombinant Human IFN-beta 1a (Mammalian) (IFN-β; cat# 11415-1; R&D Systems) or 500UI/ml of Recombinant Human IFN-gamma (IFN-γ; cat# 285-IF-100; R&D Systems). Cells were treated for 24h (1 day), 48h (2 days) and 120h (5 days); parallel cultures were left untreated as controls.

Lentiviral transduction. ADAR gene expression inhibition was performed using transduction-ready lentiviral particles containing 3 target-specific constructs encoding shRNA specifically designed to knock down ADAR expression. Control shRNA lentiviral particles containing a scrambled shRNA were used as a negative control for experiments. MDA-MB-231, MCF7 and BT474 cells transduction were performed accordingly to manufacturer's instructions (Santa Cruz biotechnology, Texas).

Cell proliferation assay. Cell proliferation was determined by 3-(4,5-dimethylthiazole-2-yl)-2.5 diphenyltetrazolium bromide assay (MTT, Sigma). All cells were seeded at a density of 6000 cells per well. At each time point, 25 µl of 5 mg/ml MTT was added and incubated at 37°C for 3.5 h and 100 µl DMSO was added to the wells. Every 24 hours, the rate of cellular proliferation was determined by measuring the absorbance at 590 nm. Cell growth curves were calculated as mean values after normalization to the absorbance at day 1 from 3 independent experiments comprising each six replicates. Difference in cell growth was considered as significant when $p<0.05$ according to a paired t test.

Apoptosis assessment. Apoptotic cell percentage was evaluated using the PE-Annexin-V Apoptosis Detection kit I (BD Pharmingen, San Diego, CA) following the manufacturer's instructions. Briefly, cells were double stained with Annexin V and 7-AAD and were then analyzed by flow cytometry. Apoptotic cells were defined as Annexin V positive cells including Annexin V^+/ 7-AAD^- cells (early apoptosis) and Annexin V^+/7-AAD^+ cells (late apoptose). Difference in apoptosis was considered as significant when $p<0.05$ according to a paired t test.

Western blot analysis. Cells were lysed in a buffer (NaCl 150mM, Tris-HCl 50mM, NP40 1%, SDS 20%, EDTA 5mM, protease inhibitors cocktail) at 4°C for 30 minutes. Protein concentrations were determined using the Pierce™ BCA Protein Assay kit (Thermo Scientific). Equal amounts of proteins (10µg) were separated on 4-12% Bis-Tris gels, transferred to nitrocellullose membranes, blocked with TBST buffer (50 mM Tris pH 8.0, 150 mM NaCl, 0.1% Tween 20) containing 5% nonfat milk, washed with TBST buffer, and incubated overnight at 4°C with primary antibodies against ADAR1 antibody (Cat#12317S, Cell Signaling, Danvers, Massachusetts) at a dilution of 1:1000, and against Actin, Clone 4 (Cat# MAB1501R, Millipore, Billerica, Massachusetts), at a dilution of 1:5000. The membranes were then washed in TBST four times, incubated with HRP-conjugated secondary antibodies for 2 h at RT and washed in TBST buffer four times. Proteins were detected using the Western lightning Ultra system (Perkin Elmer, Waltham, Massachusetts). The immunoblot signals were visualized with a chemiluminescence system (Biorad, Hercules, California) and quantified using Biolab 4.0.1 software.

<u>*qRT-PCR analysis.*</u> The extraction, quantification and quality control of the RNA extracted from cell lines was performed as described above. Only four cell lines gave enough quality and quantity material for downstream analyses (MCF7, MDA-MB-231, BT-474, and MCF-10A). For the analysis of the data obtained with qRT-PCR, relative expression of the genes of interest to *GUSB* and *TBP* was calculated using the $2^{-\Delta Ct}$ method. This normalized expression level allowed to determine the fold changes in the expression of the genes of interest between different subgroups.

<u>*Processing of Roche FLX read mapping.*</u> Only four cell lines gave enough quality and quantity of material for downstream analyses (MCF7, MDA-MB-231, BT-474, and MCF-10A). Sequencing was performed as explained for the *AZIN1* amplicon. The following primers were used:

AZIN1ex11-13_F (Tag: AAGACTCGGCAGCATCTCCA; Specific Sequence: ACCGGAAGTGATGAACCAGCCT);

AZIN1ex11-13_R (Tag: GCGATCGTCACTGTTCTCCA; Specific Sequence: GCTGAATGCAAGAAGGCACAAAGA);

BPNT1_Alu1_F (Tag: AAGACTCGGCAGCATCTCCA; Specific Sequence: CCAATTGACAGTTCAGGTCAATGTTC);

BPNT1_Alu1_R (Tag: GCGATCGTCACTGTTCTCCA; Specific Sequence: AAAATTGTGCCCTAAAGAAATCTGG);

MRPS16_Alu_F (Tag: AAGACTCGGCAGCATCTCCA; Specific Sequence: TTCCCATGTGTTTTAAAAGCCTGAA);

MRPS16_Alu_R (Tag: GCGATCGTCACTGTTCTCCA; Specific Sequence: GCCAAATTATGTAATGTTTTCTTTTTC);

BPNT1_Alu2_F (Tag: AAGACTCGGCAGCATCTCCA; Specific Sequence: GCCGAGTTCCAGAATCTATTAAAAATG);

BPNT1_Alu2_R (Tag: GCGATCGTCACTGTTCTCCA; Specific Sequence: TCTTTCTCCTAGCTAAGTAAATGAAACTT);

ZDHHC20_Alu_F (Tag: AAGACTCGGCAGCATCTCCA; Specific Sequence: AAATCACTTTTCATTCACCCAATAAA);

ZDHHC20_Alu_R (Tag: GCGATCGTCACTGTTCTCCA; Specific sequence: GGCCAAATTATAAACAAATTATAAACCT).

A total of 39 samples, each corresponding to a specific combination of cell line, interferon treatment and duration, were multiplexed on a single 454 sequencing run. We first extracted per-sample amplicons and trimmed MID sequences at each ends of sequencing reads. During this step, we required that the read sequence start by the MID and ends by its reverse complement, for a total read length of 250bp, primer sequences included. This ensures that most reads retained for alignment correspond to amplicons sequenced at full length. Adaptor and primer sequences were further removed and reads were mapped on the reference genome with the bwasw (v0.5.9) aligner (Li and Durbin, 2010) with default parameters.

Once reads were mapped on the reference genome, edited positions were identified based on pileup alignment. Only target regions were screened for RNA editing. Mapped bases at each position were obtained using the SAMtools (v0.1.16) mpileup program (Li et al., 2009) called with the following command line: '`samtools mpileup -B -D -r range -q 0 -Q 0 -f hg19.fa aln.bam > out.pileup`', where range corresponds to regions targeted for amplicon sequencing. Since read depth was greatly superior compared to our whole

transcriptome datasets, we considered a position as edited if the number of non-reference bases was at least 10, so that low frequency editing events could be detected. Additionally, as target regions correspond to UTRs of genes transcribed on the reverse strand, we restricted identification of editing to positions where the reference base was a T and non-reference bases were Cs.

To investigate the incidence of coverage over the detection of RNA editing, we estimated the mean number of detected edits for different read depth. We first examined in what extend alignments could be downsampled. The only genomic region having a very high coverage (> 2,000 reads) in every sample was the *Alu* region located within *MRPS16* gene (chr10:75008708-75008970). For each sample, we then generated 10 replicates of the original alignment at specified depth D by randomly selecting D reads mapped on *MRPS16 Alu* region. We computed the mean number of edits detected across all downsampled replicates based on pileup alignment (10 or more edited bases at a given position). Note that this downsampling makes sense only for amplicon sequenced at full length and covering the whole target region, since in this case, number of mapped reads and read depth are equivalent.

Long 454 amplicons allow for the analysis of editability on a per-read basis. The main hypothesis is that if a given genomic position P_i is more often edited than another position P_j located in the same region (and consequently covered by the same amplicon sequence), then we can expect that P_j will be edited in amplicons where P_i has already been edited. In other words, if a given amplicon harbors a total of N edited positions, these should correspond to the N most edited positions across all amplicon sequences covering this region.

To verify this hypothesis, we extracted amplicon sequences mapped on each target *Alu* and overlapping all editable positions detected within this *Alu*. We then determined which positions were edited for each amplicon individually. This produces a binary matrix M for each target Alu, where $M_{i,j} = 1$ if position i is edited in amplicon j, 0 otherwise. Reads were further ordered from the highest edited to the one harboring the lowest number of edited bases. Similarly, genomic positions were also sorted from the most edited to the least edited.

Statistical Analysis

All computations were implemented in R (R Development Core Team) v2.15.1 and Bioconductor (Gentleman et al., 2004) 2.10. Defaults functions' parameters were used unless specified otherwise.

Third party data. Our analysis rests on a number of public domain data sets:

- *Alu* were located from the RepeatMasker (Smit et al.) downloaded from UCSC (http://genome.ucsc.edu).
- The DARNED database (Kiran and Baranov, 2010) for hg19 was downloaded from darned.ucc.ie.
- The RNA editing sites from the GM12878 lymphoblastoid were obtained from the Supplemental table of Ramaswamy et al. (Ramaswami et al., 2012).
- RNA-seq data from the TCGA were downloaded from the public access repository on 08/02/2013 and assembled using custom R scripts whilst CBS segmented $\log_2$ ratios for matching samples were downloaded.

Calculation of editing frequencies. For all RDD sites determined by the pipeline described in Figure S1, editing frequency was defined for each sample as the ratio of

the number of RNA-seq reads documenting the non-reference base by the total number of reads covering the site. Since gene expression varied from sample to sample, some RDD sites were not covered in some samples. In such cases, the editing frequency was considered undefined (i.e., 'NA' in R's terminology).

Statistical tests and related graphics. Spearman correlation coefficients, ρ, and corresponding p-values were calculated with R's cor.test function. All group comparisons were evaluated with the Wilcoxon tests as implemented by R's wilcox.test. All tests were two-sided, except for the paired comparison, which were single-sided. The multivariate analyses were performed with R's lm. Correlations coefficients and p-values were rounded to the nearest one significant digit number with R's signif. Because cancer is a heterogeneous disease, revealing the variability of statistics is essential. We displayed all individual sample-level data points for almost all the analyses conducted in this study in the form of scatter plots or strip charts with overlaid boxplots. Numerical data underlying each plot are provided as Supplemental Tables.

Confirmation of editing sites. Since our sequencing protocol was unstranded, RDDs were considered confirmed in the DARNED database if we could find a RDD in DARNED with the same genomic position and the same or reverse complement substitution.

The genomic positions of all putative RDDs and 1,000 random positions within 1,000 randomly selected *Alu* were sent to the Sanger Institute team (A.S. and P.C.). No other information was provided in order to avoid confirmation bias. They then computed DNA and RNA allelic frequencies at these putative RDD and random control positions in 15 breasts cancers. Tumor DNA sequences are described elsewhere (Nik-Zainal et al., 2012a, 2012b). RNA sequences were obtained from 2x75bp paired-end HiSeq 2000 Illumina sequencing with each sample run on two lanes. RNA-seq reads were aligned with TopHat (Trapnell et al., 2009, 2012) 1.3 in unsupervised mode (i.e., no transcript database provided to guide the alignment). The resultant binary alignment files (BAM) were merged then duplicates were removed with Picard (http://broadinstitute.github.io/picard/) MarkDuplicates. Multi-mapped reads were excluded from this analysis. SAMtools (Li et al., 2009) and a custom script were used to determine the allelic frequencies of each putative RDD. Hence, the data generation and computational processing of the validation samples differed substantially from those used to derive the original RDDs, reinforcing the value of the confirmation. The computed allelic frequencies were sent back to the Brussels team for comparison to the original RDDs. A RDD was considered confirmed if there was at least one sample with 1) a coverage >20 reads in both DNA and RNA at the RDD position, 2) an alternative allele identical to the original RDD in >2% of the RNA-seq reads, 3) The reference allele present in >95% on the DNA reads.

Editing frequencies heatmap. Rows and columns were ordered by increasing row-wide and column-wise mean editing frequencies. Contour lines were drawn from the smoothing of the resulting frequencies matrix. Smoothing was computed with the image.smooth function from R's fields package v6.7 with scale parameter theta=3.

Logistic dose-response fitting. Dose-response curves were established from the site-specific editing frequencies shown in Figure 5F and matched ADAR protein expression shown in Figure 5E (data in Supplemental Table S6). We included the two ADAR isoforms as (p150 + p110)/actin. We filtered out sites with less than 4 data points and for which the editing frequency at the lower ADAR expression was below 0.025. This filter excluded from the analysis sites for which trivial detection artifacts

caused departure from the logistic model. The dose-response curve of each editing site was then fit to the model with the `drm` function (with argument `fct= L.5(fixed=c(-1,0,NA,NA,1))`, i.e. the two-parameters logistic model, $f(x)=\varepsilon_i/(1+\exp(\omega_i-x))$) from R package drc (Ritz and Streibig, 2005) v2.5.12.

DNA-based statistical model of editability. RNA editing sites from biological sample GM12878 (see 'Third party data' above) were filtered to retain editing events falling within Alu regions and covered at a depth >20x. 51,621 sites passed this filter. They were ordered by order of appearance in the human genome. The first half of them were assigned to the training set, the second half to the validation set. Note that because editing sites tend to cluster per *Alu*, assigning them randomly to the training and validation sets would not guaranty independence of these sets.

The training set was used to derive DNA sequence features associated with the RNA editing ratio. We found highly significant association with the following variables defined on a per-site basis:

- Smith-Waterman alignment score of the 51bp hg19 DNA sequence centered on the edit site within the 2,501bp DNA sequence, also centered on the edit site, but on the opposite strand. We computed the alignment with the pairwiseAlignment function from Bioconductor package GenomicRanges v1.8.13 using the local-global mode with mismatch penalty of -3 and default parameters of function nucleotideSubstitutionMatrix.
- The distance between the best Smith-Waterman alignment and the edit site.
- The 20 nucleotides surrounding the edit site.

The edit ratios in the training set were then modeled with these 1+1+20=22 variables using a linear model as implemented by R's lm function. We attempted to use alternative parameters, e.g. larger windows around the edited sites and compute models with RBF kernel support vector machines, but did not obtain radically better fits of the training data.

Finally, the linear model was used to estimate the editability scores of the validation editing sites shown in Figure 4J.

Gene set analysis. We derived the genes whose expression had a strong positive correlation with the mean editing frequency by taking the intersection of the 250 genes the most positively correlated (Spearmans' ρ) with the mean editing frequency in the RNA-seq expression data, and the Affymetrix expression data. The intersection contained 85 genes.

The Affymetrix expression values were adjusted for *ADAR* amplification with a procedure akin to that of a previous publication (Venet et al., 2011). For each gene, expressions were fitted to the level of *ADAR* amplification determined from our Affymetrix SNP6.0 arrays. *ADAR* CN-adjusted expressions were then computed for each gene as the sum of its mean expression across the cohort and the residuals of the fit.

Adjusted Affymetrix data were then analyzed with the GSA (Efron and Tibshirani, 2007) package v1.03 for R, first using the 'canonical pathway' and then the 'transcription factor targets' gene sets from MSigDB (Liberzon et al., 2011; Subramanian et al., 2005) v3.1 (files c2.cp.v3.1.symbol.gmt and c3.tft.v3.1.symbol.gmt downloaded from www.broadinstitute.org/gsea). We searched for gene sets correlated with the patient-averaged editing frequency (using GSA's `resp.type="Quantitative"`).

Supplemental References

Allinen, M., Beroukhim, R., Cai, L., Brennan, C., Lahti-Domenici, J., Huang, H., Porter, D., Hu, M., Chin, L., Richardson, A., et al. (2004). Molecular characterization of the tumor microenvironment in breast cancer. Cancer Cell *6*, 17–32.

Choudhury, S., Almendro, V., Merino, V.F., Wu, Z., Maruyama, R., Su, Y., Martins, F.C., Fackler, M.J., Bessarabova, M., Kowalczyk, A., et al. (2013). Molecular Profiling of Human Mammary Gland Links Breast Cancer Risk to a p27(+) Cell Population with Progenitor Characteristics. Cell Stem Cell *13*, 117–130.

Denkert, C., Loibl, S., Noske, A., Roller, M., Müller, B.M., Komor, M., Budczies, J., Darb-Esfahani, S., Kronenwett, R., Hanusch, C., et al. (2010). Tumor-associated lymphocytes as an independent predictor of response to neoadjuvant chemotherapy in breast cancer. J. Clin. Oncol. Off. J. Am. Soc. Clin. Oncol. *28*, 105–113.

Eisenberg, E., Adamsky, K., Cohen, L., Amariglio, N., Hirshberg, A., Rechavi, G., and Levanon, E.Y. (2005). Identification of RNA editing sites in the SNP database. Nucleic Acids Res. *33*, 4612–4617.

Elston, C.W., and Ellis, I.O. (1991). Pathological prognostic factors in breast cancer. I. The value of histological grade in breast cancer: experience from a large study with long-term follow-up. Histopathology *19*, 403–410.

Genestie, C., Zafrani, B., Asselain, B., Fourquet, A., Rozan, S., Validire, P., Vincent-Salomon, A., and Sastre-Garau, X. (1998). Comparison of the prognostic value of Scarff-Bloom-Richardson and Nottingham histological grades in a series of 825 cases of breast cancer: major importance of the mitotic count as a component of both grading systems. Anticancer Res. *18*, 571–576.

Gentleman, R.C., Carey, V.J., Bates, D.M., Bolstad, B., Dettling, M., Dudoit, S., Ellis, B., Gautier, L., Ge, Y., Gentry, J., et al. (2004). Bioconductor: open software development for computational biology and bioinformatics. Genome Biol. *5*, R80.

Harvey, J.M., Clark, G.M., Osborne, C.K., and Allred, D.C. (1999). Estrogen receptor status by immunohistochemistry is superior to the ligand-binding assay for predicting response to adjuvant endocrine therapy in breast cancer. J. Clin. Oncol. Off. J. Am. Soc. Clin. Oncol. *17*, 1474–1481.

Karolchik, D., Hinrichs, A.S., Furey, T.S., Roskin, K.M., Sugnet, C.W., Haussler, D., and Kent, W.J. (2004). The UCSC Table Browser data retrieval tool. Nucleic Acids Res. *32*, D493–D496.

Kent, W.J. (2002). BLAT--the BLAST-like alignment tool. Genome Res. *12*, 656–664.

Leake, R., Barnes, D., Pinder, S., Ellis, I., Anderson, L., Anderson, T., Adamson, R., Rhodes, T., Miller, K., and Walker, R. (2000). Immunohistochemical detection of steroid receptors in breast cancer: a working protocol. UK Receptor Group, UK

NEQAS, The Scottish Breast Cancer Pathology Group, and The Receptor and Biomarker Study Group of the EORTC. J. Clin. Pathol. *53*, 634–635.

Li, H., and Durbin, R. (2010). Fast and accurate long-read alignment with Burrows-Wheeler transform. Bioinforma. Oxf. Engl. *26*, 589–595.

Li, H., Handsaker, B., Wysoker, A., Fennell, T., Ruan, J., Homer, N., Marth, G., Abecasis, G., and Durbin, R. (2009). The Sequence Alignment/Map format and SAMtools. Bioinforma. Oxf. Engl. *25*, 2078–2079.

Li, Q., Birkbak, N.J., Gyorffy, B., Szallasi, Z., and Eklund, A.C. (2011). Jetset: selecting the optimal microarray probe set to represent a gene. BMC Bioinformatics *12*, 474.

Liberzon, A., Subramanian, A., Pinchback, R., Thorvaldsdóttir, H., Tamayo, P., and Mesirov, J.P. (2011). Molecular signatures database (MSigDB) 3.0. Bioinforma. Oxf. Engl. *27*, 1739–1740.

McCall, M.N., Bolstad, B.M., and Irizarry, R.A. (2010). Frozen robust multiarray analysis (fRMA). Biostat. Oxf. Engl. *11*, 242–253.

McKenna, A., Hanna, M., Banks, E., Sivachenko, A., Cibulskis, K., Kernytsky, A., Garimella, K., Altshuler, D., Gabriel, S., Daly, M., et al. (2010). The Genome Analysis Toolkit: a MapReduce framework for analyzing next-generation DNA sequencing data. Genome Res. *20*, 1297–1303.

Nik-Zainal, S., Van Loo, P., Wedge, D.C., Alexandrov, L.B., Greenman, C.D., Lau, K.W., Raine, K., Jones, D., Marshall, J., Ramakrishna, M., et al. (2012b). The life history of 21 breast cancers. Cell *149*, 994–1007.

Olshen, A.B., Venkatraman, E.S., Lucito, R., and Wigler, M. (2004). Circular binary segmentation for the analysis of array-based DNA copy number data. Biostat. Oxf. Engl. *5*, 557–572.

Picardi, E., and Pesole, G. (2013). REDItools: high-throughput RNA editing detection made easy. Bioinformatics. *29*, 1813–1814.

R Development Core Team R: A Language and Environment for Statistical Computing. *1*, ISBN 3–900051 – 07–0.

Ritz, C., and Streibig, J. (2005). Bioassay Analysis Using R. J. Stat. Softw. *12*.

Sherry, S.T., Ward, M.H., Kholodov, M., Baker, J., Phan, L., Smigielski, E.M., and Sirotkin, K. (2001). dbSNP: the NCBI database of genetic variation. Nucleic Acids Res. *29*, 308–311.

Smedley, D., Haider, S., Ballester, B., Holland, R., London, D., Thorisson, G., and Kasprzyk, A. (2009). BioMart--biological queries made easy. BMC Genomics *10*, 22.

Smit, A., Hudley, R., and Green, P. RepeatMasker Open-3.0.

Subramanian, A., Tamayo, P., Mootha, V.K., Mukherjee, S., Ebert, B.L., Gillette, M.A., Paulovich, A., Pomeroy, S.L., Golub, T.R., Lander, E.S., et al. (2005). Gene set enrichment analysis: a knowledge-based approach for interpreting genome-wide expression profiles. Proc. Natl. Acad. Sci. U. S. A. *102*, 15545–15550.

Trapnell, C., Pachter, L., and Salzberg, S.L. (2009). TopHat: discovering splice junctions with RNA-Seq. Bioinforma. Oxf. Engl. *25*, 1105–1111.

Trapnell, C., Roberts, A., Goff, L., Pertea, G., Kim, D., Kelley, D.R., Pimentel, H., Salzberg, S.L., Rinn, J.L., and Pachter, L. (2012). Differential gene and transcript expression analysis of RNA-seq experiments with TopHat and Cufflinks. Nat. Protoc. *7*, 562–578.

Venet, D., Dumont, J.E., and Detours, V. (2011). Most random gene expression signatures are significantly associated with breast cancer outcome. PLoS Comput. Biol. *7*, e1002240.

Van de Wiel, M.A., Kim, K.I., Vosse, S.J., van Wieringen, W.N., Wilting, S.M., and Ylstra, B. (2007). CGHcall: calling aberrations for array CGH tumor profiles. Bioinforma. Oxf. Engl. *23*, 892–894.

Wolff, A.C., Hammond, M.E.H., Schwartz, J.N., Hagerty, K.L., Allred, D.C., Cote, R.J., Dowsett, M., Fitzgibbons, P.L., Hanna, W.M., Langer, A., et al. (2007). American Society of Clinical Oncology/College of American Pathologists guideline recommendations for human epidermal growth factor receptor 2 testing in breast cancer. J. Clin. Oncol. Off. J. Am. Soc. Clin. Oncol. *25*, 118–145.
