# Supplementary material for: Principles Governing A-to-I RNA Editing in the Breast Cancer Transcriptome
Source: Cell Rep. 2015 Oct 1;13(2):277–89. doi: 10.1016/j.celrep.2015.09.032 (PMC5326813; doi:10.1016/j.celrep.2015.09.032)
Supplement: Document S2. Article plus Supplemental Information [file mmc9.pdf]

Cell Reports

Article

Principles Governing A-to-I RNA Editing in the Breast Cancer Transcriptome

Graphical Abstract

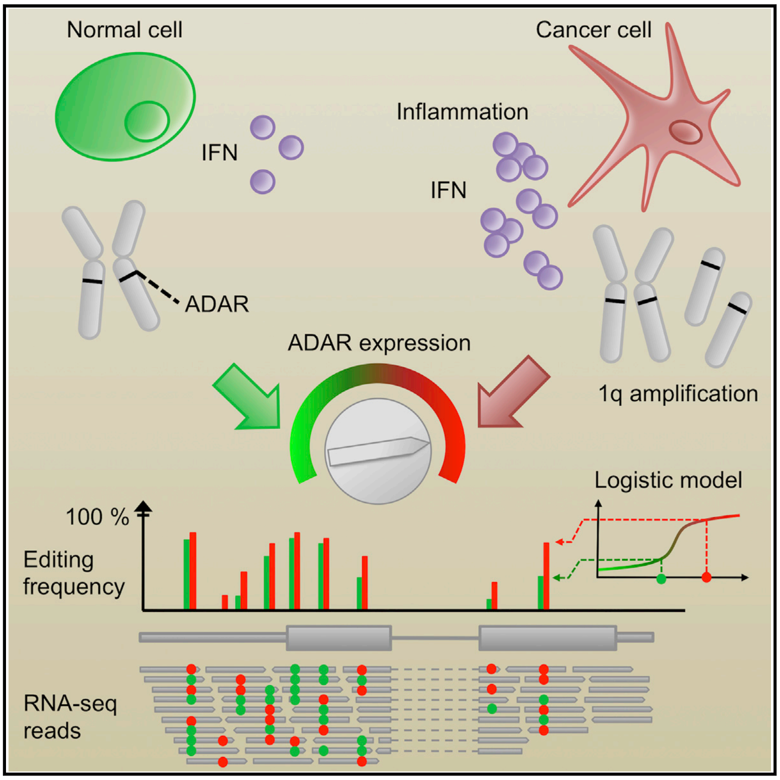

Authors

Debora Fumagalli, David Gacquer, Françoise Rothé, ..., Peter J. Campbell, Christos Sotiriou, Vincent Detours

Correspondence

christos.sotiriou@bordet.be (C.S.), vdetours@ulb.ac.be (V.D.)

In Brief

Fumagalli et al. identify the principles governing A-to-I editing in breast and potentially all types of cancer, demonstrating that A-to-I editing is a pervasive source of transcriptome variation that is mainly controlled by two factors, 1q amplification and inflammation, both of which are highly prevalent among human cancers.

Highlights

- A-to-I editing is a major source of mRNA variability in breast and other cancers
- RNA editing is globally controlled by tumor interferon and ADAR copy number
- Both these factors are highly prevalent among human cancers
- RNA editing sites might represent a new class of therapeutic targets

Accession Numbers

GSE43358

Fumagalli et al., 2015, Cell Reports *13*, 277–289
October 13, 2015 ©2015 The Authors
http://dx.doi.org/10.1016/j.celrep.2015.09.032

Cell Reports
Article

OPEN ACCESS
CellPress

Principles Governing A-to-I RNA Editing in the Breast Cancer Transcriptome

Debora Fumagalli,[1,10] David Gacquer,[2,10] Françoise Rothé,[1,10] Anne Lefort,[2] Frederick Libert,[2] David Brown,[1] Naima Kheddoumi,[1] Adam Shlien,[4] Tomasz Konopka,[2] Roberto Salgado,[1] Denis Larsimont,[5] Kornelia Polyak,[6] Karen Willard-Gallo,[7] Christine Desmedt,[1] Martine Piccart,[8] Marc Abramowicz,[9] Peter J. Campbell,[4] Christos Sotiriou,[1,8,11,*] and Vincent Detours[2,3,11,*]

[1]Breast Cancer Translational Research Laboratory, Jules Bordet Institute, Université Libre de Bruxelles (ULB), Boulevard de Waterloo, 125-1000 Brussels, Belgium
[2]IRIBHM, Université Libre de Bruxelles (ULB), Route de Lennik, 808-1070 Brussels, Belgium
[3]WELBIO, Route de Lennik, 808-1070 Brussels, Belgium
[4]Cancer Genome Project, Wellcome Trust Sanger Institute, Wellcome Trust Genome Campus, Hinxton, Cambridgeshire CB10 1SA, UK
[5]Department of Pathology, Jules Bordet Institute, Université Libre de Bruxelles (ULB), Boulevard de Waterloo, 125-1000 Brussels, Belgium
[6]Department of Medical Oncology, Dana-Farber Cancer Institute, 450 Brookline Avenue, Boston, MA 02215, USA
[7]Molecular Immunology Unit, Jules Bordet Institute, Université Libre de Bruxelles (ULB), Boulevard de Waterloo, 125-1000 Brussels, Belgium
[8]Department of Medicine, Jules Bordet Institute, Université Libre de Bruxelles (ULB), Boulevard de Waterloo, 125-1000 Brussels, Belgium
[9]Department of Genetics, Hôpital Erasme, Route de Lennik, 808-1070 Brussels, Belgium
[10]Co-first author
[11]Co-senior author
*Correspondence: christos.sotiriou@bordet.be (C.S.), vdetours@ulb.ac.be (V.D.)
http://dx.doi.org/10.1016/j.celrep.2015.09.032
This is an open access article under the CC BY license (http://creativecommons.org/licenses/by/4.0/).

SUMMARY

Little is known about how RNA editing operates in cancer. Transcriptome analysis of 68 normal and cancerous breast tissues revealed that the editing enzyme ADAR acts uniformly, on the same loci, across tissues. In controlled ADAR expression experiments, the editing frequency increased at all loci with ADAR expression levels according to the logistic model. Loci-specific "editabilities," i.e., propensities to be edited by ADAR, were quantifiable by fitting the logistic function to dose-response data. The editing frequency was increased in tumor cells in comparison to normal controls. Type I interferon response and *ADAR* DNA copy number together explained 53% of ADAR expression variance in breast cancers. *ADAR* silencing using small hairpin RNA lentivirus transduction in breast cancer cell lines led to less cell proliferation and more apoptosis. A-to-I editing is a pervasive, yet reproducible, source of variation that is globally controlled by 1q amplification and inflammation, both of which are highly prevalent among human cancers.

INTRODUCTION

Although intense effort is currently being dedicated to cancer genome sequencing, comparatively little attention has been devoted at understanding how faithful RNA sequences are to the DNA sequences from which they were derived. mRNA is the target of a series of post-transcriptional modifications that can affect its structure and stability, one of the most relevant being RNA editing (Bass, 2002; Levanon et al., 2004; Nishikura, 2010). The most common form of RNA editing in humans, the A-to-I type, is catalyzed by the adenosine deaminases that act on RNA (ADARs) family of enzymes, which bind double-stranded RNA (dsRNA) and turn adenosines into inosines at precise positions (Bass, 2002; Nishikura, 2010). Inosines are subsequently interpreted as guanosines by the cellular transcription machinery. ADAR enzymes are essential in mammals (Higuchi et al., 2000; Wang et al., 2000) and exist in three forms: ADAR (also known as ADAR1), which is ubiquitous and has two isoforms—p110 is constitutive and p150 is inducible; ADARB1 (also known as ADAR2), principally expressed in the brain; and ADARB2 (also known as ADAR3), which contrary to ADAR and ADARB1 seems to be enzymatically inactive (Chen et al., 2000; Savva et al., 2012).

A-to-I edits can profoundly influence cellular functions and regulations by altering mRNA splicing, stability, localization, and translation, and by interfering with the binding of regulatory RNAs (Athanasiadis et al., 2004; Rueter et al., 1999; Wang et al., 2013). In addition to mRNA, ADAR can target non-coding RNAs such as micro-RNAs (miRNAs), small-interfering RNAs (siRNAs), and long non-coding RNAs (lncRNAs), affecting both their structure and activities (Blow et al., 2006; Hundley and Bass, 2010; Kapusta et al., 2013; Kawahara et al., 2007). A-to-I editing has been shown to occur predominantly in highly repetitive *Alu* sequences, likely because their frequency (>10^6) in the human genome makes their arrangement in quasi-palindrome configurations prone to RNA duplex formation highly probable (Athanasiadis et al., 2004; Bazak et al., 2014a; Kim et al., 2004; Levanon et al., 2004). High-throughput sequencing studies suggest that tens of thousands to millions of positions are targeted by A-to-I editing in the human transcriptome (Bahn et al., 2012; Ju et al.,

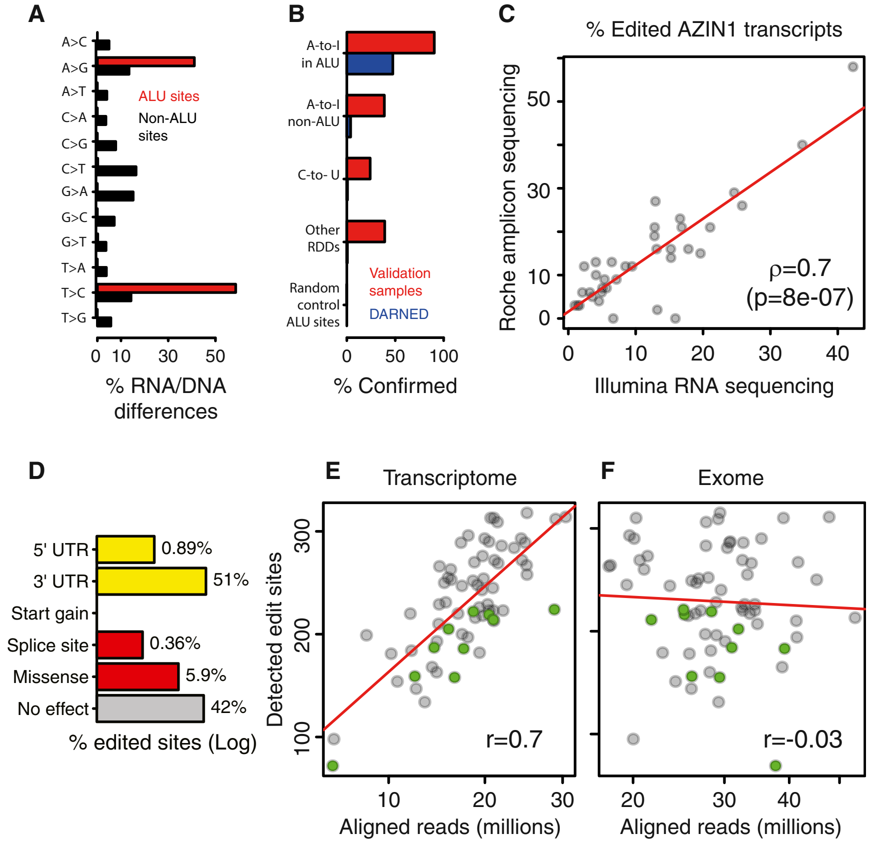

Figure 1. Detection of A-to-I Editing

(A) Substitution frequencies of RDDs.

(B) Percentage of RDDs confirmed in the validation data set, n = 15 BCs (in red), and the DARNED database (in blue). The negative control set is composed of 1,000 sites selected at random positions in randomly selected *Alu* regions. Sites in immunoglobulin (Ig) hyper-variable regions were excluded; see the Supplemental Experimental Procedures.

(C) Each dot represents a sample for which the frequency of edited AZIN1 transcripts has been measured with Illumina full transcriptome sequencing (x axis) and Roche FLX amplicon sequencing (y axis). ρ denotes the Spearman's correlation.

(D) Distribution of the 560 edited sites into functional categories.

(E and F) Number of detected *Alu* A-to-I sites as a function of transcriptome and exome coverages, respectively. Green dots represent tumor-matched normal samples.

2011; Li et al., 2009; Park et al., 2012; Peng et al., 2012; Ramaswami et al., 2012, 2013), and a recent publication reports that potentially all adenosines in specific *Alu* repeats undergo A-to-I editing (Bazak et al., 2014b).

Currently, a limited number of studies on A-to-I RNA editing in cancer have been published, with the findings pointing to a diversity of effects. For example, in brain cancer, editing inhibits cell growth and is reduced in glioma (Maas et al., 2001; Paz et al., 2007) and pediatric astrocytoma (Cenci et al., 2008). In contrast, A-to-I editing increases during chronic myeloid leukemia progression (Jiang et al., 2013). In hepatocellular carcinoma, A-to-I editing of the antizyme inhibitor 1 (*AZIN1*) increases and neutralizes a key inhibitor of the polyamine synthesis pathway, thereby promoting proliferation in vitro and increasing tumor initiation and volume in a mouse xenograft model (Chen et al., 2013). The studies published so far included a small number of samples—an important limit given the sheer diversity of tumor transcriptomes—and/or investigated a limited number of editing sites. Whether the edited transcripts originated from cancer cells or other cell types, e.g., immune cells, present in the tumor mass was not addressed. Hence, both the magnitude and mechanisms regulating A-to-I editing in the majority of cancers, including breast cancer (BC), remain largely unknown.

The main objective of this study was to investigate the principles governing the A-to-I editing process in BC as well as in other types of cancer.

RESULTS

Detection and Validation of A-to-I Editing Sites in Breast Tissue

The extent of A-to-I RNA editing in BC was investigated by paired exome and transcriptome sequencing of a broad series of BC samples representing the principal intrinsic subtypes including 17 triple-negative (TN), 14 HER2-positive (HER2), 16 luminal A (LA), and 11 luminal B (LB) tumors (Table S1). Paired exome and transcriptome sequencing of matched, tumor-adjacent normal tissue was performed on ten cases from this series. RNA-DNA single nucleotide differences (RDDs) were called as outlined in Figure S1 (details in the Supplemental Experimental Procedures).

Overall, we detected 16,027 RDDs in one or more samples, with all possible base changes represented (Figure 1A). Among these, 560 RDDs were located in *Alu* regions, and all were of the A-to-I type (Figure 1A; Table S2), consistent with the notion that A-to-I editing occurs predominantly in forward-facing *Alu* forming dsRNA duplexes processed by ADAR. Forty-seven percent of the A-to-I *Alu* RDDs were present in the DARNED RNA editing site database (Kiran and Baranov, 2010). In contrast, only 2.5% of A-to-I, non-*Alu* RDDs and 0.6% of non A-to-I RDDs were found in the DARNED database (Figure 1B).

Breast tissue is not well represented in the studies covered by the DARNED database. Given that gene expression and RNA editing frequency (defined for each sample as the ratio of the number of RNA sequencing (RNA-seq) reads documenting the non-reference base relative to the total number of reads covering the site) could be regulated in a tissue specific manner, we further validated our findings in an independent breast series. This independent validation series included 15 BC samples with paired transcriptome and full genome sequencing data from the Sanger Institute. The genomic coordinates of our

putative RDDs and the coordinates of 1,000 random *Alu* positions were sent to the Sanger Institute without any additional information. This blind test—based on an independent RDD detection pipeline (Supplemental Experimental Procedures)—confirmed 90% of the *Alu* RDDs, while only one of the 1,000 random *Alu* sites was detected in the validation series. Beyond *Alu*, overlap with the validation series was below 40% (Figure 1B). Given the low confirmation rate of RDDs located outside of *Alu* regions in both the DARNED database and the independent validation series, and that the majority of human editing events are A-to-I detected in *Alu* repeats (Athanasiadis et al., 2004; Bazak et al., 2014b; Kim et al., 2004; Levanon et al., 2004), our subsequent analyses focused exclusively on the subset of A-to-I RDDs located in *Alu* sequences. Since several works have reported the editing of *AZIN1*, this target was also included in our analyses (Chen et al., 2013; Ju et al., 2011; Li et al., 2009, 2011; Peng et al., 2012; Qin et al., 2014; Ramaswami et al., 2012; Shah et al., 2009).

To evaluate the accuracy of edited transcript frequencies measured in our full transcriptome data, we generated amplicons of the *AZIN1* editing site region for 36 samples that were then analyzed by an independent sequencing technology (Roche FLX sequencer). The edit frequencies measured from full transcriptome and amplicon sequencing were remarkably consistent (Figure 1C) and thereby validated the accuracy of these estimations.

The distribution of A-to-I within *Alu* edits according to functional effect is shown in Figure 1D; functional information for all putative and confirmed edited sites is available Table S2.

The Apparent Size of the Editome Depends on the Transcriptome Sequencing Depth and on the Span of Sequenced Genomic Regions

Sequencing depth is a key factor in detecting single nucleotide variations (Bazak et al., 2014b), leading us to ask whether the exome and RNA sequencing depths could influence the number of detectable *Alu* edit sites. While this number was not dependent on the exome sequencing depth, it did greatly increase with the transcriptome coverage (Figures 1E and 1F; Table S3). No plateau was reached in our data set, which had a maximum coverage of $\sim 3 \times 10^7$ reads/sample. This suggests that with higher transcriptome coverage additional A-to-I editing sites should be detectable in the breast transcriptome.

A comparison of our results and methods with previous literature is presented in Tables S4A and S4B. This analysis revealed that genome sequencing span is among the main factors limiting the RDD detection. Since our DNA sequencing covered the exome and not the entire genome, we implemented a less conservative editing detection pipeline bypassing the exome DNA comparison and focusing instead the detection of A-to-I editing on sites previously reported in the literature (Supplemental Experimental Procedures). This DNA-free pipeline detected 59,993 A-to-I editing sites. The main variable investigated in this paper, namely, the mean editing frequency, estimated from these 59,993 sites or the 560 *Alu* sites obtained with the DNA-based pipeline, was nearly identical ($\rho = 0.9$, $p = 2 \times 10^{-16}$). Most of the sites detected by the DNA-free pipeline were expressed in few samples (median, 14.7% of the samples; interquartile range [IQR], 4.4%–50%) and/or edited at low frequency (median, 0% of the reads; IQR, 0%–3.4%); i.e., they were of limited interest as far as correlative analysis across a significant fraction of the cohort is concerned and most probably had negligible influence on cancer progression. The number of sites dropped from 59,993 to 1,852 after filtering out positions expressed at detectable levels in <75% of the samples and not edited at a frequency >10% in any samples. By contrast, applying the same filter to the DNA-based pipeline reduced the number of sites from 560 to 455.

More A-to-I Editing Was Found in Tumor Compared to Normal Matched Breast Tissue

To determine whether A-to-I editing is specifically altered in BC, the mean editing frequencies across all edited sites were compared between matched normal and tumor breast tissues for ten cases where paired exome and transcriptome sequencing data were available for the normal tissue. We also compared the specific edit frequency of the *AZIN1* transcript determined by high-depth amplicon sequencing (Roche FLX sequencer) between tumor and matched normal breast tissues. The global mean editing frequency and the *AZIN1* specific editing frequency were higher in tumor compared to matched-normal breast tissues (Figures 2A and 2B; Tables S3 and S5).

Normal breast samples may contain less epithelial cells; hence, lower editing in these samples could be a trivial consequence of lower editing in non-epithelial cells (e.g., adipocytes) compared to epithelial cells. Thus, the site-averaged editing frequencies across all 560 *Alu* sites from the independent validation series (15 BCs) were compared to eight normal breast organoids (i.e., freshly isolated uncultured intact breast milk ducts). Editing was higher in tumor compared to pure normal epithelial cells (Figure 2C), which validates our findings.

Global A-to-I Editing Is Governed by ADAR Expression and Site-Specific Editability

The general principles governing A-to-I editing in BC were investigated in multiple, matched exome-transcriptome data pairs. The ADAR family of enzymes catalyzes A-to-I editing, leading us to first determine their expression levels in normal and tumor breast tissues as well as their association with editing frequency using transcriptome sequencing data. ADAR was expressed 9-fold more than ADARB1 and >1,000-fold more than ADARB2 ($p < 10^{-16}$, Figure S2), which was anticipated because these last two isoforms are principally expressed in the brain. Moreover, while ADAR expression was higher in tumor compared to patient-matched normal breast tissues ($p = 0.005$, Figure S2), an inverse borderline-significant trend was observed for ADARB1 ($p = 0.1$, Figure S2).

The mean editing frequency (defined as the average editing frequency of all 560 *Alu* sites) was significantly positively correlated with ADAR mRNA expression levels (Spearman's $\rho = 0.7$, $p < 2 \times 10^{-16}$; 40% of variance explained; Figure 3A; Table S3), while it was weakly anti-correlated with ADARB1 expression levels (Figure S2), as previously reported (Chen et al., 2013). The global association detected between *ADAR* mRNA expression and the mean editing frequency was also observed at individual editing sites (Figure S2; Table S2). Considering both the high

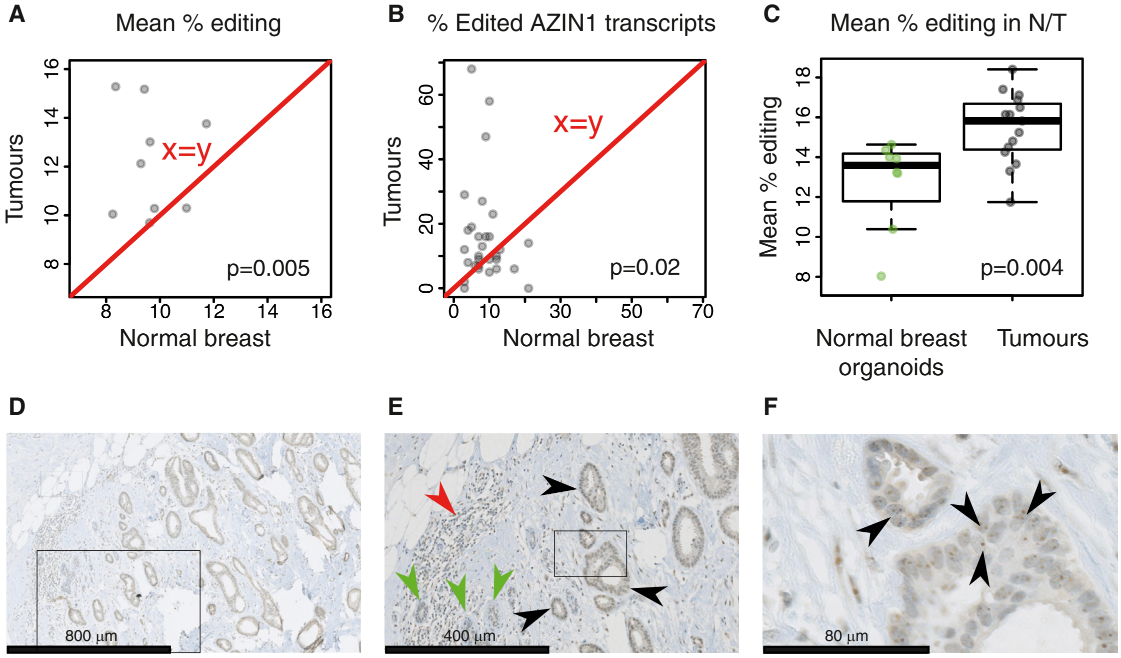

Figure 2. A-to-I Editing and ADAR Expression in Normal and Tumor Breast Tissue
(A) Each dot represents a patient with the mean editing frequency in her normal (x axis) and her matched tumor breast tissue (y axis).
(B) Same as (A), except that the AZIN1 editing frequency measured by Roche FLX amplicon sequencing is depicted.
(C) The mean editing frequency of eight breast organoid cultures is compared to that of 15 breast tumors.
(D) Representative ADAR staining of a luminal A tumor.
(E) Zooming in (D) reveals that tumor staining (black arrows) is higher than in normal epithelium (green arrows) and lymphocytes (red arrows).
(F) Zooming further in (E) reveals a higher staining of nucleoli (black arrows).

levels of *ADAR* mRNA expression and its strong correlation with the mean editing frequency, our further analyses were focused on ADAR.

Editing site distribution across normal and BC tissues was investigated by plotting the maximum edit frequency for all editing sites against the number of samples where editing of these sites was detected (Figure 3B). These two variables were highly correlated indicating that if a site was highly edited in one sample, it was very likely to be edited in many other samples. This also suggested that the editing sites detected in normal tissues are also detected in matched tumor tissues and across all BC patients.

Sites and samples were then ordered by increasing mean editing frequencies, and the individual editing frequencies at all 560 *Alu* sites in all samples were displayed as a heatmap (Figure 3C; negative controls in Figure S2). This revealed that high editing frequencies were present in the samples with more editing sites and high ADAR expression. Conversely, samples with lower ADAR expression had fewer edited sites, which were edited at lower frequencies. Taken together, these data suggest a quantitative model of A-to-I editing (Figure 3D). In this model, turning up the ADAR expression “knob” leads to detectable editing at more sites and an increased editing frequency of all the editable sites. Conversely, when ADAR expression is low, editing is detectable at fewer sites and at a lower frequency. We propose that “editability,” the propensity of a position to be edited by ADAR, depends mostly upon biophysical interactions between an individual site with its surrounding RNA sequence and partnering as a duplex with ADAR. We show below how to quantitatively estimate it from dose-response data.

Validation of the A-to-I Editing Model

We challenged this A-to-I editing model by inducing *ADAR* expression in four breast cell lines (three tumor and one normal tissue derived cell lines) with interferon α, a known ADAR inducer (Patterson and Samuel, 1995). The effect of inducing *ADAR* overexpression on the editing frequency of *AZIN1* and four of the most edited *Alu* regions in the discovery series was analyzed by amplicon sequencing (Roche FLX sequencer). These experiments demonstrated: First, that the same sites were edited in all cell lines (Figure S3; Table S6), including 90 of the 91 sites detected by whole-transcriptome sequencing in vivo. Second, that the editing frequency profiles were similar across all cell lines (Figure S3). Third, that *ADAR* induction increased editing frequencies at all edited positions (Figures 4A and S3). Fourth, that *ADAR* induction and/or increase of depth of coverage increased the number of detected editing sites (Figure 4B). Due to deeper coverage (typically >1,000× for the Roche FLX sequencer) of the cell line amplicons, we identified 137 new sites in addition to the 90 in the discovery data set, which suggests there are likely more sites to identify in breast tissue.

We took advantage of the long reads (>300 bp) and high coverage of the Roche FLX data to further validate our model by applying it to thousands of individual mRNA molecules

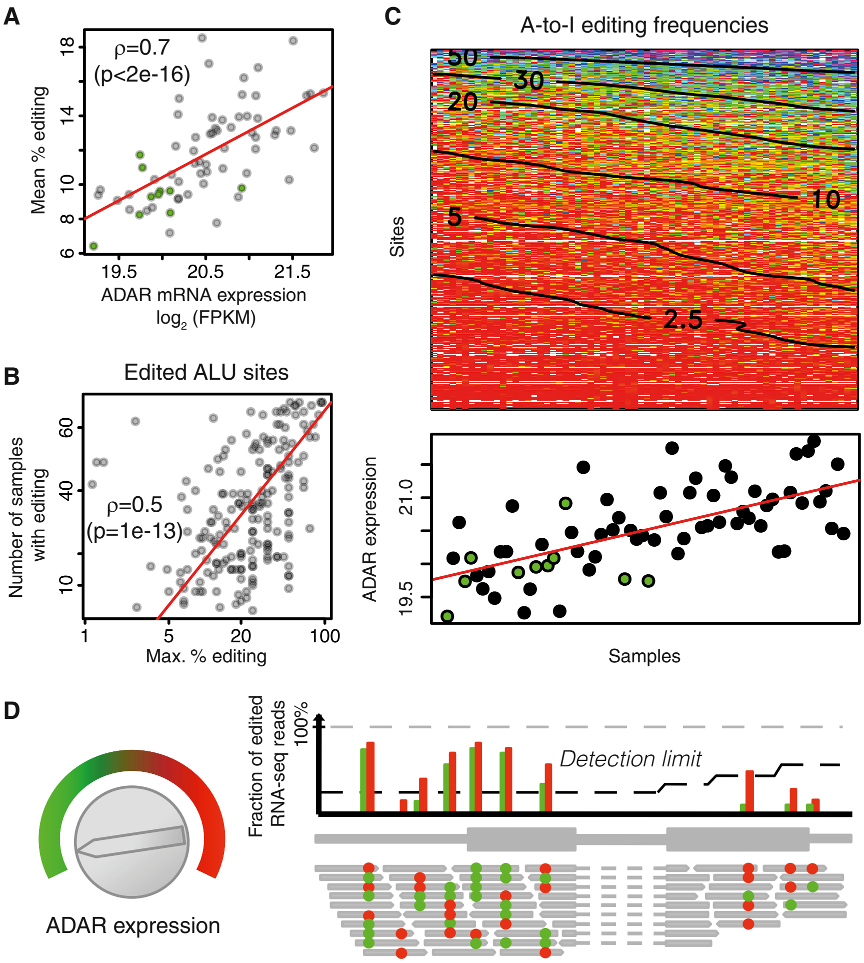

Figure 3. Model of A-to-I Editing

(A) Each dot represents a sample with its RNA-seq-estimated ADAR expression on the x axis (in $\log_2$ of fragments per kilobase per million mapped reads), and its mean editing frequency across all 560 *Alu* sites on the y axis. Green dots represent tumor-matched normal samples. The RNA-seq expression of *ADAR* is highly correlated with microarrays and qRT-PCR expression (Figure S2).

(B) Each dot represents an *Alu* A-to-I editing site with the maximal edit frequency across all samples on the x axis and the number of samples in which it was detectably edited on the y axis.

(C) Heatmap of editing frequencies across all *Alu* A-to-I edit sites in all samples. Both are ordered by increasing (down-to-up, left-to-right) mean editing frequencies. Smoothed contour lines labels give the percentage of edited transcripts. The bottom panel shows corresponding ADAR expression. Green dots represent tumor-matched normal samples. Negative controls are presented in Figure S2.

(D) Model of A-to-I editing. Turning the ADAR "expression knob" clockwise increases ADAR expression. As a result, more transcripts are edited (red dots), and the editing frequency of all editable sites increases accordingly (compare green versus red bars). Moreover, the detection limit at some sites for which editing was previously undetectable is passed. The detection limit depends on sequencing coverage, which is lower on the right-most exon. Importantly, the ranking of editing frequencies of the different sites is unaltered by *ADAR* expression.

transcribed from the same DNA region in the same individual. Focusing on one 256-bp *Alu* region in one cell line, 65 of 68 adenosines potentially targeted by ADAR (Figure 4C) were edited in at least one of the 2,842 mRNA molecules analyzed. The number of edited positions per transcript was highly variable, ranging from 0 to 26 (38% of all adenosines). As expected, the sets of edited positions in "low-edited" mRNA molecules tended to be subsets of those edited in "high-edited" mRNA molecules. These findings further validate our A-to-I editing model. Nevertheless, the editing process had a strong stochastic component at the level of individual molecules. This is at odds with the deterministic nature of editability, a quantity defined at the level of populations of RNA transcripts. We propose to reconcile these two viewpoints by interpreting editability as a probability of edition by ADAR.

Quantitative Estimation of Site-Specific Editability with the Logistic Model

The dependence of site-specific editing frequencies on ADAR protein expression in our in vitro experiments is shown in Figure 4D. Editing frequencies increase monotonously with ADAR until a site-specific saturation threshold is reached. This suggests that these frequencies could be approximated with the logistic model, $f(x) = \varepsilon_i/(1+\exp(\omega_i - x))$, at each site i. The offset of the s-shaped curve is controlled by ω_i and the editing frequency at saturation by ε_i. We propose ε_i—a quantity independent of ADAR expression—as the mathematical definition of site-specific editability, putting this concept on a firm quantitative ground.

We estimated ε_i and ω_i by fitting the logistic model to each one of the dose-response curves shown in the above graphics. A typical fit is shown in Figure 4E (see also Figure S4) and the distributions of ε_i and ω_i across all sites in Figures 4F and 4G. As expected, ε_i estimates are spread over the entire [0, 1] interval. The ω_i estimates are centered around a unique value, i.e., ω_i is essentially site independent. Related p values (Figure S4) are small considering that only four points were available for each fit. Although saturation was reached for two ADAR expression data points in one experiment but not in the others, the estimates obtained for independent experiments were consistent ($\rho = 0.97$, $p < 2 \times 10^{-16}$; Figure S4). The lower coverage of our in vivo data was not sufficient to adequately fit the logistic model, but ε_i estimated in vitro is highly correlated with the mean editing frequency measured in vivo (Figure 4H). In vivo editing is, on average, well below saturation (Figure 4H). Hence, the logistic model provides an operational procedure to derive useful quantitative estimates of site-specific editability from dose-response data.

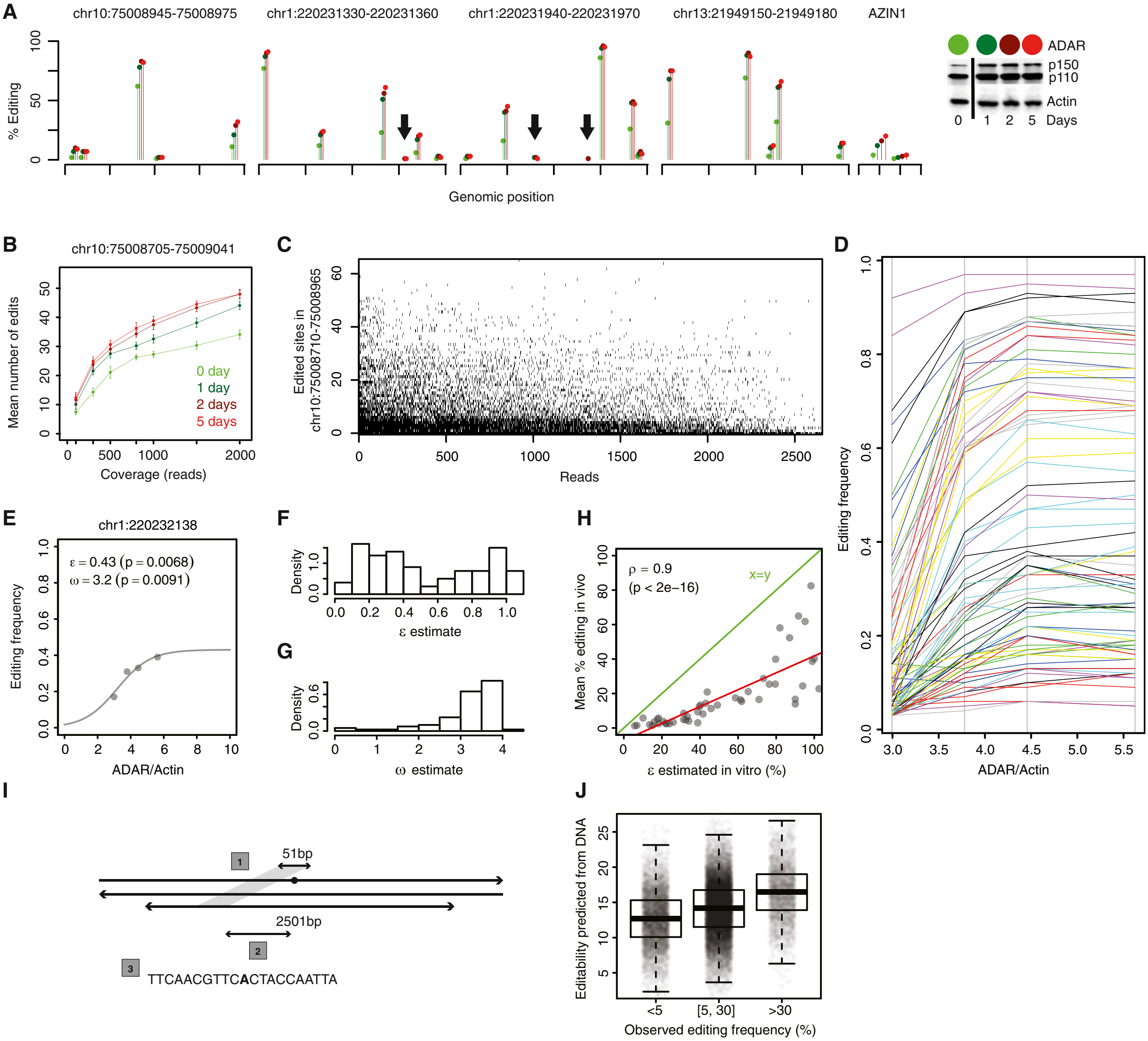

Figure 4. Validation of the A-to-I Editing Model and Quantitative Estimation of Site-Specific Editability

(A) Effect of increasing ADAR expression in the cell line MCF7 on editing in four representative *Alu* regions and AZIN1. The full-length of sequenced regions are shown in Figure S3 for MCF7 and three more cell lines. Complete ADAR western blots quantifications underlying the color scale are provided in Figure 5E (see baseline t = 0 and IFN-α, $t \in \{1, 2, 5\}$ days tracks) and in Figure S7. Increasing ADAR expression increases the editing frequency at all editable positions, as predicted by the model of Figure 3D. Similar results were obtained for IFN-β and IFN-γ (global, position-less, view Figure 5F). Arrows point at editing sites detectable only at higher ADAR expression in our assay.

(B) Increasing sequencing coverage (x axis) or ADAR expression (color scale) increases the number of detectable editing sites (y axis). Coverage variation was implemented by down-sampling the total pool of sequencing reads, starting from 2,000×, down to 100×, and re-running the variant detection pipeline for each down-sampled alignment. Each data point is the mean of 30 down-sampling experiments. Error bars, SD.

(C) Editing of individual mRNA molecules. Each black dot depicts an edited base in a given mRNA molecule. The y axis goes from 0 to 60 and corresponds to the adenosines in the ~250-bp span that are edited in at least one of the 2,842 reads represented along on the x axis. Reads and adenosines were ordered by decreasing editing frequencies. 185 non-edited reads were omitted from the figure.

(D) Dose-response curves for experiment in cell line BT474. ADAR was increased through IFN-α stimulation (as in A). We focused on 81 sites (color lines) with a baseline editing frequency >2.5% in order to avoid trivial nonlinear effects caused by lack of detection at low ADAR expression.

(E) Example of a fit of the logistic model (line) to experimental data points (dots). The unit of ω is commensurate to the dimensionless ADAR relative expression and ε is the fraction of edited transcripts at saturation.

(F and G) Distributions of ε and ω across the 81 sites.

(legend continued on next page)

Site-Specific Editability Is Correlated with Local Sequence Features

We hinted that editability depends upon biophysical interactions between an individual site with its surrounding RNA sequence and partnering as a duplex with *ADAR*. This implies that editability should be partially predictable from the sequence data, so we sought to develop and validate a simple proof-of-principle DNA-based statistical model for editability. The model relies on the notions that (1) an edited site must be part of an RNA duplex, implying that it lies within a sequence with a nearby palindromic match, and (2) ADAR activity depends upon a specific nucleotide sequence in the vicinity of the edited base (Figure 4I; Supplemental Experimental Procedures). To build the model, we analyzed the editing frequencies of 51,621 edited *Alu* sites with $\geq 20\times$ coverage from an independent sample sequenced at very high coverage (Ramaswami et al., 2012). These sites were then ordered by genomic position. The first half was used to fit a statistical model of the edit frequency based on DNA data alone. Editability scores were then computed for the second half of the sites (not used to train the model), which turned out to be strongly associated with the observed editing frequencies (Figure 4J). Our validated statistical model supports the notion that the editability of a given site is partly determined by the local site-specific DNA features. Of note, the logistic fit of dose-response data, not the DNA-based model, should be used to estimate quantitatively editability.

Association of ADAR Expression, A-to-I Editing, and Clinico-Pathological Variables

The relevance of ADAR expression to the A-to-I editing process led us to analyze its tissue and cellular localization by immunohistochemistry (IHC). Uniform ADAR expression was detected in cancer cells (Figures 2D–2F) but to a lesser extent in normal cells and tumor-infiltrating lymphocytes (TILs; see Figure 2E). Moreover, ADAR staining was markedly stronger in nucleoli (Figure 2F), in agreement with previous findings (Desterro et al., 2003; Sansam et al., 2003).

To investigate the potential clinical impact of A-to-I editing, we determined whether the mean editing frequency was associated with the tumor cell content (i.e., the proportion of malignant epithelial cells, adipose, stroma, normal epithelial cells and TILs) and/or well-established clinico-pathological parameters, including estrogen receptor, progesterone receptor, the proliferation marker Ki67, HER2 status, tumor size, nodal status, and histological grade. The mean editing frequency was positively correlated with the percentage of TILs (Spearman's correlation $\rho = 0.3$, $p = 0.02$), tumor size ($\rho = 0.3$, $p = 0.01$), and HER2 IHC staining ($\rho = 0.3$, $p = 0.01$; Figure S5; Tables S1 and S3). Multivariate analysis of this data set suggests that TILs and HER2 IHC are dependent variables in their association with editing frequency (Figure S5).

To circumvent our limited sample size, correlations between these variables and ADAR expression were assessed in a large cohort of 787 BC patients with HER2 analyzed by IHC (Curtis et al., 2012). TILs were not scored in this series so the level of Signal Transducer and Activator of Transcription 1 (*STAT1*) expression, a proxy for type I interferon response, was used instead. This independent BC series confirmed an association between ADAR and STAT1 expression but not for HER2 status or tumor size (Figure S5). The lack of an association with estrogen receptor, Ki67, and HER2 indicates that ADAR expression is not correlated with a specific BC subtype beyond their link with the adaptive immune response.

The Interferon Response and Gains in *ADAR* Copy Number Independently Control A-to-I Editing in Cancer

The biological processes potentially associated with RNA editing were investigated by searching for genes whose expression had a strong positive correlation with the mean editing frequency (details in the Supplemental Experimental Procedures). Remarkably, 62 of the 85 genes identified were located on chromosome 1q ($p = 10^{-66}$). Since *ADAR* is located on chromosome 1q, we next used SNP array data to determine *ADAR* copy numbers in our samples. *ADAR* amplification was frequent in our series (44%) and correlated with high mean editing frequencies (Figure 5A).

Chromosome 1q contains hundreds of genes and therefore its amplification could have a systemic impact on the BC transcriptome (Curtis et al., 2012). Therefore, we further characterized the genes correlated with editing that were independent from 1q amplification. First, the microarray expression data were adjusted for 1q copy number to remove any potential confounding effects of *ADAR* amplification, and then gene set analysis was performed (Efron and Tibshirani, 2007) to identify canonical pathways associated with the mean editing frequency. The 13 significant pathway gene sets revealed by this analysis were all involved in interferon responses, interferon-related DNA and RNA sensing, and lymphocyte biology (Figure S6). We also investigated gene sets with shared transcription factor binding motifs between their promoters. The seven significant gene sets identified were overwhelmingly related to *NFκB* and the interferon response, including the Interferon Response Factors *IRF1*, *IRF2*, and *IRF7* (Figure S6). To further investigate the relationship between interferon-related genes and ADAR expression, the median expression levels of STAT1 (Figure 5B) and 389 type I interferon-inducible genes (Figure S6) derived from ten microarray studies (Schoggins et al., 2011) were measured. The expression of STAT1 and the 389 genes were positively

(H) The 81 edited sites are depicted as dots with the corresponding ε_i estimates derived from the BT474 cell lines on the x axis and their in vivo editing frequency on the y axis.

(D)–(H) are part of a more comprehensive analysis presented in Figure S4.

(I) DNA-based statistical model of editability. The model included three parameters: (1) the best Smith-Waterman global alignment score of the 51-bp sequence surrounding the editing site (green dot) within the 2,501-bp sequence surrounding the editing site on the reverse strand; (2) the distance separating the editing site from this best alignment; (3) the 20 nucleotides surrounding the editing site. These 1 + 1 + 20 = 22 variables were fitted with a linear model against the editing frequencies of half of 51,621 *Alu* editing sites with coverage $\geq 20\times$ previously identified (Ramaswami et al., 2012).

(J) Observed editing frequencies versus editabilities predicted from DNA for validation sites.

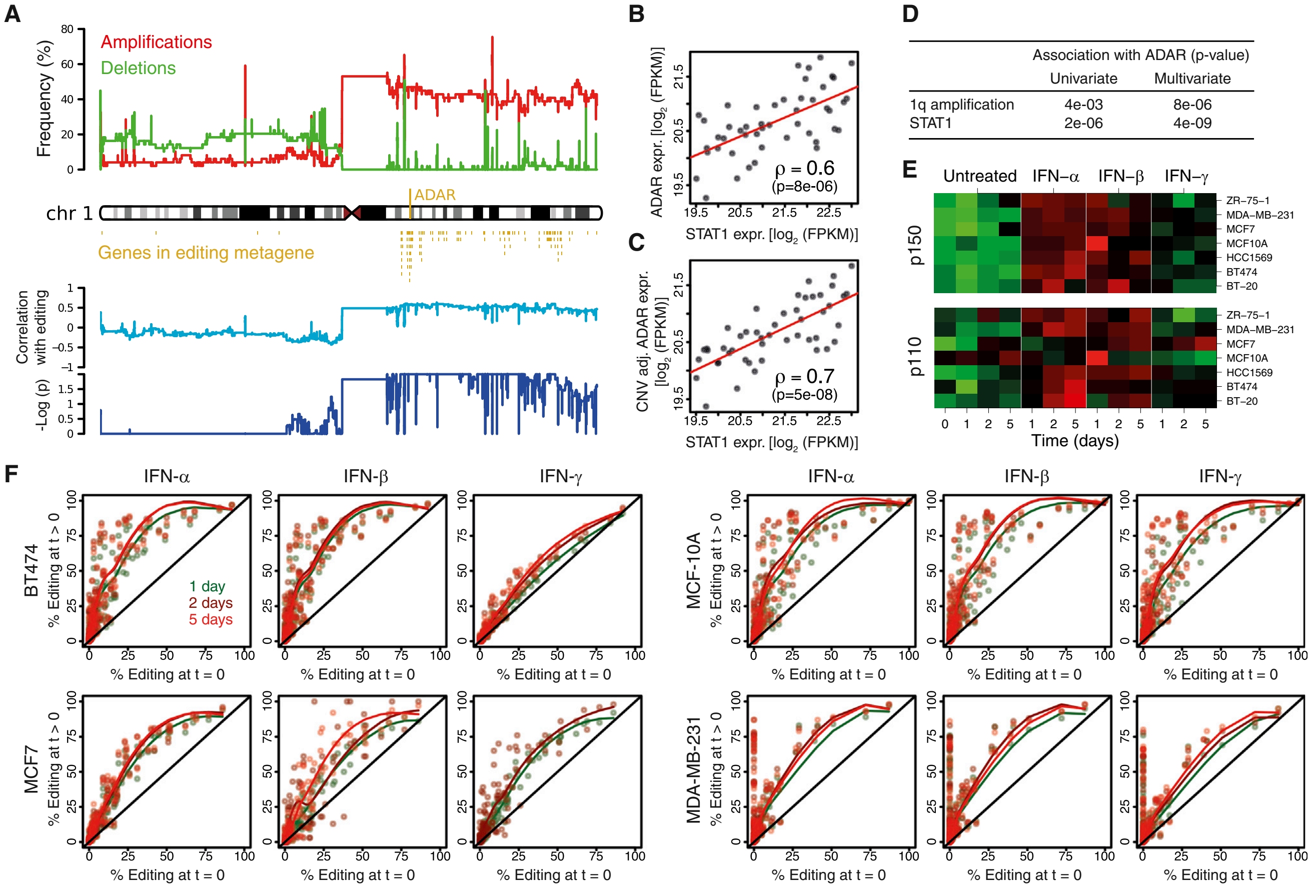

Figure 5. *ADAR* Amplification and the Interferon Response Are Independent Predictors of ADAR Expression in Cancer

(A) The top panel shows the frequencies of amplifications/deletions along chromosome 1 in our series. The middle panel shows the genes whose expression is highly associated with that of ADAR. Nineteen genes not located on chr1 are omitted. The bottom panel shows the Spearman's correlation coefficient and associated p values of non-segmented copy-number array probes with the sample-wise mean editing frequencies.

(B) Dots represent tumor samples, with STAT1 expression on the x axis and ADAR expression on the y axis.

(C) Same as (B) with *ADAR* expression adjusted for *ADAR* copy number.

(D) Association p values of *ADAR* copy number and STAT1 expression with ADAR expression increase in a multivariate analysis, demonstrating that ADAR expression is independently associated with these two variables.

(E) Seven breast cancer cell lines were exposed to interferon α, β, and γ for 1, 2, and 5 days. Western blots quantifications are depicted for each cell line, interferon, and time. Because expression dynamic ranges vary among cell lines, each line has its own color scale extending from low expression in green to high expression in red. The underlying gels are presented in Figure S7 and blot quantification in Table S6. Corresponding mRNA RT-PCR expression data are shown Figure S7 and detailed Table S7.

(F) Editing frequencies in the absence of treatment (x axis) versus interferon treatment (y axis). Points depict the editing sites in AZIN1 and the four *Alu* regions of Figure S3. Points are above the identity line x = y (black diagonals); i.e., interferons increase editing frequencies at all sites. Library preparation failed for MCF7/IFN-γ at 5 days. Limited sequencing coverage precluded detection of some editing events for MDA-MB-231, t = 0 and t = 1 days.

associated with ADAR expression, suggesting that increased editing was part of a broader type I interferon response related to the chronic inflammatory state in cancer.

The respective roles of *ADAR* copy number and STAT1 expression (as a proxy for interferon response) in the A-to-I editing process were further defined using multivariate analysis to demonstrate that they are independently associated with *ADAR* expression (Figures 5B–5D). STAT1 was correlated with ADAR expression (Figure 5B), and this correlation could be strengthened by adjusting ADAR expression for *ADAR* DNA copy number (Figures 5C and 5D). Taken together, STAT1 and *ADAR* copy number explained 53% of ADAR expression variation. The independent effect of type I interferon response and *ADAR* amplification was also supported by measuring the constitutive p110 and interferon-inducible p150 ADAR isoforms (Figure S6). STAT1 expression was more strongly correlated with p150 than p110, and, conversely, *ADAR* copy number was more strongly correlated with p110 than p150.

While *ADAR* amplification is likely limited to malignant epithelial cells, the type-I interferon effect could be principally mediated by TILs. To further explore this, we treated seven breast cell lines (derived from the four principal BC molecular subtypes and normal breast) with individual interferons (α, β, and γ) to determine whether editing can be directly increased

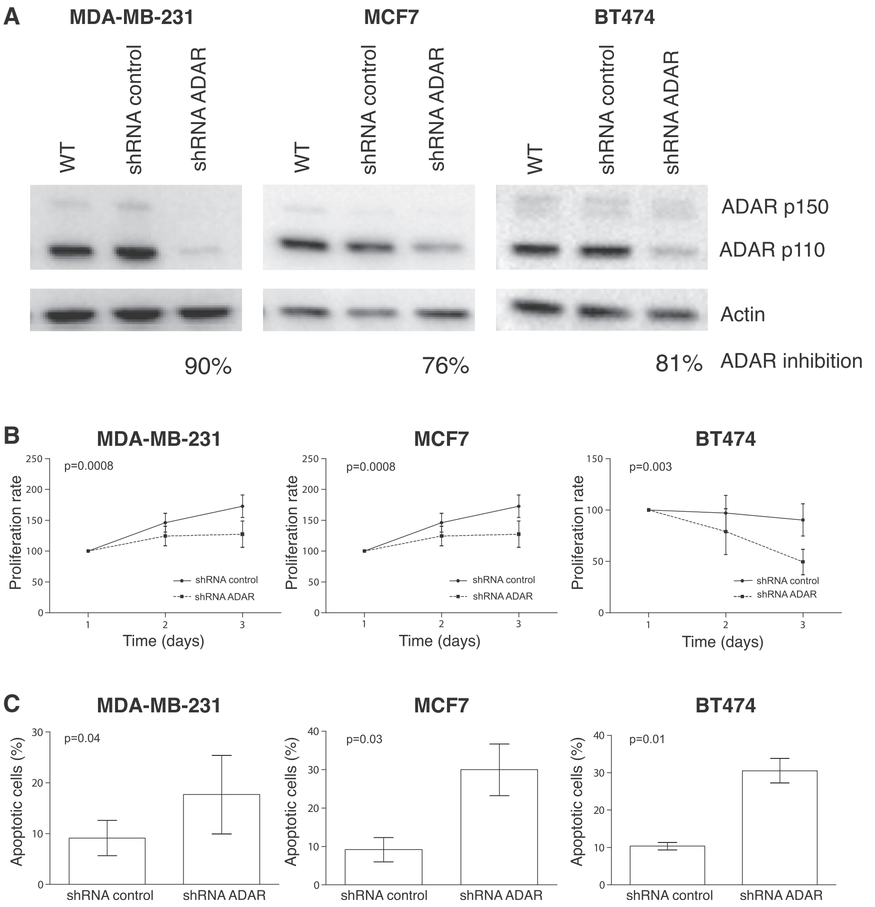

Figure 6. ADAR Involvement in Cell Proliferation and Apoptosis

(A) Western blot analysis of ADAR silencing after shRNA lentiviral transduction in MDA-MB-231, MCF7, and BT474 breast cancer cell lines.

(B) ADAR silencing statistically decreases cell proliferation. Cell growth curves for ADAR-knockdown cells (shRNA ADAR) and control cells (shRNA control) in MDA-MB-231, MCF7, and BT474 BC cell lines.

(C) ADAR silencing statistically increases cell apoptosis. Illustration of the percentage of apoptotic cells in ADAR-knockdown cells (shRNA ADAR) and control cells (shRNA control) in MDA-MB-231, MCF7, and BT474 BC cell lines. Error bars depict SDs of three independent experiments.

by interferon. ADAR p150 protein expression increased with all three interferons in all cell lines at each time point (Figures 5E and S7), while p110 induction was weaker and less consistent. The moderate but significant correlation between p110 and STAT1 mRNA detected in primary tumors suggests that a small amount of p110 was induced (Figure S6). The same four cell lines used to validate our A-to-I editing model were analyzed for p150 and p110 *ADAR* mRNA isoform expression levels, the editing proportion of *AZIN1* and the four most edited *Alu* regions previously selected. The mRNA levels for p110 and p150 isoforms paralleled their protein expression (Figure S7). Moreover, editing increased at all editable sites with all interferons in the four cell lines (Figure 5F). Higher editing levels were observed at 2 or 5 days compared to untreated or 1 day. The induction of ADAR and editing was lowest for IFN-γ. These experiments confirm that type-I interferon response affect A-to-I mRNA editing in epithelial cells.

ADAR Is Involved in Cell Proliferation and Apoptosis in Breast Cancer

Given that we have shown that both ADAR expression and mean editing frequency were higher in breast tumors compared to matched normal tissues, we aimed to further investigate ADAR's role on cell proliferation, migration, and apoptosis. To that purpose, ADAR expression was stably knocked down in three representative BC cell lines (MDA-MB-231, MCF7, and BT474) using small hairpin RNA (shRNA) lentiviral particles (shRNA ADAR). The three cell lines were also transduced with scramble shRNA lentiviral particles (shRNA control) as a negative control for the functional experiments. ADAR silencing was confirmed by western blot analysis (Figure 6A).

To assess the role of ADAR in cell proliferation, MTT assays were performed. These experiments showed that ADAR silencing led to a statistically significant decrease in cell proliferation (shRNA ADAR) compared to the control cells (shRNA control) in all cell lines (Figure 6B). These results suggest that ADAR promotes cell proliferation. No significant effect of ADAR silencing was found on cell migration. The role of ADAR in apoptosis was investigated using Annexin V assays. ADAR silencing led to a statistically significant increase in cell apoptosis (shRNA ADAR) compared to the control cells (shRNA control) in all cell lines (Figure 6C) suggesting that ADAR may act as an anti-apoptotic factor.

The Role of *ADAR* Copy-Number Gains and Interferon Responses in Other Cancers

ADAR amplification is frequent in human cancers (Figure 7) and inflammatory responses are pervasive in this disease. This information led us to investigate whether these two factors were related to ADAR expression in 4,480 cancers from The Cancer Genome Atlas (TCGA, http://cancergenome.nih.gov/) for which sample-matched expression and copy-number profiles were available. The representative analyses shown in Figures 5B and 5C were reproducible across the TCGA data set, which spanned 20 types of cancer from 16 organs (Figure 7). Overall, ADAR expression was consistently associated with both *ADAR* copy number and STAT1 expression. Similar to BC, adjusting ADAR expression for *ADAR* copy number increased the correlation between ADAR and STAT1 for all except pancreatic, kidney, and thyroid tumors. The frequency of *ADAR* amplification was

OPEN ACCESS
CellPress

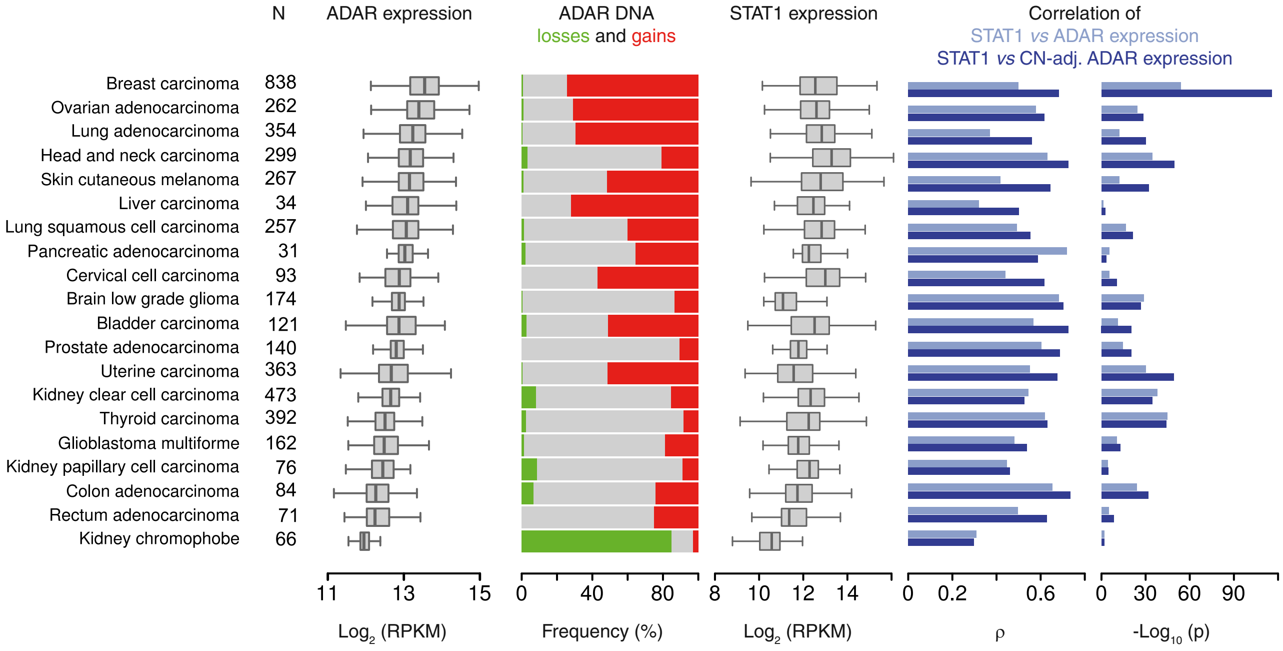

Figure 7. *ADAR* Amplification and the Interferon Response Predict ADAR Expression in Human Cancers

We included all TCGA data sets and tumors (see "N" column) for which both copy-number and RNA-seq expression data (pipeline v.3) were available. Data sets are ordered by decreasing median ADAR expression (top to bottom). The three leftmost plots depict the distributions of ADAR expression, *ADAR* DNA copy number, and STAT1 expression across each data set. The two rightmost bar plots extend to TCGA data the calculation presented for our data in Figures 5B and 5C. In most cancers, adjusting ADAR expression for *ADAR* copy number increases the Spearman correlation, ρ, with STAT1 (cf. the dark blue bars to the light blue bars).

low in kidney and thyroid tumors, therefore correcting for *ADAR* copy number had a limited effect. These data suggest that ADAR expression could be principally driven by interferon in these two types of cancer. In most cancers, however, the editing process is driven by both type I interferon and *ADAR* copy-number amplification. A correlation between *ADAR* copy number and ADAR expression has also been recently reported in esophageal cancer (Qin et al., 2014).

DISCUSSION

The magnitude of A-to-I editing in cancer as well as the mechanisms controlling and regulating the A-to-I editing machinery are currently unknown. To address both points, we performed a survey on RNA editing in cancer by profiling dozens of BCs and matched healthy breast tissues. The sample size of this study opened a window on principles governing A-to-I editing that were previously out of reach. A significant finding from our study was the demonstration that the same sites are edited in normal and tumor breast tissues as well as in several BC cell lines. We further showed that while the editing frequency profiles are correlated across tissues and BC cell lines, the frequency of editing is significantly higher in tumors compared to their matched normal breast tissues. High editing frequencies are detected in samples with high ADAR expression. These data provide the basis for our A-to-I editing model, where increases in ADAR expression increase the editing frequency of all editable positions in the transcriptome. We successfully validated this model in BC cell lines and showed that ADAR control of site-specific editing frequency can be approximated with the logistic model. ADAR's site-specific activity, that we call editability, is partly influenced by the biophysics of interactions between nucleotides in the surrounding RNA sequences and their duplex partnering with ADAR and can be estimated from dose-response experiments. Finally, we showed that ADAR expression is controlled by 1q amplification and inflammation in human cancers.

In our study, longer *ADAR* induction times and/or deeper sequencing coverage increased the number of editing sites detected. Interestingly, no plateau was reached at the depths we investigated, with up to 3×10^7 aligned reads per sample. A previous study made a similar observation using a coverage of up to 5×10^8 mRNA reads/sample, where no plateau was reached despite >140,000 A-to-I sites detected in the *Alu*'s (Ramaswami et al., 2012). A hundred million sites could be edited in humans (Bazak et al., 2014b). Differences in number of edited sites between the cited works and the present study could be due to the cell type analyzed (e.g., lymphoblastoid cell line versus breast tissues/cell lines) and the DNA (e.g., whole-genome versus coding sequences and their neighborhood) and/or RNA sequencing strategies (Table S4). For example, several studies used the GM12878 and the YH (also known as SRA043767) cell lines for which the transcriptomes were sequenced at the outstanding depth of 0.5–1.2 billions reads and compared to the matched whole-genome sequence. In these studies, the number of editing sites, ranging from ∼20,000 to ∼2 million, is

commensurate to the number of callable bases. Conversely, the studies with lower individual transcriptome coverages report less editing sites, like ours (560 sites) and Bahn et al. (2012) (5,965 sites). Bahn et al. had access to the full genome sequence, while we had access only to the coding DNA sequence (CDS) regions and their neighborhood. In addition, the detection pipeline specificity versus sensitivity trade-off may also play a role. Most previous studies used the A > G ratio as a surrogate for error rates; i.e., they assumed that A-to-I is the only significant RNA editing type and that all A > G RDDs are bona fide editing events. The A > G rate in *Alu* regions is 80% in Bazak et al. (2014a, 2014b), 90% in Peng et al. (2012), 96% in Ramaswami et al. (2012), and 100% in our study. Our pipeline is therefore more conservative according to this criterion, and consequently less putative editing sites were detected. It is anticipated that a large number of additional A-to-I editing sites beyond those identified here remain to be discovered in BC. The data presented here clearly demonstrate that A-to-I editing is a pervasive phenomenon in cancer and suggest that it is a major source of mRNA sequence variability in breast and potentially other types of cancer (Paz-Yaacov et al., 2015; Han et al., 2015). Editing has the potential to significantly impact transcriptional regulation and cellular functions in tumor cells. Indeed, our in vitro studies have shown that *ADAR* silencing decreases cell proliferation and promotes apoptosis supporting the potential carcinogenic role of ADAR and consequently A-to-I editing in BC.

Multiple studies are revealing that aberrant expression of ADAR and APOBEC families of enzymes occurs in many human diseases, including cancer. Since the first studies implementing the sequencing technology in humans, ADAR appeared to be one of the highest overexpressed genes in BC, and its recoding potential started to emerge (Shah et al., 2009). More recent works have shown that in breast and other tumor types mutational signatures are associated with APOBEC family proteins (Alexandrov et al., 2013; Nik-Zainal et al., 2012) with evidence that APOBEC-mediated mutagenesis is highly active in human cancers (Burns et al., 2013; Roberts et al., 2013; Swanton et al., 2015; Zhang et al., 2015). Although the relevance of ADARs and RNA editing in cancer just begins to be recognized (Avesson and Barry, 2014; Han et al., 2014; Mo et al., 2014; Salameh et al., 2015; Witkin et al., 2015), the link between A-to-I editing by ADAR and the type I interferon response shown in our study suggests that the cancer immune response can influence ADAR's activity, as shown in other systems. A significant role for ADAR is further supported by our demonstration that its expression is significantly upregulated by *ADAR* copy-number gains in breast (up to 75%) and other cancers (up to 70%). Overall, these data highlight the potential magnitude of A-to-I RNA editing in tumors and thereby the possibility for large-scale clinical implications. RNA editing and/or APOBEC-mediated mutagenesis could shape the immunogenicity of the tumor and thereby directly affect anti- and/or pro-tumor immune responses. RNA editing itself, the processes it regulates and its potential to differentially direct activities in response to the chronic inflammatory tumor microenvironment, may have important implications for clinical progression in breast and other cancers.

The widespread editing we observed, in combination with the conservation of editing sites detected across tissues and patients, suggests there might be clinical and therapeutic implications for a wide range of cancer patients. However, modulation of editing at an individual site is entangled with many processes. The model we established for A-to-I editing implies that modulation of ADAR will also affect all editable sites in expressed transcripts. In addition, ADAR has been shown to influence miRNA processing (Heale et al., 2009; Ota et al., 2013; Shoshan et al., 2015; Tomaselli et al., 2013; Yang et al., 2006), to control mRNA transcript stability (Wang et al., 2013) and to affect several RNA processing pathways (Bahn et al., 2015). Finally, variation of ADAR expression in vivo will possibly be associated with modification of the hundreds of genes located on 1q and/or controlled by interferon. Determining whether increasing A-to-I editing limits or enhances cancer progression will need to take into account all of these potential variables. More research is needed to identify the critical editing sites, establish their potential as markers of cancer evolution, and investigate them as a new class of therapeutic targets.

EXPERIMENTAL PROCEDURES

The study has been approved by the Institut Jules Bordet Ethics Committee (approval number: CE1967). The methods are fully detailed in the Supplemental Experimental Procedures. In brief, the exome and transcriptome of 58 well-characterized BC samples representing the four main known subtypes based on immunohistochemistry, namely, TN, HER2$^+$, luminal A, and luminal B, and ten matched normal samples were profiled using exome sequencing and RNA-seq in paired-end mode on the Illumina HiSeq 2000 platform. Gene expression and SNPs profiles were obtained with Affymetrix HG-U133 Plus 2.0 Array chips and Affymetrix Genome-Wide Human SNP Arrays 6.0 for 57 and 49 tumor samples, respectively. RNA reads obtained from RNA-seq were aligned simultaneously on the human genome and all known exonic junctions. Variant calls were submitted to a series of filters limiting artifact associated with RNA-seq. The identified RNA-DNA differences (RDDs) were validated in an independent cohort of 15 BC samples; moreover, few events as well as their editing frequencies were validated using an independent technology (Roche FLX sequencer). The effect of interferon (IFN) on ADAR expression and editing was evaluated on six BC cell lines and one immortalized, non-transformed mammary epithelial cell line, MCF-10A. Cell lines were treated for 1, 2, or 5 days with IFN α, β, or γ. The effect of treatment on ADAR p110 and p150 protein and gene expression levels were evaluated quantifying the immunoblot signals and qRT-PCR data, respectively, while the effect of IFN treatment on editing distribution and frequency was investigated using amplicon sequencing (Roche FLX sequencer). In each sample, the mean editing frequency was correlated with clinico-pathological parameters and the expression of ADAR. The intracellular localization of ADAR was defined using immunohistochemistry. The association between editing and *ADAR* amplification and/or a surrogate of interferon response (STAT1 expression) was evaluated in breast and 19 additional cancer types obtained from TCGA. Finally, the effect of ADAR knockdown on cell proliferation, migration, and apoptosis was evaluated in three representative BC cell lines transduced with shRNA lentiviral particles.

ACCESSION NUMBERS

Sequencing and SNPs array data obtained from the enrolled patients are archived at European Genome-phenome Archive, https://www.ebi.ac.uk/ega, under accession number EGAS00001000495; amplicon sequencing data obtained from cell lines are archived at European Nucleotide Archive, http://www.ebi.ac.uk/ena, under study accession number ERP004253; gene expression array data are archived at the NCBI GEO, http://www.ncbi.nlm.

nih.gov/geo, under accession number GSE43358. Clinical information, results of sequence and arrays preprocessing, and biological assays are available in Tables S1, S2, S3, S4, S5, S6, and S7.

SUPPLEMENTAL INFORMATION

Supplemental Information includes Supplemental Experimental Procedures, seven figures, and seven tables and can be found with this article online at http://dx.doi.org/10.1016/j.celrep.2015.09.032.

AUTHOR CONTRIBUTIONS

C.S., V.D., and M.A. made substantial contributions to study conception and design. D.F., F.R., and N.K. acquired the data (acquired and managed patients/samples, performed cell lines experiments, etc.). A.L., F.L., and P.J.C. generated the sequencing data. R.S. and D.L. performed the pathology evaluation. K.P. generated the breast cancer organoids. D.G., V.D., D.B., T.K., A.S., C.S., D.F., and F.R. analyzed and interpreted the data (e.g., statistical analysis, biostatistics, computational analysis). V.D., D.F., C.S., C.D., D.G., K.W.-G., F.R., D.B., M.P., and R.S. made substantial contributions to writing, review, and/or revision of the manuscript. V.D. and C.S. supervised the study.

ACKNOWLEDGMENTS

This work was supported by a grant of the Belgian National Cancer Plan PNC29. D.G. and T.K. have been supported by a WELBIO grant. M.A., D.B., V.D., and C.S. were supported by the FNRS. A.S. is funded by the H.L. Holmes Award from the National Research Council Canada and an EMBO fellowship. C.D. has been supported by the Brussels Region. The authors thank Raphael Leplae and the ULB Computing Center for their support, Roland De Wind for pathology support, Cédric Blanpain, Sabine Costagliola, Jacques E. Dumont, Pierre Vanderhaeghen, and Gilbert Vassart for helpful discussions.

Received: March 13, 2015
Revised: July 13, 2015
Accepted: September 11, 2015
Published: October 1, 2015

REFERENCES

Alexandrov, L.B., Nik-Zainal, S., Wedge, D.C., Aparicio, S.A.J.R., Behjati, S., Biankin, A.V., Bignell, G.R., Bolli, N., Borg, A., Børresen-Dale, A.-L., et al.; Australian Pancreatic Cancer Genome Initiative; ICGC Breast Cancer Consortium; ICGC MMML-Seq Consortium; ICGC PedBrain (2013). Signatures of mutational processes in human cancer. Nature *500*, 415–421.

Athanasiadis, A., Rich, A., and Maas, S. (2004). Widespread A-to-I RNA editing of Alu-containing mRNAs in the human transcriptome. PLoS Biol. *2*, e391.

Avesson, L., and Barry, G. (2014). The emerging role of RNA and DNA editing in cancer. Biochim. Biophys. Acta *1845*, 308–316.

Bahn, J.H., Lee, J.-H., Li, G., Greer, C., Peng, G., and Xiao, X. (2012). Accurate identification of A-to-I RNA editing in human by transcriptome sequencing. Genome Res. *22*, 142–150.

Bahn, J.H., Ahn, J., Lin, X., Zhang, Q., Lee, J.-H., Civelek, M., and Xiao, X. (2015). Genomic analysis of ADAR1 binding and its involvement in multiple RNA processing pathways. Nat. Commun. *6*, 6355.

Bass, B.L. (2002). RNA editing by adenosine deaminases that act on RNA. Annu. Rev. Biochem. *71*, 817–846.

Bazak, L., Levanon, E.Y., and Eisenberg, E. (2014a). Genome-wide analysis of Alu editability. Nucleic Acids Res. *42*, 6876–6884.

Bazak, L., Haviv, A., Barak, M., Jacob-Hirsch, J., Deng, P., Zhang, R., Isaacs, F.J., Rechavi, G., Li, J.B., Eisenberg, E., and Levanon, E.Y. (2014b). A-to-I RNA editing occurs at over a hundred million genomic sites, located in a majority of human genes. Genome Res. *24*, 365–376.

Blow, M.J., Grocock, R.J., van Dongen, S., Enright, A.J., Dicks, E., Futreal, P.A., Wooster, R., and Stratton, M.R. (2006). RNA editing of human microRNAs. Genome Biol. *7*, R27.

Burns, M.B., Temiz, N.A., and Harris, R.S. (2013). Evidence for APOBEC3B mutagenesis in multiple human cancers. Nat. Genet. *45*, 977–983.

Cenci, C., Barzotti, R., Galeano, F., Corbelli, S., Rota, R., Massimi, L., Di Rocco, C., O'Connell, M.A., and Gallo, A. (2008). Down-regulation of RNA editing in pediatric astrocytomas: ADAR2 editing activity inhibits cell migration and proliferation. J. Biol. Chem. *283*, 7251–7260.

Chen, C.X., Cho, D.S., Wang, Q., Lai, F., Carter, K.C., and Nishikura, K. (2000). A third member of the RNA-specific adenosine deaminase gene family, ADAR3, contains both single- and double-stranded RNA binding domains. RNA *6*, 755–767.

Chen, L., Li, Y., Lin, C.H., Chan, T.H.M., Chow, R.K.K., Song, Y., Liu, M., Yuan, Y.-F., Fu, L., Kong, K.L., et al. (2013). Recoding RNA editing of AZIN1 predisposes to hepatocellular carcinoma. Nat. Med. *19*, 209–216.

Curtis, C., Shah, S.P., Chin, S.-F., Turashvili, G., Rueda, O.M., Dunning, M.J., Speed, D., Lynch, A.G., Samarajiwa, S., Yuan, Y., et al.; METABRIC Group (2012). The genomic and transcriptomic architecture of 2,000 breast tumours reveals novel subgroups. Nature *486*, 346–352.

Desterro, J.M.P., Keegan, L.P., Lafarga, M., Berciano, M.T., O'Connell, M., and Carmo-Fonseca, M. (2003). Dynamic association of RNA-editing enzymes with the nucleolus. J. Cell Sci. *116*, 1805–1818.

Efron, B., and Tibshirani, R. (2007). On testing the significance of sets of genes. Ann. Appl. Stat. *1*, 107–129.

Han, S.-W., Kim, H.-P., Shin, J.-Y., Jeong, E.-G., Lee, W.-C., Kim, K.Y., Park, S.Y., Lee, D.-W., Won, J.-K., Jeong, S.-Y., et al. (2014). RNA editing in RHOQ promotes invasion potential in colorectal cancer. J. Exp. Med. *211*, 613–621.

Heale, B.S.E., Keegan, L.P., McGurk, L., Michlewski, G., Brindle, J., Stanton, C.M., Caceres, J.F., and O'Connell, M.A. (2009). Editing independent effects of ADARs on the miRNA/siRNA pathways. EMBO J. *28*, 3145–3156.

Higuchi, M., Maas, S., Single, F.N., Hartner, J., Rozov, A., Burnashev, N., Feldmeyer, D., Sprengel, R., and Seeburg, P.H. (2000). Point mutation in an AMPA receptor gene rescues lethality in mice deficient in the RNA-editing enzyme ADAR2. Nature *406*, 78–81.

Hundley, H.A., and Bass, B.L. (2010). ADAR editing in double-stranded UTRs and other noncoding RNA sequences. Trends Biochem. Sci. *35*, 377–383.

Jiang, Q., Crews, L.A., Barrett, C.L., Chun, H.-J., Court, A.C., Isquith, J.M., Zipeto, M.A., Goff, D.J., Minden, M., Sadarangani, A., et al. (2013). ADAR1 promotes malignant progenitor reprogramming in chronic myeloid leukemia. Proc. Natl. Acad. Sci. USA *110*, 1041–1046.

Ju, Y.S., Kim, J.-I., Kim, S., Hong, D., Park, H., Shin, J.-Y., Lee, S., Lee, W.-C., Kim, S., Yu, S.-B., et al. (2011). Extensive genomic and transcriptional diversity identified through massively parallel DNA and RNA sequencing of eighteen Korean individuals. Nat. Genet. *43*, 745–752.

Kapusta, A., Kronenberg, Z., Lynch, V.J., Zhuo, X., Ramsay, L., Bourque, G., Yandell, M., and Feschotte, C. (2013). Transposable elements are major contributors to the origin, diversification, and regulation of vertebrate long noncoding RNAs. PLoS Genet. *9*, e1003470.

Kawahara, Y., Zinshteyn, B., Sethupathy, P., Iizasa, H., Hatzigeorgiou, A.G., and Nishikura, K. (2007). Redirection of silencing targets by adenosine-to-inosine editing of miRNAs. Science *315*, 1137–1140.

Kim, D.D.Y., Kim, T.T.Y., Walsh, T., Kobayashi, Y., Matise, T.C., Buyske, S., and Gabriel, A. (2004). Widespread RNA editing of embedded alu elements in the human transcriptome. Genome Res. *14*, 1719–1725.

Kiran, A., and Baranov, P.V. (2010). DARNED: a DAtabase of RNa EDiting in humans. Bioinformatics *26*, 1772–1776.

Levanon, E.Y., Eisenberg, E., Yelin, R., Nemzer, S., Hallegger, M., Shemesh, R., Fligelman, Z.Y., Shoshan, A., Pollock, S.R., Sztybel, D., et al. (2004). Systematic identification of abundant A-to-I editing sites in the human transcriptome. Nat. Biotechnol. *22*, 1001–1005.

Li, J.B., Levanon, E.Y., Yoon, J.-K., Aach, J., Xie, B., Leproust, E., Zhang, K., Gao, Y., and Church, G.M. (2009). Genome-wide identification of human

RNA editing sites by parallel DNA capturing and sequencing. Science *324*, 1210–1213.

Li, M., Wang, I.X., Li, Y., Bruzel, A., Richards, A.L., Toung, J.M., and Cheung, V.G. (2011). Widespread RNA and DNA sequence differences in the human transcriptome. Science *333*, 53–58.

Maas, S., Patt, S., Schrey, M., and Rich, A. (2001). Underediting of glutamate receptor GluR-B mRNA in malignant gliomas. Proc. Natl. Acad. Sci. USA *98*, 14687–14692.

Mo, F., Wyatt, A.W., Sun, Y., Brahmbhatt, S., McConeghy, B.J., Wu, C., Wang, Y., Gleave, M.E., Volik, S.V., and Collins, C.C. (2014). Systematic identification and characterization of RNA editing in prostate tumors. PLoS ONE *9*, e101431.

Nik-Zainal, S., Alexandrov, L.B., Wedge, D.C., Van Loo, P., Greenman, C.D., Raine, K., Jones, D., Hinton, J., Marshall, J., Stebbings, L.A., et al.; Breast Cancer Working Group of the International Cancer Genome Consortium (2012). Mutational processes molding the genomes of 21 breast cancers. Cell *149*, 979–993.

Nishikura, K. (2010). Functions and regulation of RNA editing by ADAR deaminases. Annu. Rev. Biochem. *79*, 321–349.

Ota, H., Sakurai, M., Gupta, R., Valente, L., Wulff, B.-E., Ariyoshi, K., Iizasa, H., Davuluri, R.V., and Nishikura, K. (2013). ADAR1 forms a complex with Dicer to promote microRNA processing and RNA-induced gene silencing. Cell *153*, 575–589.

Park, E., Williams, B., Wold, B.J., and Mortazavi, A. (2012). RNA editing in the human ENCODE RNA-seq data. Genome Res. *22*, 1626–1633.

Patterson, J.B., and Samuel, C.E. (1995). Expression and regulation by interferon of a double-stranded-RNA-specific adenosine deaminase from human cells: evidence for two forms of the deaminase. Mol. Cell. Biol. *15*, 5376–5388.

Paz, N., Levanon, E.Y., Amariglio, N., Heimberger, A.B., Ram, Z., Constantini, S., Barbash, Z.S., Adamsky, K., Safran, M., Hirschberg, A., et al. (2007). Altered adenosine-to-inosine RNA editing in human cancer. Genome Res. *17*, 1586–1595.

Paz-Yaacov, N., Bazak, L., Buchumenski, I., Porath, H.T., Danan-Gotthold, M., Knisbacher, B.A., Eisenberg, E., and Levanon, E.Y. (2015). Elevated RNA editing activity is a major contributor to transcriptomic diversity in tumors. Cell Rep. *13*, this issue, 267–276.

Peng, Z., Cheng, Y., Tan, B.C.-M., Kang, L., Tian, Z., Zhu, Y., Zhang, W., Liang, Y., Hu, X., Tan, X., et al. (2012). Comprehensive analysis of RNA-Seq data reveals extensive RNA editing in a human transcriptome. Nat. Biotechnol. *30*, 253–260.

Qin, Y.-R., Qiao, J.-J., Chan, T.H.M., Zhu, Y.-H., Li, F.-F., Liu, H., Fei, J., Li, Y., Guan, X.-Y., and Chen, L. (2014). Adenosine-to-inosine RNA editing mediated by ADARs in esophageal squamous cell carcinoma. Cancer Res. *74*, 840–851.

Ramaswami, G., Lin, W., Piskol, R., Tan, M.H., Davis, C., and Li, J.B. (2012). Accurate identification of human Alu and non-Alu RNA editing sites. Nat. Methods *9*, 579–581.

Ramaswami, G., Zhang, R., Piskol, R., Keegan, L.P., Deng, P., O'Connell, M.A., and Li, J.B. (2013). Identifying RNA editing sites using RNA sequencing data alone. Nat. Methods *10*, 128–132.

Roberts, S.A., Lawrence, M.S., Klimczak, L.J., Grimm, S.A., Fargo, D., Stojanov, P., Kiezun, A., Kryukov, G.V., Carter, S.L., Saksena, G., et al. (2013). An APOBEC cytidine deaminase mutagenesis pattern is widespread in human cancers. Nat. Genet. *45*, 970–976.

Rueter, S.M., Dawson, T.R., and Emeson, R.B. (1999). Regulation of alternative splicing by RNA editing. Nature *399*, 75–80.

Salameh, A., Lee, A.K., Cardó-Vila, M., Nunes, D.N., Efstathiou, E., Staquicini, F.I., Dobroff, A.S., Marchiò, S., Navone, N.M., Hosoya, H., et al. (2015). PRUNE2 is a human prostate cancer suppressor regulated by the intronic long noncoding RNA PCA3. Proc. Natl. Acad. Sci. USA *112*, 8403–8408.

Sansam, C.L., Wells, K.S., and Emeson, R.B. (2003). Modulation of RNA editing by functional nucleolar sequestration of ADAR2. Proc. Natl. Acad. Sci. USA *100*, 14018–14023.

Savva, Y.A., Rieder, L.E., and Reenan, R.A. (2012). The ADAR protein family. Genome Biol. *13*, 252.

Schoggins, J.W., Wilson, S.J., Panis, M., Murphy, M.Y., Jones, C.T., Bieniasz, P., and Rice, C.M. (2011). A diverse range of gene products are effectors of the type I interferon antiviral response. Nature *472*, 481–485.

Shah, S.P., Morin, R.D., Khattra, J., Prentice, L., Pugh, T., Burleigh, A., Delaney, A., Gelmon, K., Guliany, R., Senz, J., et al. (2009). Mutational evolution in a lobular breast tumour profiled at single nucleotide resolution. Nature *461*, 809–813.

Shoshan, E., Mobley, A.K., Braeuer, R.R., Kamiya, T., Huang, L., Vasquez, M.E., Salameh, A., Lee, H.J., Kim, S.J., Ivan, C., et al. (2015). Reduced adenosine-to-inosine miR-455-5p editing promotes melanoma growth and metastasis. Nat. Cell Biol. *17*, 311–321.

Swanton, C., McGranahan, N., Starrett, G.J., and Harris, R.S. (2015). APOBEC Enzymes: Mutagenic Fuel for Cancer Evolution and Heterogeneity. Cancer Discov. *5*, 704–712.

Tomaselli, S., Bonamassa, B., Alisi, A., Nobili, V., Locatelli, F., and Gallo, A. (2013). ADAR enzyme and miRNA story: a nucleotide that can make the difference. Int. J. Mol. Sci. *14*, 22796–22816.

Wang, Q., Khillan, J., Gadue, P., and Nishikura, K. (2000). Requirement of the RNA editing deaminase ADAR1 gene for embryonic erythropoiesis. Science *290*, 1765–1768.

Wang, I.X., So, E., Devlin, J.L., Zhao, Y., Wu, M., and Cheung, V.G. (2013). ADAR regulates RNA editing, transcript stability, and gene expression. Cell Rep. *5*, 849–860.

Witkin, K.L., Hanlon, S.E., Strasburger, J.A., Coffin, J.M., Jaffrey, S.R., Howcroft, T.K., Dedon, P.C., Steitz, J.A., Daschner, P.J., and Read-Connole, E. (2015). RNA editing, epitranscriptomics, and processing in cancer progression. Cancer Biol. Ther. *16*, 21–27.

Yang, W., Chendrimada, T.P., Wang, Q., Higuchi, M., Seeburg, P.H., Shiekhattar, R., and Nishikura, K. (2006). Modulation of microRNA processing and expression through RNA editing by ADAR deaminases. Nat. Struct. Mol. Biol. *13*, 13–21.

Zhang, L., Zhou, Y., Cheng, C., Cui, H., Cheng, L., Kong, P., Wang, J., Li, Y., Chen, W., Song, B., et al. (2015). Genomic analyses reveal mutational signatures and frequently altered genes in esophageal squamous cell carcinoma. Am. J. Hum. Genet. *96*, 597–611.

Han, L., Diao, L., Yu, S., Xu, X., Li, J., Zhang, R., Yang, Y., Werner, H.M.J., Eterovic, A.K., Yuan, Y., et al. (2015). The genomic landscape and clinical relevance of A-to-I RNA editing in human cancers. Cancer Cell *28*, this issue, 515–528.

Cell Reports

Supplemental Information

Principles Governing A-to-I RNA Editing in the Breast Cancer Transcriptome

Debora Fumagalli, David Gacquer, Françoise Rothé, Anne Lefort, Frederick Libert, David Brown, Naima Kheddoumi, Adam Shlien, Tomasz Konopka, Roberto Salgado, Denis Larsimont, Kornelia Polyak, Karen Willard-Gallo, Christine Desmedt, Martine Piccart, Marc Abramowicz, Peter J. Campbell, Christos Sotiriou, and Vincent Detours

Supplemental Information

Supplemental Figures 2

Supplemental Tables...9

Supplemental Experimental Procedures 10
Patients and sample characterization and preparation 10
Detection of RNA-DNA differences...11
Protein expression, mRNA expression and DNA copy number profiling 15
Cell lines experiments...16
Statistical Analysis 19

Supplemental References 22

Supplemental Figures

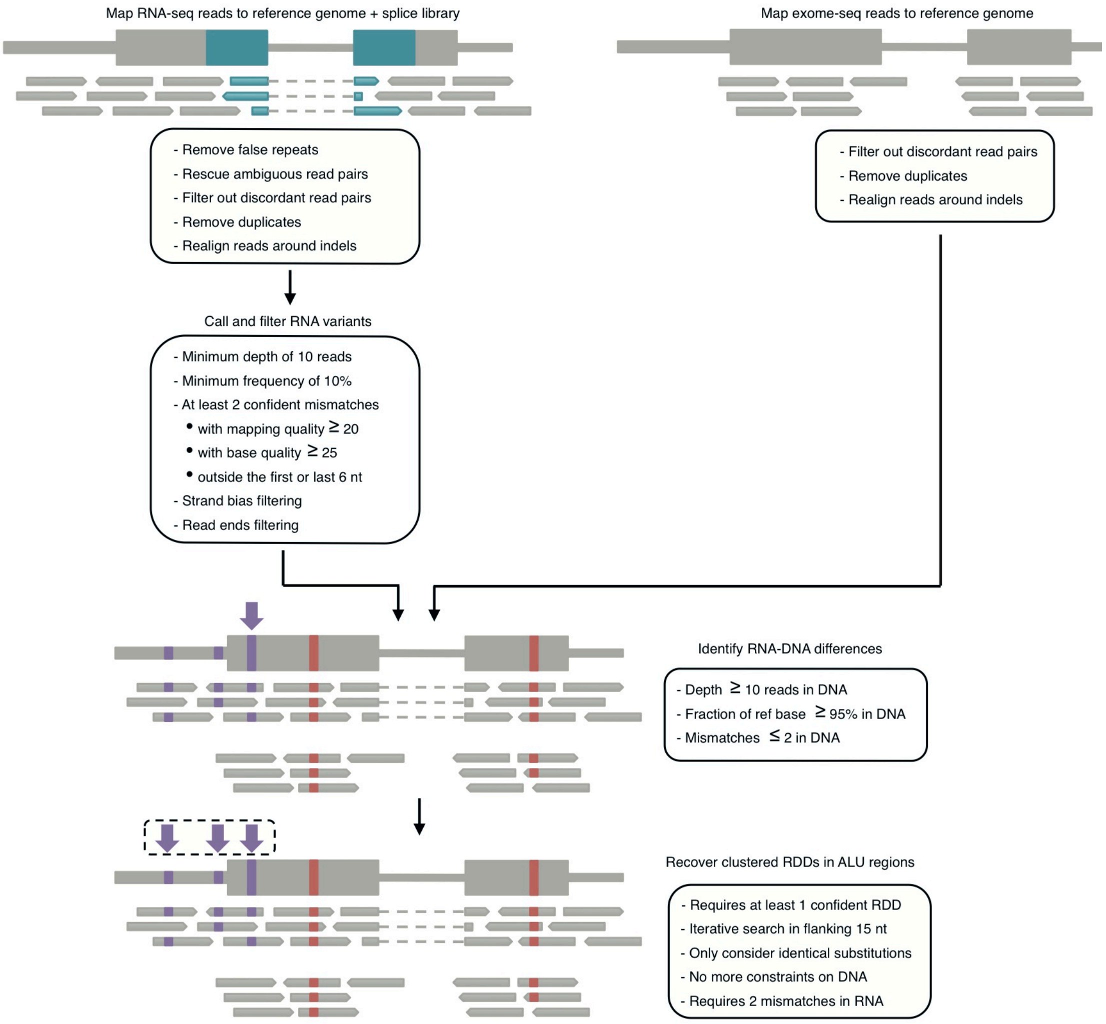

Figure S1. Overview of the pipeline for RNA/DNA differences (RDDs) detection (Related to Figures 1-5). Details are presented in the Supplemental Experimental Procedures.

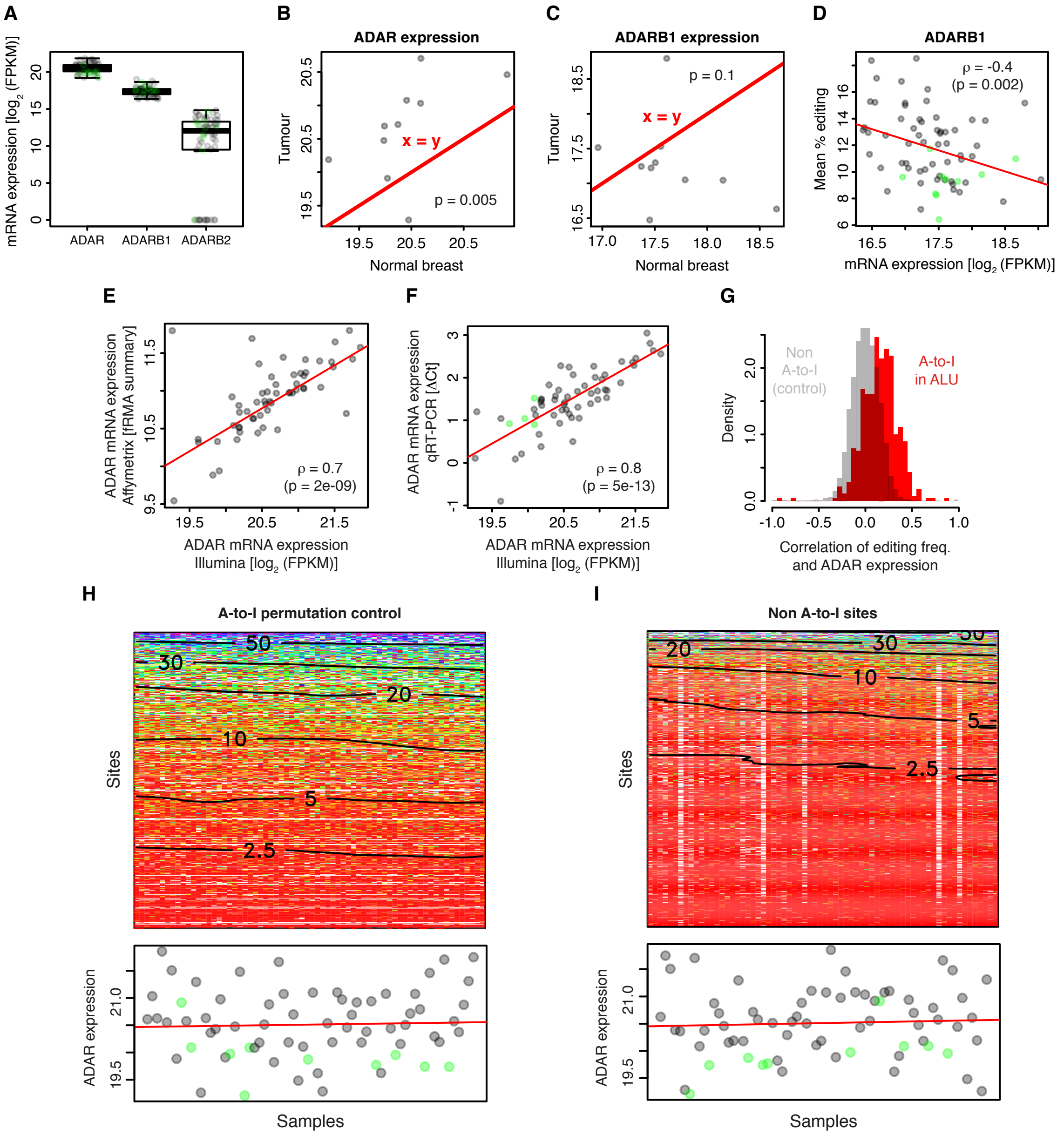

Figure S2. Expression and correlation with A-to-I RNA editing for the *ADAR* isoforms (A-D, Related to Figures 2 and 3) and (E-I) additional controls associated with Figure 3. (A), Expression of *ADAR*, *ADARB1* and *ADARB2* across our cohort. **(B)**, Each point represents a sample with the expression of *ADAR* in the normal breast tissue depicted on the x-axis and the expression in the matched tumor breast tissue on the y-axis. All but one point are above the x=y identity line, demonstrating that *ADAR* expression is higher in tumors than in normal tissues. The p-value was calculated from a Wilcoxon paired signed test. **(C)**, Same as **(B)** for *ADARB1*. **(D)**, each point represents a sample with *ADARB1* expression on the x-axis and the mean editing frequency on the y-axis. *ADAR* expression quantification from whole transcriptome sequencing is highly consistent with, (**E**), Affymetrix microarray and, (**F**), qRT-PCR quantifications. (**G**), Distribution of Spearman's correlations across the samples of the RNA-seq expression of *ADAR* and the editing frequencies of individual sites: 560 Alu A-to-I sites (red), and as negative control 11,312 putative non A-to-I RDDs (grey). (**H** and **I**), Heatmaps of editing frequencies after random permutation editing of frequencies across samples, depicted in panel (**H**), and of non-A-to-I putative RDDs, depicted in panel (**I**). The gradient in these negative control heatmaps is from top-to-bottom, without left-to-right component. Green dots represent tumor-matched normal samples.

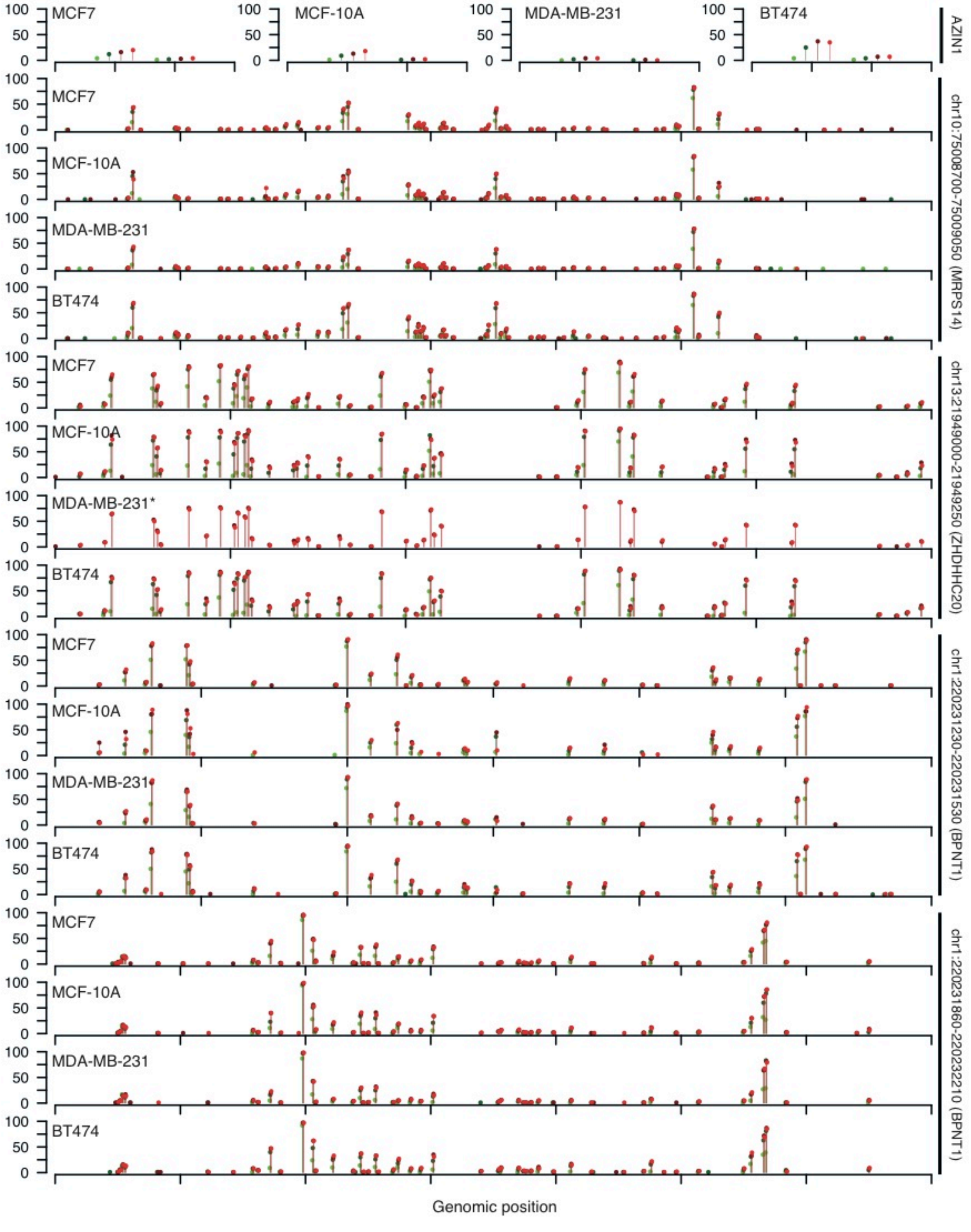

Figure S3. The same sites are edited in four breast cell lines (three tumor and one normal tissue derived cell lines) and increasing *ADAR* expression increases the editing frequency at all these sites (Related to Figure 4A). Editing in *AZIN1* and 4 *Alu* regions is shown in 4 breast cell lines and 4 ADAR protein expression levels (same color scale as in Figure 4A). The x-axis scales are different for each region (see right-side labels). (*) Library preparation failed for the two lower ADAR expression samples for MDA-MB-231, chr13:21949000-21949250.

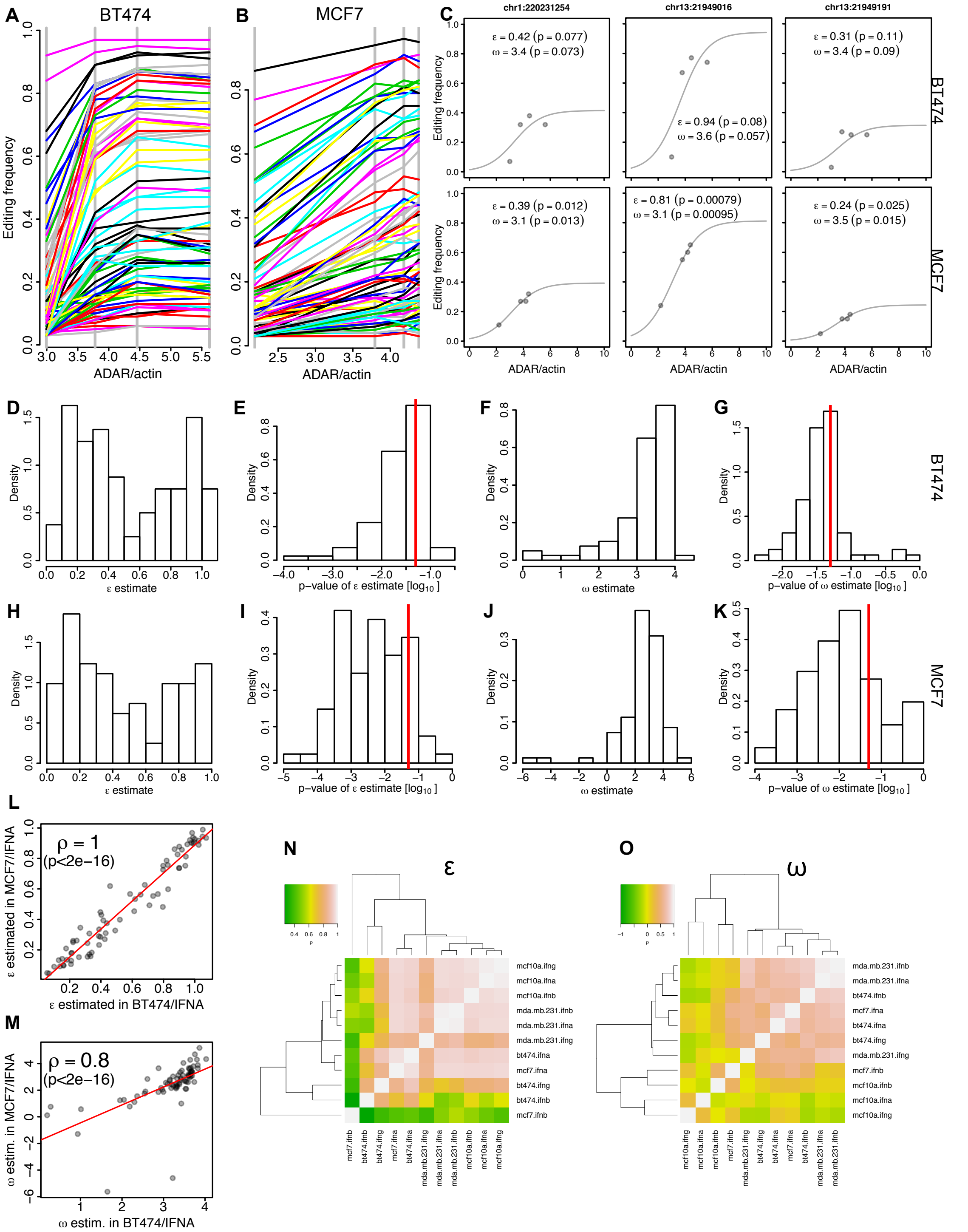

Figure S4. Modeling editing frequency with the logistic function (Related to Figures 4E-H). (**A** and **B**) Dose-response curves for cell lines BT474 and MCF7 (**A** also shown in main text). ADAR was induced via interferon treatment. Note that saturation is reached for the third and fourth points for BT474, but not MCF7. (**C**), Fits of the logistic model to dose-response data for three editing sites are shown. (**D-K**), Overview of all the logistic fits for dose-response curves shown in (**A**) and (**B**), with distribution of ε_i (**D**, **H**), ω_i (**F**, **J**) and associated p-values (**E**, **I**, **G** and **K**; Red lines denote the p=0.05 limit). ε_i but not ω_i estimates are distributed around a central value, suggesting that ε_i, but not ω_i, is site-specific. (**L** and **M**), comparison of the logistic parameters estimated from the BT474 and MCF7 experiments. (**N** and **O**), Correlations of ε_i, and ω_i, between all pairs of interferon treatment experiments performed in this study for which enough data was available. Lower correlations typically resulted from aberrant fits caused by data scarcity, the fits rest on 4 data points, and suboptimal doses.

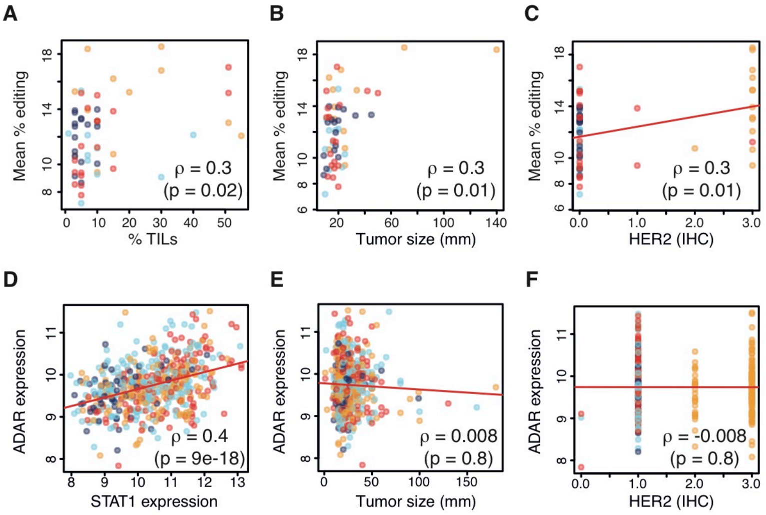

G

		Multivariate p-values			
	Univariate	TILs and HER2	TILs and T size	HER2 and T size	All
% TILs	0.01	0.07	0.02		0.06
Tumor size	0.0001		0.0002	0.0009	0.0009
HER2 (IHC)	0.004	0.02		0.04	0.13

Figure S5. Correlations of A-to-I editing and breast cancer clinico-pathological variables in our cohort (Related to Figure 2). The mean editing frequency is significantly correlated with (**A**), the proportion of tumor-infiltrating lymphocytes (TILs), (**B**), tumor size and (**C**), HER2 defined by immuno-histochemistry. Point colors depict subtypes: navy blue, luminal A; sky blue, luminal B; orange, HER2; red, triple negative. These associations were tested in 787 patients of the Metabric cohort (Curtis et al., 2012) for whom HER2 IHC was available. *ADAR* expression (a surrogate for the editing frequency, Figure 3A) and *STAT1* expression (a surrogate for TILs) were used in this analysis. An association was found with *STAT1* (**D**), but not tumor size (**E**) and HER2 IHC (**F**). (**G**), A multivariate analysis demonstrates that the associations of editing with HER2 and TILs are statistically related. Multivariate analysis decreases the significance of all variables when all three are combined together. We conducted bi-variate analyses to dispel the ambiguity of variables' dependencies. HER2 status and TILs were less significant when analyzed together than with tumor size. Thus, the dependency is mostly between TILs and HER2 status. Importantly, this figure depicts only significant associations. No significant correlations could be found between mean editing frequency and adipose content, stromal content, grade, nodal status, and ER, PGR and Ki67 immuno-histochemistry staining.

A

gene set	GSA score	p-value	FDR
KEGG_RIG_I_LIKE_RECEPTOR_SIGNALING_PATHWAY	0.3981	0	0
KEGG_CYTOSOLIC_DNA_SENSING_PATHWAY	0.6729	0	0
ST_TUMOR_NECROSIS_FACTOR_PATHWAY	0.6677	0	0
SIG_PIP3_SIGNALING_IN_B_LYMPHOCYTES	0.6815	0	0
PID_TXA2PATHWAY	0.6138	0	0
REACTOME_IMMUNOREGULATORY_INTERACTIONS_BETWEEN_A_LYMPHOID_AND_A_NON_LYMPHOID_CELL	1.2665	0	0
REACTOME_INTERFERON_ALPHA_BETA_SIGNALING	1.8057	0	0
REACTOME_INTERFERON_SIGNALING	0.8589	0	0
REACTOME_RIG_I_MDA5_MEDIATED_INDUCTION_OF_IFN_ALPHA_BETA_PATHWAYS	0.5511	0	0
REACTOME_CYTOKINE_SIGNALING_IN_IMMUNE_SYSTEM	0.5604	0	0
REACTOME_INFLAMMASOMES	0.869	0	0

B

gene set	GSA score	p-value	FDR
V$NFKAPPAB_01	0.2987	0	0
V$IRF1_01	0.4256	0	0
V$IRF2_01	0.5835	0	0
V$ISRE_01	0.5547	0	0
V$IRF7_01	0.5173	0	0
V$ICSBP_Q6	0.4919	0	0
STTTCRNTTT_V$IRF_Q6	0.8069	0	0

C

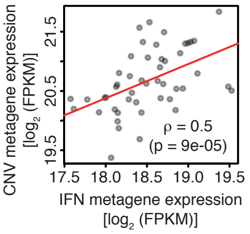

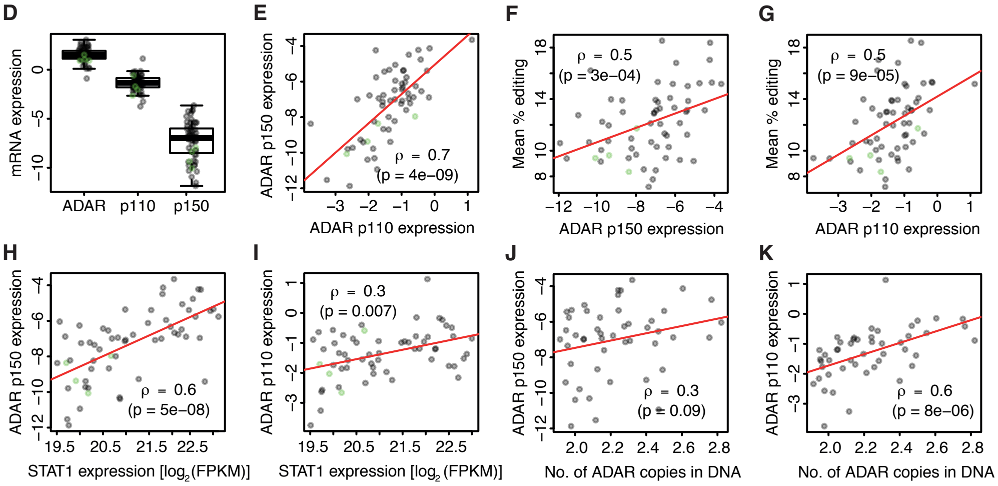

Figure S6. Gene set and metagenes analysis of the correlation of A-to-I editing with gene expression (A-C), and expressions of *ADAR* isoforms p110 and p150 support a control of *ADAR* expression by 1q amplification and interferon (D-K) (Related to Figure 5). Affymetrix expression data were adjusted for *ADAR* DNA copy number and then screened for gene sets associated with the mean editing frequency. (**A**), Screen of gene sets defining canonical pathways. (**B**), Screen of genes set defined by genes sharing binding motifs for the same transcription factor in their promoter. Null p-values and false discovery rate (FDR) means that no random gene sets in 500 had a higher GSA score (see Supplemental Experimental Procedures). (**C**), Correlation between DNA copy number-adjusted *ADAR* expression and the median expression of 389 interferon-induced genes compiled from 10 studies. The relative expression of *ADAR,* its constitutively active form, p110, and the interferon-inducible form, p150, were measured by qRT-PCR for 58 samples (see Table S3), depicted as individual data points in the panels. (**D**), The truncated form of *ADAR*, p110, predominates over the full-length transcript, p150. (**E**), The expressions of the two isoforms are highly correlated and, (**F** and **G**), are correlated with the mean editing frequency. *STAT1* is correlated with the expression of interferon-inducible p150 (**H**), but less with the expression of p110 (**I**). Conversely, the correlation with *ADAR* copy number is lower for p150 (**J**) than p110 (**K**) — in agreement with the notion that the association of p150 with *ADAR* copy number is confounded by its strong dependence on interferon control. Green dots represent tumor-matched normal samples.

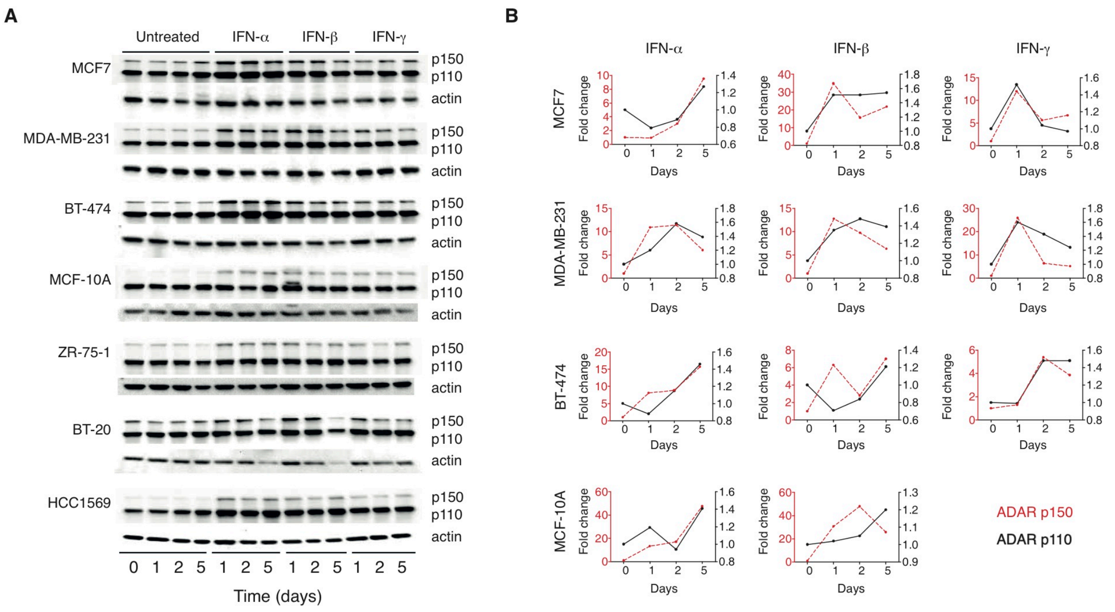

Figure S7. Interferon treatments increase *ADAR* mRNA and protein expressions in breast cancer cell lines (Related to Figure 5). (**A**), Western blots underlying Figure 5E and Table S6. (**B**), qRT-PCR for *ADAR* p110 and p150 (see also Table S7). The expressions of p110 and p150 are presented as fold changes relative to the expression of the untreated cells. Note the different *y*-axis scales used for the two isoforms. The scales for p150 are much larger.

Supplemental Tables

Supplemental tables are provided as online excel files.

- Table S1: **Patients data (Related to Figures 1-5**). Clinic-pathologic data of the patients involved in the study.

- Table S2: **Putative RNA-DNA differences (RDDs) in *in vivo* samples (Related to Figures 1-5)**. Characterization of the 16,027 RDDs identified in the patients under study. This data is necessary to reproduce most calculation in the paper.

- Table S3: **Sample data (Related to Figures 1-5)**. For each patient involved in the study, this table reports key information necessary to reproduce most analyses and figures in the paper.

- Table S4: **Comparison of (A) A-to-I RNA editing studies and (B) detection pipelines (Related to Figure 1).** These tables report the comparison between the current study and the most relevant ones published in the field in the last years with what regards (A) their features and (B) their detection pipelines.

- Table S5: **AZIN1 editing measured by amplicon sequencing in 30 patient-matched tumor/normal pairs (Related to Figure 2)**. Values representing the editing of AZIN1 measured by amplicon sequencing (Roche FLX) in tumor and normal matched pairs of 30 study patients.

- Table S6: **ADAR protein expression quantification (A) and editing frequency (B) in *in vitro* IFN experiments (Related to Figures 4 and 5).** For cell lines treated with IFN α, β and γ for 24h, 48h and 120h, these tables report: (A) the ADAR protein expression determined by Western blot, and (B) the editing frequency of the sites investigated with amplicon sequencing.

- Table S7: **ADAR RT-PCR mRNA expression in *in vitro* IFN experiments Related to Figures 4 and 5).** For cell lines treated with IFN α, β and γ for 24h, 48h and 120h, this table reports the expression of ADAR determined with RT-PCR.

Supplemental Experimental Procedures

Patients and samples characterization and preparation

Samples selection. A total of 58 breast cancer (BC) patients for whom both fresh-frozen tumor and matched normal breast tissue, as well as formalin-fixed paraffin embedded (FFPE) matched tumor breast tissue were available at Jules Bordet Institute Tumor Bank (Jules Bordet Institute, Brussels, Belgium) were selected for this project. Patients were recruited between 2007 and 2011; the associated clinico-pathological data can be found in Table S1.

The use of the data is consistent with the informed consent signed by the patients or has been granted ethical approval by the local Ethics Committee and is in accordance with the applicable laws and regulations of Belgium. The study has been approved by the local Ethics Committee (approval number: CE1967).

Samples histopathology. On the basis of their immunohistochemistry (IHC) profile, patients were classified in one of the principle IHC BC subtypes: triple negative (TN: estrogen receptor (ER), progesterone receptor (PgR), and human epidermal growth factor receptor 2 (HER2) negative), HER2 positive (any ER and PgR, HER2 positive), luminal A (ER positive, HER2 negative, histological grade 1) and luminal B (ER positive, HER2 negative, histological grade 3).

The ER and PgR status was defined using the anti-estrogen receptor antibody [SP1] (ab166600, Abcam®, Cambridge, UK) and the anti-progesterone receptor antibody [1E2] (Roche, Basel, Switzerland), respectively. The staining was scored according to Allred (Harvey et al., 1999; Leake et al., 2000) using a combined score for proportion and intensity, and was considered as positive if the global score was >2. The HER2 status was defined using the antiHER2/neu antibody (4B5) (Roche). The scoring and subsequent FISH-analyses were done in accordance to the ASCO-CAP Guidelines on HER2-testing (Wolff et al., 2007). The histological grade was defined using the modified Bloom-Richardson grading system (Elston and Ellis, 1991; Genestie et al., 1998). The Ki67 staining was performed using the Monoclonal Mouse Anti-Human Ki-67 Antigen (Clone MIB-1) (Dako, Glostrup, Denmark).

For each sample, an hematoxylin and eosin (H&E) slide was made and was reviewed by a breast pathologist to confirm that the tumor specimen contained at least 30% of tumor cell nuclei and that the matched, adjacent normal specimen contained no tumor cells. Evaluation of the quantity and location (stromal or intratumoral) of tumor-infiltrating lymphocytes (TILs) was defined as described previously (Denkert et al., 2010).

DNA Extraction. DNA from both tumor and matched normal fresh-frozen tissues was extracted using the DNeasy Blood and Tissue kit® (Qiagen, Venlo, Netherlands) following the manufacturer's instructions. DNA concentration was measured using the NanoDrop 1000 instrument (Thermo Scientific, Waltham, Massachusetts). All the samples yielded enough material for downstream analyses.

RNA Extraction. RNA from both tumor and normal fresh-frozen tissues as well as from cell lines was extracted using TRIzol® (Life Technologies, Carlsbad, California) following the manufacturer's instructions. RNA concentration was defined using the NanoDrop 1000, and RNA integrity (RIN: RNA Integrity Number) was assessed using an Agilent 2100 Bioanalyzer (Agilent Technologies, Santa Clara, California).

All the samples yielded enough material for downstream analyses and had a RIN equal or superior to 6.5.

Purification of organoids from primary breast tissues. The protocol is described in details elsewhere (Allinen et al., 2004; Choudhury et al., 2013).

Detection of RNA-DNA differences

The analysis pipeline for the detection of RNA-DNA differences (RDDs) is summarized in Figure S1 and described in details in the following sections.

RNA Sequencing. Transcriptome sequencing was performed at DNAVision (Gosselie, Belgium). Transcriptome libraries from 58 tumor and 10 matched normal samples were constructed using the Illumina® TruSeq™ RNA Sample Preparation Kit for paired end reads sequencing on the HiSeq 2000 (Illumina, San Diego, California) following the manufacturer's instructions.

Briefly, starting from 1 µg of total RNA, the poly-A containing mRNA molecules were purified using poly-T oligo-attached magnetic beads. Following purification, the mRNA was fragmented into small pieces using divalent cations under elevated temperature. The cleaved RNA fragments were copied into first strand cDNA using reverse transcriptase and random primers. This was followed by second strand cDNA synthesis using DNA Polymerase I and RNase H and purification using the AMPure XP beads (Agencourt BioSciences Corporation, Beverly, Massachusetts). The cDNA fragments went through an end repair process, the addition of a single 'A' base and ligation of the adapters. The products were purified using the AMPure XP beads and enriched with PCR (15 cycles) to create the final cDNA library followed by purification using the AMPure XP beads. Libraries' quality control and quantification were performed using the Agilent Bioanalyser 2100 and qRT-PCR; libraries were pooled (4 libraries/pool). Clusters were generated in a cBot Cluster Generation System using the Paired-End Cluster Generation Kit v2-HS and sequenced on the Illumina HiSeq 2000 platform with a 2x50 base-pairs (BP) paired-end mode.

Exome Sequencing. Exome sequencing was performed at GATC (Konstanz, Germany). Genomic libraries from the tumor and matched normal samples were generated using the Illumina Paired End DNA sample preparation kit (Illumina) following the manufacturer's instructions. Enrichment was performed using the Agilent SureSelect Human All Exon V3 kit (Agilent) following the manufacturer's instructions.

Briefly, 2-3 µg of total genomic DNA was randomly fragmented to between 150 and 600bp by focused acoustic shearing (Covaris Inc, Wouburn, Massachusetts). A cleanup was performed using AMPure beads (Agencourt BioSciences Corporation) following the manufacturer's protocol and quality of the material was assessed using the Agilent Bioanalyser 2100. The size fractionated DNA was end repaired using T4 DNA polymerase, Klenow polymerase and T4 polynucleotide kinase and purified using AMPure beads. The resulting blunt ended fragments were A-tailed using a 3′-5′ exonuclease-deficient Klenow fragment, purified using AMPure beads and ligated to Illumina paired-end adaptor oligonucleotides in a 'TA' ligation at 20°C for 15 minutes. The product was purified using AMPure beads. After estimation of the concentration, the adaptor-ligated library was amplified and then purified using AMPure beads. Quality and quantity were assessed using an Agilent 2100 Bioanalyzer. The enriched regions were captured, purified, PCR amplified and purified using AMPure beads. After quantification and quality control of the captured library, samples were pooled (four samples/lane) for loading on an Illumina HiSeq

2000. Samples were sequenced in paired-end mode, with a read length of 2x100 bases.

Transcriptome read mapping. Because transcriptome read mapping is a key step to identify differences between RNA and DNA, we designed a dedicated framework to handle common errors associated to spliced reads. RNA reads were mapped simultaneously on the human reference genome (hg19) and a dedicated library of splice junction sequences using the Burrows-Wheeler Aligner (Li and Durbin, 2010) (BWA v0.5.9). We chose the BWA aligner due to its ability to handle gapped alignment and to report multiple matches for each read, which is required to identify reads mapping equally to the genome and the corresponding splice junction or to solve ambiguous read pairing. Paired reads were mapped independently with the command '`bwa aln –n 6`' to report up to 6 matches for reads that can be aligned to multiple places. Splice junctions were designed by concatenating respectively the last and first 50 nucleotides for each pair of consecutive exons. We used gene annotations from Refseq, UCSC, Ensembl and Gencode, downloaded from the UCSC Table Browser (Karolchik et al., 2004). Junctions common to two or more annotation sources were added only once to the library. Also, because BWA concatenates all chromosome sequences before indexing the reference, buffers of 20 N letters were added at each extremity of the splice junctions. This prevents BWA from producing irrelevant alignments extending outside the boundaries of reference sequences. For exons shorter than 50 nucleotides, this procedure would add adjacent intronic bases immediately upstream or downstream of this exon to meet the required splice sequence length. Such additions could further introduce inaccurate mapping and recurrent alignment errors around splice junctions. To solve this issue, we used an incremental approach to create splice site sequences, allowing as many exons as necessary to meet the required sequence length of 100 nucleotides. After alignment, coordinates of reads mapped on splice junctions were converted to the hg19 coordinate system.

Trimmed RNA reads could be shorter than 50 bases, thus some could be equally placed on a splice junction and its genomic counterpart. In this case, unique matches could be mistaken with repeats and incorrectly discarded. After alignment, all matches reported for a given read were processed to remove those that were identical once alignments on splice sites were reverted back to hg19 coordinates.

Paired-end sequencing often implies a pair rescue step in which ambiguous alignments can be fixed based on strand orientation and distance between mates. However, in the context of RNA sequencing, this procedure can sometimes introduce recurrent mismatches. When paired reads are processed independently, 'bwa aln' uniquely map them on the correct genomic location. However, when running 'bwa sampe' to perform read pairing, incorrect alignments can be preferred if they form a pair matching the expected insert size and strand orientation, even in presence of multiple mismatches. This mainly occurs for processed pseudogenes, because 'bwa sampe' does not compute the distance between mates with regard to the skipped introns spanned by transcriptome reads.

To solve this issue without losing the benefit of paired-end information, we implemented our own read-pairing step similar to 'bwa sampe'. For each read pair for which either one or both mates could not be uniquely mapped, we considered all possible correct pairings minimizing the cumulative edit distance over both mates. Read pairing was considered correct if strand orientation and inner distance between mates, after subtraction of intronic sequences between them, matched the Illumina

sequencing protocol. If multiple best pairings were found, both mates were flagged as repeats and discarded. Otherwise, the best pair was selected and both reads were considered unique. During this step, reads identified as repeats when mapped independently could be recovered as unique if they belonged to a single best pairing. However, unlike 'bwa sampe', we did not implement a Smith-Waterman local alignment to rescue read pairs where only one mate could not be mapped.

Once non-canonical read pairs were discarded, duplicates were removed with Picard's MarkDuplicates utility (v1.59) (http://broadinstitute.github.io/picard/) with default parameters and reads were realigned locally using the GATK's IndelRealigner program (v1.4-15) (McKenna et al., 2010). Local realignment was run with options '--knownAlleles known.indels.vcf --consensusDeterminationModel USE_READS' --maxConsensuses 50 --maxReadsForRealignment 400000 --maxReadsInMemory 300000', where known.indels.vcf was downloaded from the GATK resource bundle.

Exome read mapping. Paired-end reads from exome sequencing were mapped to the hg19 reference genome using BWA with default settings. As for transcriptome reads, only concordant unique read pairs were used. Duplicates were further removed using Picard and remaining reads were realigned locally with GATK. Both programs were used with the same parameters as for transcriptome alignments.

Identification of RNA-DNA differences. We identified single nucleotide substitutions based on pileup alignments. Pileup was computed using SAMtools (v0.1.18) (Li et al., 2009) with the command '`samtools mpileup -B -D -d 100000 -f hg19.fa in.bam | pileup-to-vcf.pl`', where pileup-to-vcf.pl was an in-house program designed to call a variant if the following conditions are met at a given position: 1) minimum depth of 10 reads, 2) minimum alternate allele frequency of 10 percent, 3) a minimum of 2 confident mismatches with base quality equal or greater to 25, 4) located in reads with a mapping quality of 20 or more and 5) not within the first of last 6 nucleotides of this read. Variants were then filtered based on strand bias and distance to read ends, to discard low confidence candidates relying on mismatches whose position relative to the query sequence harbors a suspicious pattern. Substitutions were further identified as RDDs if the corresponding position in DNA was homozygous for the reference. This implied 6) a minimum coverage of 10 reads in DNA, 7) a fraction of reference base equal to or greater than 95 percents and 8) allowing a maximum of 2 mismatches. However, because recent studies show that some dbSNP entries correspond to RNA edits (Eisenberg et al., 2005), we did not remove RDDs matching a known record from dbSNP (Sherry et al., 2001) v135.

Recovery of clustered RDDs. Due to the low coverage in certain regions of our transcriptome datasets, many rare edits were lost considering our minimum depth and frequency thresholds. Previous studies (Ju et al., 2011; Peng et al., 2012; Ramaswami et al., 2012) also report that a large fraction of RNA edits are located within untranslated region of genes, which are poorly covered by exome libraries. ADAR mediated editing is known to operate on double-stranded RNA duplexes often caused by the presence of inverted Alu elements. As a consequence, RNA edits are often clustered in genomic regions corresponding to both strands of the latter duplexes. Based on the hypothesis that multiple edits are likely to be close one from each other, we added an additional step to our pipeline to rescue rare edits clustered with high confidence candidates. We selected all edits called within Alu elements, based on UCSC RepeatMasker (Smit et al.), and searched for identical substitutions in the same genomic region. We iteratively screened genomic intervals of 15 nucleotides

immediately upstream and downstream of each confident RDD, these intervals being extended until no more additional edits could be rescued.

Cohort-wise integration of editing sites. Per-sample edits identified as described in the previous sections were then pooled together and fraction of edited bases for each sample was recomputed directly from pileup alignment in each samples in the cohort. This allowed rare edits detected only in highly covered transcriptomes to be rescued in other samples.

Excluded regions. The majority of the RDDs (10,254) clustered in 8.6Mb spread across the following five regions that did not overlap the Alu regions.

**Name**	**Chrom**	**Strand**	**txStart**	**txEnd**
BL_1	chr6	*	28477796	33448353
uc010yts.2	chr2	+	89890561	90471176
uc021vkt.1	chr2	-	89156873	89630175
uc021vku.1	chr2	+	89185067	89595920
uc021ser.1	chr14	-	105994255	107283085
uc021wml.1	chr22	+	22385571	23265082

These five regions encompassed immunoglobulin variable regions and HLA genes, suggesting alignment artifacts and/or a potential immunological effect unrelated to A-to-I editing. Therefore, we did not consider these RDDs, but used them as negative controls in several of our A-to-I editing analyses.

DNA-free A-to-I editing detection pipeline. we downloaded a list of known A-to-I editing sites for hg19 from the RADAR database (Ramaswami and Li, 2014). REDItoolKnown, which is part of the REDItools package (Picardi and Pesole, 2013), was then invoked with default settings for each of the 68 RNA-seq samples (`REDItoolKnown.py -i sample.bam -f hg19.fa -l RADAR.tab -u -o sample.edits.txt`). The output was a list of positions of known editing sites for which editing is callable, but not necessarily present, in the sample at hand. Per-sample lists of edits were merged, resulting in a total of 115,087 non-redundant genomic positions callable in at least one sample. The number of sites for which at least one read across the entire cohort documented an editing event was 59,993. Importantly, this pipeline did not use our DNA exome sequences, and therefore is not limited by the small fraction of the genome they cover—dramatically extending the number of callable basis. Among the 59,993 events, 50,918 were from Alu regions.

Measure of AZIN1 editing in tissues with the Roche FLX sequencer. A region containing the edited site of *AZIN1* was amplified using designed fusion oligonucleotide primers (Forward 5'-ACCGGAAGTGATGAACCAGCCT-3' and Reverse 5'-GCTGAATGCAAGAAGGCACAAAGA-3' specific sequences). For each patient sample, PCR was performed on 50 ng of cDNA using the Platinum PCR System (Life Technologies Europe B.V., Gent, Belgium) and standard Touch-Down thermocycling conditions (2 min denaturation at 94°C, followed by 20 cycles of denaturation for 30 s at 94°C, annealing for 30 s at 65°C* and extension for 30 s at 72°C (*with decrements of 0,5c° annealing temperature at the completion of each cycle), 20 cycles of denaturation for 30 s at 94°C, annealing for 30 s at 55°C and extension for 30 s at 72°C, and final extension for 6 min at 72°C). The fused primers

each contained a common 20-bp region at their 5'-end that is used in Multiplex Identifiers labeling, clonal amplification and sequencing on a 454 Genome Sequencer FLX system as described by manufacturer (Roche Applied Sciences, Indianapolis, USA).

After removing primer and adapter sequences, 454 reads were mapped on the reference genome (hg19) using the BLAT program (Kent, 2002) due to its ability to handle long spliced reads. Blat was invoked with the following command: '`blat -stepSize=5 hg19.fa reads.fasta out.psl`'. Editing at position chr8:103,841,636 was then computed for each sample based on pileup alignment.

Protein expression, mRNA expression and DNA copy number profiling

ADAR IHC. For each sample, a representative FFPE block containing invasive adenocarcinoma, including whenever possible a corresponding ductal carcinoma *in situ*-component, lymphocytes and normal ductal epithelium cells, was selected.

ADAR IHC was performed as follows: briefly, sections were de-paraffinized and processed using the Ventana detection system with the iView™ DAB detection kit (Ventana, Tucson, Arizona). Antigen retrieval was performed with EDTA (Tris/borate/EDTA; pH 8.4). The slides were then incubated in a 1:50 dilution of mouse polyclonal anti-ADAR antibody (Abcam, ab88574) at room temperature for 28 minutes. After staining, slides were processed in accordance with routine protocols. A representative slide was chosen and scanned with a NanoZoomer 2.0RS scan (Hamamatsu Photonics Hamamatsu-SHI, Japan) in 40x mode using the NDP.scan software.

Quantitative real-time PCR (qRT-PCR). In order to analyze the expression of *ADAR* p110, *ADAR* p150, total *ADAR* expression in both clinical samples and cell lines, we first reverse-transcribed 500 ng of total RNA using the High Capacity RNA-to-cDNA kit (Applied Biosystems, Foster City, CA) following the manufacturer's instructions. qRT-PCR was performed according to the TaqMan Gene Expression Assay protocol (Applied Biosystems) using the following primers: *ADAR* p110: forward, 5'-GGCAGTCTCCGGGTG -3', reverse 5'- CTGTCTGTGCTCATAGCCTTGA-3', FAM probe: 5'-CCGGCCGTGTCCCGAGGA-3'; *ADAR* p150: forward, 5'-CTTCCAGTGCGGAGTAGCG-3', reverse 5'- GTGACGGTGTCTGCTTTCCA-3', FAM probe: 5'- TCGGGCCAGGGTCGTGCC- 3'. For the quantification of total *ADAR*, we used commercially available primers and probe (Hs00241666_m1, Life Technologies). *GUSB* (Hs99999908_m1, Life Technologies) and *TBP* (Hs00427621_m1, Life Technologies) were used as reference genes. Real-time PCR was performed on a 7900HT Sequence Detection System (Applied Biosystems). All reactions were run in duplicate.

Gene expression from RNA-seq data. RNA-seq data were generated and aligned on the human genome as described in previous sections. Expression was then estimated from the BAM files using Cufflinks v2.0.0 (Trapnell et al., 2012) with options `-N -u --GTF`. The transcript database provided with the `--GTF` option was ENSEMBL GRCh37.65. The expression FPKM (Fragment Per Kilobase per Million aligned reads) values, x, generated by Cufflinks were set to non null values and $\log_2$-transformed with the formula $f(x) = \log_2(x+1)$.

Gene expression from Affymetrix® array. 100 ng of total RNA was profiled using the Affymetrix® HG-U133 Plus 2.0 Arrays (Affymetrix®, Santa Clara, California), following the manufacturer's instructions. Briefly, the RNA was first reverse-transcribed into double-stranded cDNA. This cDNA was transcribed in vitro. After

purification of the aRNA, 12.5 µg were fragmented and labeled prior to hybridization to the arrays. Quality control (QC) for each chip was performed following the recommendations posted on http://www.arrayanalysis.org/.

CEL files were normalized with fRMA (McCall et al., 2010) v1.8.0 for R (R Development Core Team) v2.15.1. Probes were annotated from the ENSEMBL transcript database (same version as above) using BioMart (Smedley et al., 2009) v2.12.0. The best probe for a given gene was selected with Jetset (Li et al., 2011) v0.99.3.

When needed, RNA-seq and Affymetrix data were matched gene-wise on the basis of HUGO gene symbols.

Genome Wide SNP analysis. Genome wide SNP analysis was performed at AROS Applied biotechnologies a/s (Aarhus, Denmark) on Affymetrix Genome-Wide Human SNP Arrays 6.0 (Affymetrix) following the manufacturer's instructions. Briefly, 500 ng of genomic DNA was digested with either Nsp I or Sty I and then ligated to adapters that recognize the cohesive four-basepair (bp) overhangs. A generic primer that recognizes the adapter sequence was used to amplify adapter ligated DNA fragments, with PCR conditions optimized to preferentially amplify fragments in the 200 to 1,100 bp size range in a GeneAmp PCR System 9700 (Applied Biosystems). After purification and quantification, a total of 45 µl of PCR product was fragmented and a sample of the fragmented product was visualized on a 4% TBE agarose gel to confirm that the average size was smaller than 180 bp. The fragmented DNA was labeled with biotin and hybridized to the GeneChip Mapping Panels for 18 hrs. Arrays were washed and stained using an Affymetrix fluidics Station 450 and scanned using a GeneChip Scanner 3000 7G (Affymetrix). The Affymetrix GeneChip Operating Software (GCOS) was used to collect and extract feature data from the Affymetrix GeneChip Scanner.

The Affymetrix Genome-Wide Human SNP 6.0 arrays were normalized for technical variation between chips using the copy number workflow of Affymetrix Power Tools release v1.14.3. We used the full version of the CDF, version na.32 of NetAffx's annotation database for SNP 6.0 and version na.32 r1 of the HapMap 270 reference file. We ran the procedure with the default parameter settings. The raw $\log_2$ ratios from above were segmented using the circular binary segmentation algorithm (Olshen et al., 2004) implemented in the R/Bioconductor package DNAcopy version v1.34.0. We applied the full permutation method with default parameter settings, except `undo.splits="sdundo", undo.SD=2`. The segmented $\log_2$ ratios were used as input to a two-level hierarchical mixture model as described by van de Wiel et al. (van de Wiel et al., 2007) and implemented in the R package CGHcall version v2.20.0. Default parameter settings were used expect for `prior="not all", nclass=4`.

Cell lines experiments

Cell culture and Interferon treatment. MCF7 (ATCC® HTB22™), MDA-MB-231 (ATCC® HTB26™), BT-474 (ATCC® HTB20™), MCF-10A (ATCC® CRL10317™), ZR-75-1 (ATCC® CRL1500™), BT-20 (ATCC® HTB19™) and HCC1569 (ATCC® CRL2330™) breast cells lines were obtained from ATCC (Manassas, Virginia) in December 2012 and cultured under standard conditions. All cell lines were regularly authenticated by morphological observation and tested for mycoplasma contamination (MycoAlert, Rockland, Maryland) before performing the

experiments described below. The cells were incubated at 37 °C in a humidified incubator containing 5% CO_2.

MCF7 and ZR-75-1 are ER+, HER2- tumor cell lines; MDA-MB-231 and BT-20 are ER- HER2- tumor cell lines; BT-474 is an ER+ HER2+ tumor cell line and HCC1569 is HER2+ ER-. MCF-10A is an immortalized, non-transformed mammary epithelial cell line. Where indicated, cell lines were treated with the following doses of interferon (IFN): 1000UI/ml of Universal Type I IFN (Recombinant Human IFN-alpha A/D [BglII]) (IFN-α; cat# 11200-1, R&D Systems, Minneapolis, Minnesota), 1000UI/ml of Recombinant Human IFN-beta 1a (Mammalian) (IFN-β; cat# 11415-1; R&D Systems) or 500UI/ml of Recombinant Human IFN-gamma (IFN-γ; cat# 285-IF-100; R&D Systems). Cells were treated for 24h (1 day), 48h (2 days) and 120h (5 days); parallel cultures were left untreated as controls.

Lentiviral transduction. ADAR gene expression inhibition was performed using transduction-ready lentiviral particles containing 3 target-specific constructs encoding shRNA specifically designed to knock down ADAR expression. Control shRNA lentiviral particles containing a scrambled shRNA were used as a negative control for experiments. MDA-MB-231, MCF7 and BT474 cells transduction were performed accordingly to manufacturer's instructions (Santa Cruz biotechnology, Texas).

Cell proliferation assay. Cell proliferation was determined by 3-(4,5-dimethylthiazole-2-yl)-2.5 diphenyltetrazolium bromide assay (MTT, Sigma). All cells were seeded at a density of 6000 cells per well. At each time point, 25 µl of 5 mg/ml MTT was added and incubated at 37°C for 3.5 h and 100 µl DMSO was added to the wells. Every 24 hours, the rate of cellular proliferation was determined by measuring the absorbance at 590 nm. Cell growth curves were calculated as mean values after normalization to the absorbance at day 1 from 3 independent experiments comprising each six replicates. Difference in cell growth was considered as significant when $p<0.05$ according to a paired t test.

Apoptosis assessment. Apoptotic cell percentage was evaluated using the PE-Annexin-V Apoptosis Detection kit I (BD Pharmingen, San Diego, CA) following the manufacturer's instructions. Briefly, cells were double stained with Annexin V and 7-AAD and were then analyzed by flow cytometry. Apoptotic cells were defined as Annexin V positive cells including Annexin V^+/ 7-AAD^- cells (early apoptosis) and Annexin V^+/7-AAD^+ cells (late apoptose). Difference in apoptosis was considered as significant when $p<0.05$ according to a paired t test.

Western blot analysis. Cells were lysed in a buffer (NaCl 150mM, Tris-HCl 50mM, NP40 1%, SDS 20%, EDTA 5mM, protease inhibitors cocktail) at 4°C for 30 minutes. Protein concentrations were determined using the Pierce™ BCA Protein Assay kit (Thermo Scientific). Equal amounts of proteins (10µg) were separated on 4-12% Bis-Tris gels, transferred to nitrocellullose membranes, blocked with TBST buffer (50 mM Tris pH 8.0, 150 mM NaCl, 0.1% Tween 20) containing 5% nonfat milk, washed with TBST buffer, and incubated overnight at 4°C with primary antibodies against ADAR1 antibody (Cat#12317S, Cell Signaling, Danvers, Massachusetts) at a dilution of 1:1000, and against Actin, Clone 4 (Cat# MAB1501R, Millipore, Billerica, Massachusetts), at a dilution of 1:5000. The membranes were then washed in TBST four times, incubated with HRP-conjugated secondary antibodies for 2 h at RT and washed in TBST buffer four times. Proteins were detected using the Western lightning Ultra system (Perkin Elmer, Waltham, Massachusetts). The immunoblot signals were visualized with a chemiluminescence system (Biorad, Hercules, California) and quantified using Biolab 4.0.1 software.

qRT-PCR analysis. The extraction, quantification and quality control of the RNA extracted from cell lines was performed as described above. Only four cell lines gave enough quality and quantity material for downstream analyses (MCF7, MDA-MB-231, BT-474, and MCF-10A). For the analysis of the data obtained with qRT-PCR, relative expression of the genes of interest to *GUSB* and *TBP* was calculated using the $2^{-\Delta Ct}$ method. This normalized expression level allowed to determine the fold changes in the expression of the genes of interest between different subgroups.

Processing of Roche FLX read mapping. Only four cell lines gave enough quality and quantity of material for downstream analyses (MCF7, MDA-MB-231, BT-474, and MCF-10A). Sequencing was performed as explained for the *AZIN1* amplicon. The following primers were used:

AZIN1ex11-13_F (Tag: AAGACTCGGCAGCATCTCCA; Specific Sequence: ACCGGAAGTGATGAACCAGCCT);

AZIN1ex11-13_R (Tag: GCGATCGTCACTGTTCTCCA; Specific Sequence: GCTGAATGCAAGAAGGCACAAAGA);

BPNT1_Alu1_F (Tag: AAGACTCGGCAGCATCTCCA; Specific Sequence: CCAATTGACAGTTCAGGTCAATGTTC);

BPNT1_Alu1_R (Tag: GCGATCGTCACTGTTCTCCA; Specific Sequence: AAAATTGTGCCCTAAAGAAATCTGG);

MRPS16_Alu_F (Tag: AAGACTCGGCAGCATCTCCA; Specific Sequence: TTCCCATGTGTTTTAAAAGCCTGAA);

MRPS16_Alu_R (Tag: GCGATCGTCACTGTTCTCCA; Specific Sequence: GCCAAATTATGTAATGTTTTCTTTTTC);

BPNT1_Alu2_F (Tag: AAGACTCGGCAGCATCTCCA; Specific Sequence: GCCGAGTTCCAGAATCTATTAAAAATG);

BPNT1_Alu2_R (Tag: GCGATCGTCACTGTTCTCCA; Specific Sequence: TCTTTCTCCTAGCTAAGTAAATGAAACTT);

ZDHHC20_Alu_F (Tag: AAGACTCGGCAGCATCTCCA; Specific Sequence: AAATCACTTTTCATTCACCCAATAAA);

ZDHHC20_Alu_R (Tag: GCGATCGTCACTGTTCTCCA; Specific sequence: GGCCAAATTATAAACAAATTATAAACCT).

A total of 39 samples, each corresponding to a specific combination of cell line, interferon treatment and duration, were multiplexed on a single 454 sequencing run. We first extracted per-sample amplicons and trimmed MID sequences at each ends of sequencing reads. During this step, we required that the read sequence start by the MID and ends by its reverse complement, for a total read length of 250bp, primer sequences included. This ensures that most reads retained for alignment correspond to amplicons sequenced at full length. Adaptor and primer sequences were further removed and reads were mapped on the reference genome with the bwasw (v0.5.9) aligner (Li and Durbin, 2010) with default parameters.

Once reads were mapped on the reference genome, edited positions were identified based on pileup alignment. Only target regions were screened for RNA editing. Mapped bases at each position were obtained using the SAMtools (v0.1.16) mpileup program (Li et al., 2009) called with the following command line: '`samtools mpileup -B -D -r range -q 0 -Q 0 -f hg19.fa aln.bam > out.pileup`', where range corresponds to regions targeted for amplicon sequencing. Since read depth was greatly superior compared to our whole

transcriptome datasets, we considered a position as edited if the number of non-reference bases was at least 10, so that low frequency editing events could be detected. Additionally, as target regions correspond to UTRs of genes transcribed on the reverse strand, we restricted identification of editing to positions where the reference base was a T and non-reference bases were Cs.

To investigate the incidence of coverage over the detection of RNA editing, we estimated the mean number of detected edits for different read depth. We first examined in what extend alignments could be downsampled. The only genomic region having a very high coverage (> 2,000 reads) in every sample was the *Alu* region located within *MRPS16* gene (chr10:75008708-75008970). For each sample, we then generated 10 replicates of the original alignment at specified depth *D* by randomly selecting *D* reads mapped on *MRPS16 Alu* region. We computed the mean number of edits detected across all downsampled replicates based on pileup alignment (10 or more edited bases at a given position). Note that this downsampling makes sense only for amplicon sequenced at full length and covering the whole target region, since in this case, number of mapped reads and read depth are equivalent.

Long 454 amplicons allow for the analysis of editability on a per-read basis. The main hypothesis is that if a given genomic position P_i is more often edited than another position P_j located in the same region (and consequently covered by the same amplicon sequence), then we can expect that P_j will be edited in amplicons where P_i has already been edited. In other words, if a given amplicon harbors a total of *N* edited positions, these should correspond to the *N* most edited positions across all amplicon sequences covering this region.

To verify this hypothesis, we extracted amplicon sequences mapped on each target *Alu* and overlapping all editable positions detected within this *Alu*. We then determined which positions were edited for each amplicon individually. This produces a binary matrix *M* for each target Alu, where $M_{i,j} = 1$ if position *i* is edited in amplicon *j*, *0* otherwise. Reads were further ordered from the highest edited to the one harboring the lowest number of edited bases. Similarly, genomic positions were also sorted from the most edited to the least edited.

Statistical Analysis

All computations were implemented in R (R Development Core Team) v2.15.1 and Bioconductor (Gentleman et al., 2004) 2.10. Defaults functions' parameters were used unless specified otherwise.

Third party data. Our analysis rests on a number of public domain data sets:

- *Alu* were located from the RepeatMasker (Smit et al.) downloaded from UCSC (http://genome.ucsc.edu).
- The DARNED database (Kiran and Baranov, 2010) for hg19 was downloaded from darned.ucc.ie.
- The RNA editing sites from the GM12878 lymphoblastoid were obtained from the Supplemental table of Ramaswamy et al. (Ramaswami et al., 2012).
- RNA-seq data from the TCGA were downloaded from the public access repository on 08/02/2013 and assembled using custom R scripts whilst CBS segmented $\log_2$ ratios for matching samples were downloaded.

Calculation of editing frequencies. For all RDD sites determined by the pipeline described in Figure S1, editing frequency was defined for each sample as the ratio of

the number of RNA-seq reads documenting the non-reference base by the total number of reads covering the site. Since gene expression varied from sample to sample, some RDD sites were not covered in some samples. In such cases, the editing frequency was considered undefined (i.e., 'NA' in R's terminology).

Statistical tests and related graphics. Spearman correlation coefficients, ρ, and corresponding p-values were calculated with R's cor.test function. All group comparisons were evaluated with the Wilcoxon tests as implemented by R's wilcox.test. All tests were two-sided, except for the paired comparison, which were single-sided. The multivariate analyses were performed with R's lm. Correlations coefficients and p-values were rounded to the nearest one significant digit number with R's signif. Because cancer is a heterogeneous disease, revealing the variability of statistics is essential. We displayed all individual sample-level data points for almost all the analyses conducted in this study in the form of scatter plots or strip charts with overlaid boxplots. Numerical data underlying each plot are provided as Supplemental Tables.

Confirmation of editing sites. Since our sequencing protocol was unstranded, RDDs were considered confirmed in the DARNED database if we could find a RDD in DARNED with the same genomic position and the same or reverse complement substitution.

The genomic positions of all putative RDDs and 1,000 random positions within 1,000 randomly selected *Alu* were sent to the Sanger Institute team (A.S. and P.C.). No other information was provided in order to avoid confirmation bias. They then computed DNA and RNA allelic frequencies at these putative RDD and random control positions in 15 breasts cancers. Tumor DNA sequences are described elsewhere (Nik-Zainal et al., 2012a, 2012b). RNA sequences were obtained from 2x75bp paired-end HiSeq 2000 Illumina sequencing with each sample run on two lanes. RNA-seq reads were aligned with TopHat (Trapnell et al., 2009, 2012) 1.3 in unsupervised mode (i.e., no transcript database provided to guide the alignment). The resultant binary alignment files (BAM) were merged then duplicates were removed with Picard (http://broadinstitute.github.io/picard/) MarkDuplicates. Multi-mapped reads were excluded from this analysis. SAMtools (Li et al., 2009) and a custom script were used to determine the allelic frequencies of each putative RDD. Hence, the data generation and computational processing of the validation samples differed substantially from those used to derive the original RDDs, reinforcing the value of the confirmation. The computed allelic frequencies were sent back to the Brussels team for comparison to the original RDDs. A RDD was considered confirmed if there was at least one sample with 1) a coverage >20 reads in both DNA and RNA at the RDD position, 2) an alternative allele identical to the original RDD in >2% of the RNA-seq reads, 3) The reference allele present in >95% on the DNA reads.

Editing frequencies heatmap. Rows and columns were ordered by increasing row-wide and column-wise mean editing frequencies. Contour lines were drawn from the smoothing of the resulting frequencies matrix. Smoothing was computed with the image.smooth function from R's fields package v6.7 with scale parameter theta=3.

Logistic dose-response fitting. Dose-response curves were established from the site-specific editing frequencies shown in Figure 5F and matched ADAR protein expression shown in Figure 5E (data in Supplemental Table S6). We included the two ADAR isoforms as (p150 + p110)/actin. We filtered out sites with less than 4 data points and for which the editing frequency at the lower ADAR expression was below 0.025. This filter excluded from the analysis sites for which trivial detection artifacts

caused departure from the logistic model. The dose-response curve of each editing site was then fit to the model with the `drm` function (with argument `fct= L.5(fixed=c(-1,0,NA,NA,1))`, i.e. the two-parameters logistic model, $f(x)=\varepsilon_i/(1+\exp(\omega_i-x))$) from R package drc (Ritz and Streibig, 2005) v2.5.12.

DNA-based statistical model of editability. RNA editing sites from biological sample GM12878 (see 'Third party data' above) were filtered to retain editing events falling within Alu regions and covered at a depth >20x. 51,621 sites passed this filter. They were ordered by order of appearance in the human genome. The first half of them were assigned to the training set, the second half to the validation set. Note that because editing sites tend to cluster per *Alu*, assigning them randomly to the training and validation sets would not guaranty independence of these sets.

The training set was used to derive DNA sequence features associated with the RNA editing ratio. We found highly significant association with the following variables defined on a per-site basis:

- Smith-Waterman alignment score of the 51bp hg19 DNA sequence centered on the edit site within the 2,501bp DNA sequence, also centered on the edit site, but on the opposite strand. We computed the alignment with the pairwiseAlignment function from Bioconductor package GenomicRanges v1.8.13 using the local-global mode with mismatch penalty of -3 and default parameters of function nucleotideSubstitutionMatrix.
- The distance between the best Smith-Waterman alignment and the edit site.
- The 20 nucleotides surrounding the edit site.

The edit ratios in the training set were then modeled with these 1+1+20=22 variables using a linear model as implemented by R's lm function. We attempted to use alternative parameters, e.g. larger windows around the edited sites and compute models with RBF kernel support vector machines, but did not obtain radically better fits of the training data.

Finally, the linear model was used to estimate the editability scores of the validation editing sites shown in Figure 4J.

Gene set analysis. We derived the genes whose expression had a strong positive correlation with the mean editing frequency by taking the intersection of the 250 genes the most positively correlated (Spearmans' ρ) with the mean editing frequency in the RNA-seq expression data, and the Affymetrix expression data. The intersection contained 85 genes.

The Affymetrix expression values were adjusted for *ADAR* amplification with a procedure akin to that of a previous publication (Venet et al., 2011). For each gene, expressions were fitted to the level of *ADAR* amplification determined from our Affymetrix SNP6.0 arrays. *ADAR* CN-adjusted expressions were then computed for each gene as the sum of its mean expression across the cohort and the residuals of the fit.

Adjusted Affymetrix data were then analyzed with the GSA (Efron and Tibshirani, 2007) package v1.03 for R, first using the 'canonical pathway' and then the 'transcription factor targets' gene sets from MSigDB (Liberzon et al., 2011; Subramanian et al., 2005) v3.1 (files c2.cp.v3.1.symbol.gmt and c3.tft.v3.1.symbol.gmt downloaded from www.broadinstitute.org/gsea). We searched for gene sets correlated with the patient-averaged editing frequency (using GSA's `resp.type="Quantitative"`).

Supplemental References

Allinen, M., Beroukhim, R., Cai, L., Brennan, C., Lahti-Domenici, J., Huang, H., Porter, D., Hu, M., Chin, L., Richardson, A., et al. (2004). Molecular characterization of the tumor microenvironment in breast cancer. Cancer Cell *6*, 17–32.

Choudhury, S., Almendro, V., Merino, V.F., Wu, Z., Maruyama, R., Su, Y., Martins, F.C., Fackler, M.J., Bessarabova, M., Kowalczyk, A., et al. (2013). Molecular Profiling of Human Mammary Gland Links Breast Cancer Risk to a p27(+) Cell Population with Progenitor Characteristics. Cell Stem Cell *13*, 117–130.

Denkert, C., Loibl, S., Noske, A., Roller, M., Müller, B.M., Komor, M., Budczies, J., Darb-Esfahani, S., Kronenwett, R., Hanusch, C., et al. (2010). Tumor-associated lymphocytes as an independent predictor of response to neoadjuvant chemotherapy in breast cancer. J. Clin. Oncol. Off. J. Am. Soc. Clin. Oncol. *28*, 105–113.

Eisenberg, E., Adamsky, K., Cohen, L., Amariglio, N., Hirshberg, A., Rechavi, G., and Levanon, E.Y. (2005). Identification of RNA editing sites in the SNP database. Nucleic Acids Res. *33*, 4612–4617.

Elston, C.W., and Ellis, I.O. (1991). Pathological prognostic factors in breast cancer. I. The value of histological grade in breast cancer: experience from a large study with long-term follow-up. Histopathology *19*, 403–410.

Genestie, C., Zafrani, B., Asselain, B., Fourquet, A., Rozan, S., Validire, P., Vincent-Salomon, A., and Sastre-Garau, X. (1998). Comparison of the prognostic value of Scarff-Bloom-Richardson and Nottingham histological grades in a series of 825 cases of breast cancer: major importance of the mitotic count as a component of both grading systems. Anticancer Res. *18*, 571–576.

Gentleman, R.C., Carey, V.J., Bates, D.M., Bolstad, B., Dettling, M., Dudoit, S., Ellis, B., Gautier, L., Ge, Y., Gentry, J., et al. (2004). Bioconductor: open software development for computational biology and bioinformatics. Genome Biol. *5*, R80.

Harvey, J.M., Clark, G.M., Osborne, C.K., and Allred, D.C. (1999). Estrogen receptor status by immunohistochemistry is superior to the ligand-binding assay for predicting response to adjuvant endocrine therapy in breast cancer. J. Clin. Oncol. Off. J. Am. Soc. Clin. Oncol. *17*, 1474–1481.

Karolchik, D., Hinrichs, A.S., Furey, T.S., Roskin, K.M., Sugnet, C.W., Haussler, D., and Kent, W.J. (2004). The UCSC Table Browser data retrieval tool. Nucleic Acids Res. *32*, D493–D496.

Kent, W.J. (2002). BLAT--the BLAST-like alignment tool. Genome Res. *12*, 656–664.

Leake, R., Barnes, D., Pinder, S., Ellis, I., Anderson, L., Anderson, T., Adamson, R., Rhodes, T., Miller, K., and Walker, R. (2000). Immunohistochemical detection of steroid receptors in breast cancer: a working protocol. UK Receptor Group, UK

NEQAS, The Scottish Breast Cancer Pathology Group, and The Receptor and Biomarker Study Group of the EORTC. J. Clin. Pathol. *53*, 634–635.

Li, H., and Durbin, R. (2010). Fast and accurate long-read alignment with Burrows-Wheeler transform. Bioinforma. Oxf. Engl. *26*, 589–595.

Li, H., Handsaker, B., Wysoker, A., Fennell, T., Ruan, J., Homer, N., Marth, G., Abecasis, G., and Durbin, R. (2009). The Sequence Alignment/Map format and SAMtools. Bioinforma. Oxf. Engl. *25*, 2078–2079.

Li, Q., Birkbak, N.J., Gyorffy, B., Szallasi, Z., and Eklund, A.C. (2011). Jetset: selecting the optimal microarray probe set to represent a gene. BMC Bioinformatics *12*, 474.

Liberzon, A., Subramanian, A., Pinchback, R., Thorvaldsdóttir, H., Tamayo, P., and Mesirov, J.P. (2011). Molecular signatures database (MSigDB) 3.0. Bioinforma. Oxf. Engl. *27*, 1739–1740.

McCall, M.N., Bolstad, B.M., and Irizarry, R.A. (2010). Frozen robust multiarray analysis (fRMA). Biostat. Oxf. Engl. *11*, 242–253.

McKenna, A., Hanna, M., Banks, E., Sivachenko, A., Cibulskis, K., Kernytsky, A., Garimella, K., Altshuler, D., Gabriel, S., Daly, M., et al. (2010). The Genome Analysis Toolkit: a MapReduce framework for analyzing next-generation DNA sequencing data. Genome Res. *20*, 1297–1303.

Nik-Zainal, S., Van Loo, P., Wedge, D.C., Alexandrov, L.B., Greenman, C.D., Lau, K.W., Raine, K., Jones, D., Marshall, J., Ramakrishna, M., et al. (2012b). The life history of 21 breast cancers. Cell *149*, 994–1007.

Olshen, A.B., Venkatraman, E.S., Lucito, R., and Wigler, M. (2004). Circular binary segmentation for the analysis of array-based DNA copy number data. Biostat. Oxf. Engl. *5*, 557–572.

Picardi, E., and Pesole, G. (2013). REDItools: high-throughput RNA editing detection made easy. Bioinformatics. *29*, 1813–1814.

R Development Core Team R: A Language and Environment for Statistical Computing. *1*, ISBN 3–900051 – 07–0.

Ritz, C., and Streibig, J. (2005). Bioassay Analysis Using R. J. Stat. Softw. *12*.

Sherry, S.T., Ward, M.H., Kholodov, M., Baker, J., Phan, L., Smigielski, E.M., and Sirotkin, K. (2001). dbSNP: the NCBI database of genetic variation. Nucleic Acids Res. *29*, 308–311.

Smedley, D., Haider, S., Ballester, B., Holland, R., London, D., Thorisson, G., and Kasprzyk, A. (2009). BioMart--biological queries made easy. BMC Genomics *10*, 22.

Smit, A., Hudley, R., and Green, P. RepeatMasker Open-3.0.

Subramanian, A., Tamayo, P., Mootha, V.K., Mukherjee, S., Ebert, B.L., Gillette, M.A., Paulovich, A., Pomeroy, S.L., Golub, T.R., Lander, E.S., et al. (2005). Gene set enrichment analysis: a knowledge-based approach for interpreting genome-wide expression profiles. Proc. Natl. Acad. Sci. U. S. A. *102*, 15545–15550.

Trapnell, C., Pachter, L., and Salzberg, S.L. (2009). TopHat: discovering splice junctions with RNA-Seq. Bioinforma. Oxf. Engl. *25*, 1105–1111.

Trapnell, C., Roberts, A., Goff, L., Pertea, G., Kim, D., Kelley, D.R., Pimentel, H., Salzberg, S.L., Rinn, J.L., and Pachter, L. (2012). Differential gene and transcript expression analysis of RNA-seq experiments with TopHat and Cufflinks. Nat. Protoc. *7*, 562–578.

Venet, D., Dumont, J.E., and Detours, V. (2011). Most random gene expression signatures are significantly associated with breast cancer outcome. PLoS Comput. Biol. *7*, e1002240.

Van de Wiel, M.A., Kim, K.I., Vosse, S.J., van Wieringen, W.N., Wilting, S.M., and Ylstra, B. (2007). CGHcall: calling aberrations for array CGH tumor profiles. Bioinforma. Oxf. Engl. *23*, 892–894.

Wolff, A.C., Hammond, M.E.H., Schwartz, J.N., Hagerty, K.L., Allred, D.C., Cote, R.J., Dowsett, M., Fitzgibbons, P.L., Hanna, W.M., Langer, A., et al. (2007). American Society of Clinical Oncology/College of American Pathologists guideline recommendations for human epidermal growth factor receptor 2 testing in breast cancer. J. Clin. Oncol. Off. J. Am. Soc. Clin. Oncol. *25*, 118–145.
